# Unlocking the Synergistic Promoter Role of Phosphorus in Evolving NiFe Phosphides for Enhanced Water Oxidation

**DOI:** 10.1007/s40820-026-02238-0

**Published:** 2026-06-11

**Authors:** Ningning Shi, Mingcheng Gao, M. Maneesha, C. S. Praveen, Panpan Liu, Shengnan Yue, Wangjing Xie, Dechao Chen, Yu Tang, Yuanqing Wang, Hua Fan, Xing Huang

**Affiliations:** 1 State Key Laboratory of Green and Efficient Development of Phosphorus Resources, College of Chemistry, Fuzhou University, 350108 Fuzhou, People’s Republic of China; 2 Electron Microscopy Center, Qingyuan Innovation Laboratory, 362100 Quanzhou, People’s Republic of China; 3 School of Chemistry, Chemical Engineering and Materials, Jining University, 273155 Qufu, People’s Republic of China; 4 International School of Photonics, Cochin University of Science and Technology, 682022 Cochin, India; 5 Materials Genome Institute, Shanghai University, 200444 Shanghai, People’s Republic of China

**Keywords:** NiFeP, Structural transformations, (oxy)Hydroxides, Oxygen evolution reaction, Density functional theory calculations

## Abstract

Transition metal-based electrocatalysts, especially NiFe (oxy)hydroxides, represent state-of-the-art systems for the alkaline oxygen evolution reaction (OER). While structural evolution of electrocatalysts under working conditions has garnered considerable attention, as the evolved structure often dictates catalytic activity, the mechanisms driving these transformations and catalytic performance remain poorly understood. In this work, using NiFe phosphide (NiFeP) as a model system, we systematically elucidate how phosphorus guides irreversible structural transformations and boosts OER performance. Through a combination of electron microscopy, spectroscopy, and electrochemical analysis, we demonstrate that phosphorus facilitates the transformation of NiFeP into NiFe (oxy)hydroxides while retaining residual oxyanion moieties. Importantly, we reveal that phosphorus not only accelerates the active phase formation but also suppresses Fe dissolution and directly modulates the electronic structure, a dual function that has not been well-documented in the literature. The reconstructed catalyst exhibits outstanding OER activity and remarkable stability under alkaline conditions, achieving a low overpotential of 225 mV at 10 mA cm^−2^ and stably maintaining a current density of 500 mA cm^−2^ for over 100 h, ranking among the most effective OER catalysts reported in the literature. Density functional theory calculations indicated that the resulting PO_4_^3−^ and Fe act synergistically to regulate the redox chemistry of Ni, preventing Ni over-oxidation and enhancing electronic conductivity. Furthermore, PO_4_^3−^ stabilizes key OER intermediates and lower the thermodynamic energy barrier, thereby improving catalytic performance.

**Supplementary Information:**

The online version contains supplementary material available at 10.1007/s40820-026-02238-0.

## Highlights

- Phosphorus drives NiFeP’s reconstruction into active NiFe (oxy)hydroxide, suppresses Fe dissolution, and modulates Ni’s electronic structure via residual PO_4_^3−^.
- PO_4_^3−^ and Fe synergistically act as a redox buffer, preventing Ni over-oxidation, narrowing the bandgap, and stabilizing key oxygen evolution reaction intermediates to lower the energy barrier.
- The restructured catalyst achieves a low overpotential of 225 mV at 10 mA cm^−2^ and maintains stable operation in alkaline media for over 100 h at current densities up to 500 mA cm^−2^.

**Supplementary Information:**

The online version contains supplementary material available at 10.1007/s40820-026-02238-0.

## Graphical abstract

**Figure:**
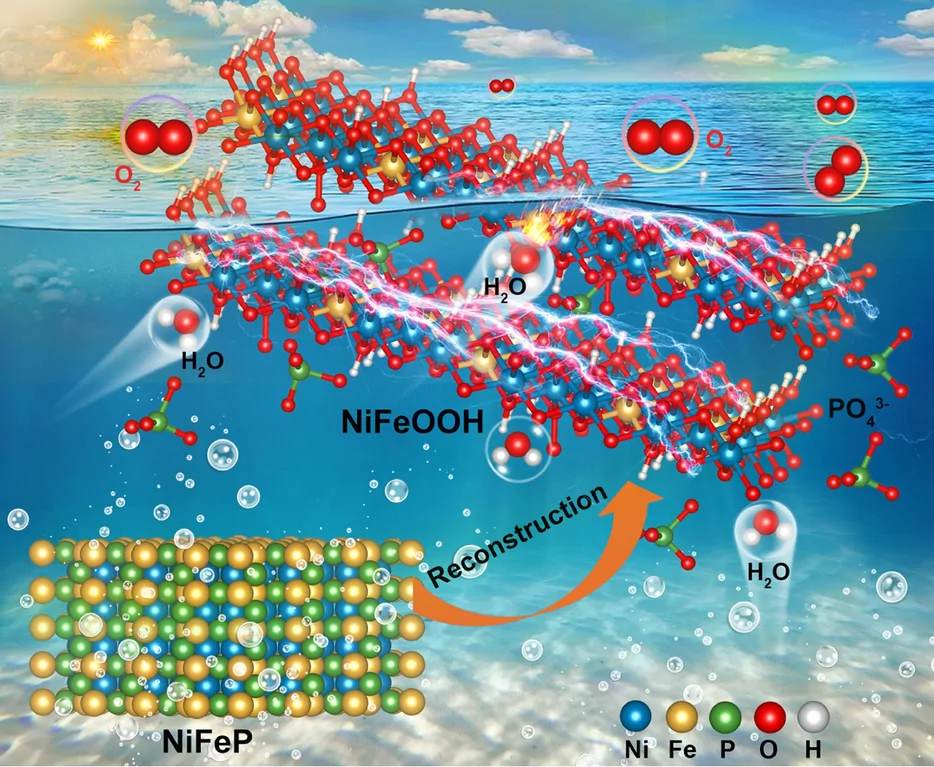

## Introduction

The rapid growth of the global economy has led to the gradual depletion of traditional fossil fuels, such as petroleum, coal, and natural gas, prompting an urgent need for alternative clean energy sources [1, 2]. While solar and wind power have emerged as cost-effective solutions, they are non-dispatchable and require grid-scale energy storage solutions to accommodate fluctuations in energy supply/demand [3, 4]. Among the most promising candidates, hydrogen stands out as a potential solution for such grid-scale energy storage and is already a major commodity chemical [5–7]. While hydrogen can be produced via electrochemical water splitting, the efficiency of this process is limited by the sluggish anodic oxygen evolution reaction (OER), which involves a complex four-electron transfer mechanism, breaking O–H bonds, and the formation of an O–O bond [8, 9].

Currently, high-performance OER catalysts primarily comprise noble-metal-based materials such as RuO_2_ and IrO_2_. However, their limited availability and high cost significantly hinder widespread commercialization. In contrast, earth-abundant first-row transition metal-based catalysts (e.g., Fe, Co, and Ni) can exhibit appreciable OER activity under alkaline conditions [10–16]. Recently, transition metal-based phosphides, sulfides, and selenides (collectively referred to as metal X-ides) have emerged as promising alternatives to conventional noble metal oxide electrocatalysts due to their high activity [17–19].

Among the metal X-ides, NiFe-based phosphides are particularly attractive for the OER due to their high intrinsic activity, favorable electronic structure, and cost-effectiveness [20–25]. To further enhance their activity and durability, various strategies have been explored. For instance, bulk amorphous NiFeP materials have been developed that exhibit excellent OER performance, due to their high macroscopic conductivity and abundant surface-active sites [26]. Specifically, such materials achieve a low overpotential of 219 mV at a current density of 10 mA cm^−2^ in an alkaline electrolyte [26]. Moreover, advanced architectural designs have been successfully fabricated, including 3D ternary nickel–iron phosphides with hierarchically porous microflower-like structures [27], NiFeP coupled with carbon tubes [28, 29], NiFeP anchored on Ti_3_C_2_T_x_ MXene [30], and NiFeP nanoflakes integrated with metal–organic frameworks (MOFs) derived from CoP-nitrogen-doped carbon [31], all delivering superior OER performance.

However, recent evidence increasingly suggests that the as-synthesized metal X-ides typically exhibit low structural stability and often function as pre-catalysts. Under anodic conditions, these materials tend to undergo structural transformations, oxidizing to the corresponding metal oxides [32], hydroxides [33, 34], or (oxy)hydroxides [35]. It has since been recognized that these in situ formed metal (oxy)hydroxides actually offer enhanced OER performance compared to their precursor states, other transformed states, or thermally prepared oxides [35–37]. For instance, Fan et al. observed an irreversible surface transformation of CoS_x_ into crystallized CoOOH, accompanied by a morphological change via a Co(OH)_2_ intermediate during the OER. This transformation, captured by in situ TEM and FTIR, indicates that CoOOH is the active species in the OER process [35]. Similarly, Hu et al. found that, driven by the anodic potential and the presence of hydroxide anions in solution, lattice sulfur atoms on the surface of (NiCo)S_1.33_ particles can be replaced by oxygen. This substitution results in both lattice oxygen and sulfur coexisting on/in the surface, a phenomenon also observed in nickel selenide and nickel telluride [38]. The resulting oxygenated surface reduces the energy barrier for subsequent reconstruction into active NiCo (oxy)hydroxides [37]. Likewise, in the case of NiFeP, the Ni_5_P_2_/FeP_4_ nanoboxes were found to undergo reconstruction into defective NiOOH/FeOOH nanosheets, forming hierarchical nanoboxes with superior OER activity and stability, benefiting from abundant interfaces between NiOOH and FeOOH and plentiful defects [36]. The surface defects generated by the etching of NiFeP have been found to promote surface reconstruction, leading to the formation of oxyhydroxide species [39]. Moreover, the self-reconstructed layer tends to form a stable (Ni_1−x_Fe_x_)_3_P–O/NiOOH heterostructure, which effectively suppresses the dissolution of phosphorus and iron [40]. Particularly, the ligand effect of phosphorus has been explained to modulate the metal electronic structure, thereby breaking the scaling relations for the adsorbed OER intermediates energetics. This modulation resulted in lower OER overpotentials and enhanced stability [41, 42].

While the critical role of structural evolution during electrochemical reactions is recognized, the atomistic-level mechanisms by which anions such as phosphorus direct this process and enhance catalytic performance remain elusive [17, 43–45]. Unlike previous reports that primarily consider phosphorus as a leachable template or a simple electronic modifier, herein we utilize NiFeP as a model system to comprehensively investigate its multifaceted roles. Herein, we utilize NiFeP as a model system to investigate the multifaceted roles of phosphorus. We demonstrate that phosphorus is not merely a sacrificial template that facilitates reconstruction into the active NiFe (oxy)hydroxide phase, but more importantly, its residual oxyanions (PO_4_^3^^–^) synergistically cooperate with Fe to modulate the electronic structure of Ni via a redox‑buffering mechanism. This synergistic effect functions as an intrinsic redox buffer and is pivotal for the observed exceptional activity and stability. By combining identical-location structural and chemical composition tracking, ex situ structural and chemical state analyses, we provide direct insight into the complex chemical‑structural transformations of metal phosphides during electrochemical water oxidation. Our study goes beyond the conventional understanding of phosphides as pre‑catalysts and offers valuable perspectives on the dynamic structure–performance relationship of metal phosphides in the OER, paving the way for the rational design of anion‑engineered electrocatalysts.

## Experimental Section

### Materials

All chemicals were used as received without further purification. Polyvinylpyrrolidone (PVP, M_w_ = 29,000), K_3_[Fe(CN)_6_] (99.9%) and NaH_2_PO_2_ (98%-101%) were purchased from Shanghai Macklin Biochemical Technology Co., Ltd. Ni(CH_3_COO)_2_·4H_2_O (≥ 98.0%); KOH (≥ 85%) and anhydrous ethanol (99.7%) were purchased from Sinopharm Chemical Reagent Co., Ltd. Commercial RuO_2_ (98%) was purchased from Shanghai Adamas Reagent Co., Ltd. Nafion (5 wt% D-520) was purchased from Dupont China Holding Co., Ltd. Carbon fiber paper was purchased from Toray Industries, Inc. Deionized water was purchased from Hangzhou Wahaha Co., Ltd. Argon (99.999%) was purchased from Fuzhou Yuanhua Chemical Co., LTd.

### Preparation of Pre-Ni, Pre-NiFe, NiFeP, Ni_x_P_y_, and NiFeO

#### Preparation of the Ni Precursor Prisms (Denoted as Pre-Ni)

Typically, 4.5 g of PVP and 2.1 g of Ni(CH_3_COO)_2_·4H_2_O were dissolved in 300 mL of anhydrous ethanol at ambient temperature to generate a clear green solution in a 500-mL round-bottom flask. The solution was then heated to 90 ℃ and refluxed under continuous magnetic stirring for 5 h. The obtained products were centrifuged, washed with anhydrous ethanol, and finally dried at 60 °C in a vacuum overnight.

#### Preparation of the NiFe Prisms (Denoted as Pre-NiFe)

20 mg of the pre-Ni was re-dispersed in 5 mL of ethanol to prepare solution A. 30 mg of K_3_[Fe(CN)_6_] was first dissolved into 5 mL of deionized (DI) water and 5 mL of ethanol to get a transparent solution, referred to as solution B. Solution A was then mixed with solution B, and the mixture was aged for 24 h at room temperature. The obtained yellow products were centrifuged by washing with anhydrous ethanol, and finally dried at 60 °C in a vacuum overnight.

#### Preparation of the Hollow NiFeP Prisms (Denoted as NiFeP)

In a typical synthesis, 20 mg of pre-NiFe and 200 mg of NaH_2_PO_2_ were placed at opposite ends of a porcelain boat, with NaH_2_PO_2_ (0.1–2.0 g) positioned upstream of the tube furnace. The phosphidation was then carried out at 350 °C for 2 h under an argon atmosphere, with a heating rate of 5 °C min^–^^1^. Upon cooling to room temperature, the NiFeP were collected. Based on our preliminary OER tests (Fig. S1 and Table S1), the optimal amount of NaH_2_PO_2_ was determined to be 0.2 g.

#### ***Preparations of the Ni***_x_***P***_y_*** (Denoted as Ni***_x_***P***_y_***)***

The synthesis process was similar to that of NiFeP, with the only difference being the use of a pre-Ni in place of pre-NiFe for the subsequent phosphidation.

#### Preparations of the NiFeO (Denoted as NiFeO)

The entire synthesis process was similar to that of NiFeP, except that annealing was performed under air conditions, and the phosphidation treatment was not applicable.

### Characterizations

X-ray diffraction (XRD) patterns were measured on a Rigaku Ultima Ⅳ diffractometer with Cu Kα radiation (λ = 1.5418 Å). Scanning electron microscopy (SEM) images were acquired using a Verios G4 UC scanning electron microscope at an accelerating voltage of 20 kV. Transmission electron microscopy (TEM), high-resolution TEM (HRTEM) images, and energy-dispersive X-ray spectroscopy (EDX) data were recorded on a FEI Talos F200s G2 with an accelerating voltage of 200 kV. Inductively coupled plasma optical emission spectrometry (ICP-OES) was performed by Agilent ICP-OES 720EX. Inductively coupled plasma mass spectrometry (ICP-MS) measurements were analyzed by Agilent 7800. X-ray photoelectron spectrometer (XPS) measurements were performed with an ESCALAB 250 by using Al/Mg as the excitation source. The XPS fitting was conducted after background subtraction (Shirley type). No constraints on peak positions were imposed during the fitting. The mixed Gaussian–Lorentzian line shape was adopted during the deconvolution with a fixed mixing factor (L:G = 30%). Ni K-edge X-ray absorption fine structure (XAFS) was performed with Si (111) crystal monochromators at the BL14W Beamline at the Shanghai Synchrotron Radiation Facility (SSRF) in Shanghai, China. The XAFS spectra were recorded in transmission mode at room temperature. The recorded data were processed using the software code Athena.

### Identical Location Transmission Electron Microscopy (IL-TEM)

For IL-TEM investigations, the catalyst (1 mg) was dispersed in 200 μL of alcohol. Subsequently, a 10 μL suspension was drop-cast onto a gold finder grid coated with a carbon-supported film. The gold finder grid was then placed onto a PEEK-embedded glassy carbon (GC) electrode with a diameter of 3 mm and covered with a Teflon cap with a hole diameter of 2.7 mm. Electrochemical measurements were performed in a single-chamber, three-electrode setup, where the working electrode was a catalyst-coated gold finder grid with a carbon film, mounted on the GC electrode. The reference electrode used was Hg/HgO, while a graphite rod was employed as the counter electrode.

## Results and Discussion

### Design Principle and Structural Characterizations

**Fig. 1:**
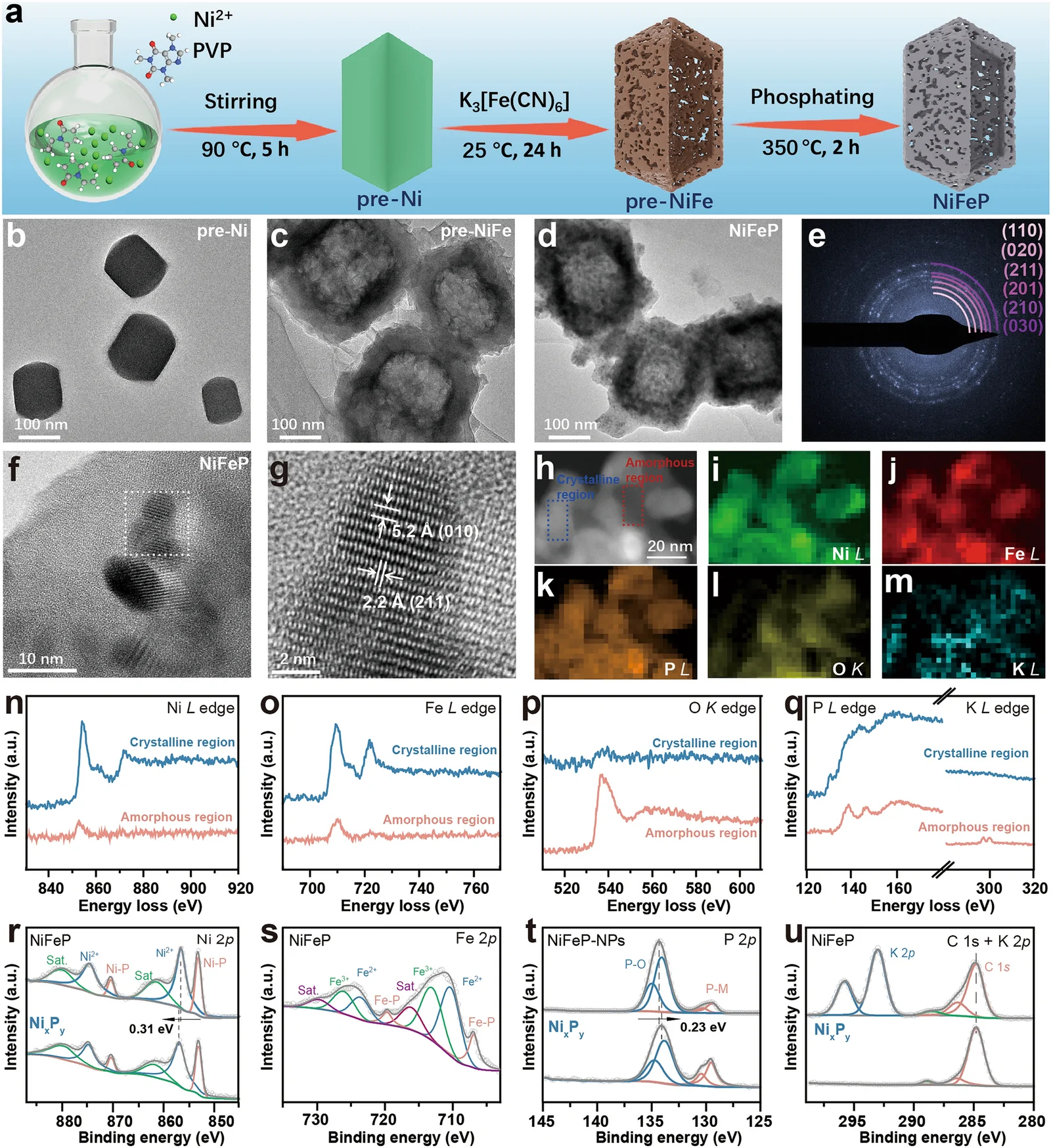
Characterizations of the as-synthesized materials. **a** Scheme illustration of the fabrication processes of NiFeP. TEM images of **b** pre-Ni, **c** pre-NiFe, and **d** NiFeP. **e** SAED patterns of NiFeP. **f** HRTEM and **g** enlarged HRTEM images for the marked area indicated in **f**. **h-m** HAADF-STEM image and corresponding EELS mappings of NiFeP. **n-q** EELS spectra of different elements involved in NiFeP for crystalline and amorphous regions. **r** Ni 2*p*, **s** Fe 2*p*, **t** P 2*p*, and **u** K 2*p *XPS spectra of NiFeP and Ni_x_P_y_

NiFeP materials were synthesized through a multi-step process. Initially, nickel precursors (pre-Ni) were fabricated as sacrificial templates using a facile reflux method [46]. During the subsequent chemical transformation, the [Fe(CN)_6_]^3−^ complex progressively consumed the pre-Ni templates, releasing Ni^2+^ ions into the solution. These Ni^2+^ ions subsequently facilitated the co-precipitation of Fe^3+^ ions. As the inner Ni core was gradually dissolved, a hollow-structured NiFe precursor (pre-NiFe) formed. Finally, the pre-NiFe was converted into hollow-structured NiFeP via a gas-phase phosphidation treatment (Fig. 1a). The fabrication process is schematically illustrated in Fig. 1a. The Ni/Fe/P atomic ratio in the NiFeP was determined to be approximately 3:1:4, as measured by ICP-OES (Table S2).

The morphology, structure, and chemical composition of the as-prepared pre-Ni, pre-NiFe, NiFeP, NiFeO, and Ni_x_P_y_ were investigated by SEM, TEM, and XRD. The SEM and TEM images indicated that the pre-Ni materials have a smooth surface and appear in a prism-like shape (Figs. S2a, S3, and 1b). The XRD pattern demonstrated that the crystallographic structure of the pre-Ni prisms was similar to that of tetragonal nickel acetate hydroxides (Fig. S2b) [46]. After coordination with K_3_[Fe(CN)_6_], hollow pre-NiFe prisms with rough surface were formed, as depicted in Figs. S4a, S5, and 1c. The XRD pattern of the hollow pre-NiFe prisms can be assigned to nickel–iron cyanide hydrate (Fig. S4b) [36]. After phosphidation, the as-obtained hollow NiFeP prisms largely retained the morphology and size of the pre-NiFe prisms, albeit with rougher surfaces and the appearance of dark-contrast spots within the prisms (Figs. S6a and 1d). Selected area electron diffraction (SAED) analysis reveals ring patterns of diffraction spots (Fig. 1e), which can be assigned to a metal phosphide phase (M_2_P) (PDF No. 89–4864). The HRTEM imaging further reveals that the obtained NiFeP prisms consist of crystalline nanoparticles surrounded by amorphous materials (Fig. 1f). Analysis of the enlarged HRTEM image and the fast Fourier transform (FFT) of the crystalline particles (Fig. 1g) indicates that they are characteristic of metal phosphide (M_2_P) with a hexagonal structure, consistent with SAED and XRD results (Figs. 1e and S6b). The observed lattice fringes with *d*-spacings of 2.2 and 5.2 Å can be assigned to the (2–11) and (010) planes of Ni_2_P, consistent with the crystal phase observed in XRD. Electron energy-loss spectroscopy study further reveals that the crystalline particles comprise Ni and Fe, along with P, indicating the formation of Ni_x_Fe_y_P (Fig. 1h-m and n-q). Notably, the Ni and Fe signals did not uniformly overlap (Fig. 1i, j), indicating a varied compositional distribution of these elements within the crystalline particles. In contrast, the amorphous regions contain Ni, Fe, O, P, and K, aligning with the EDX analysis (Fig. S7). Similar amorphous materials have been identified in other metal phosphide nanoparticles [47–49]. The additional presence of K can be attributed to the Fe precursor used in the synthesis of pre-NiFe, which contains K_3_[Fe(CN)_6_]. As control samples, Ni_x_P_y_ was synthesized via phosphidation of the pre-Ni (Figs. S8 and S9), while NiFeO was also prepared for comparative analysis (Figs. S10 and S11).

Furthermore, XPS was employed to analyze the surface chemical states of Ni, Fe, and P elements in hollow NiFeP prisms and Ni_x_P_y_. As depicted in Fig. 1r, the Ni 2*p* core level spectrum of NiFeP prisms can be deconvoluted into Ni–P (853.2/870.3 eV) in Ni_x_Fe_2-x_P [50], Ni^2+^ in the oxidized Ni species (856.6/874.6 eV), and satellite peaks (861.4/880.1 eV) [51]. The XPS spectrum of Fe 2*p* in Fig. 1s positioned at 706.9/719.7 eV corresponds to Fe–P species in Ni_x_Fe_2-x_P (706.9/719.7 eV) [50], while the peaks at 709.8/723.2 eV and 711.9/724.7 eV are assigned to the Fe^2+^/Fe^3+^ species in the amorphous phase [52], respectively. Additionally, the P 2*p* peaks at 129.4/130.3 eV and 134.0/134.9 eV (Fig. 1t) are ascribed to P-metal and P-O species, respectively. It should be noted that the binding energy for Ni 2*p* in NiFeP prisms was negatively shifted, while P 2*p* was positively shifted compared to the value in Ni_x_P_y_, manifesting that the substitution of Fe indeed modulates the electronic structure of NiFeP prisms. As mentioned earlier, K was also present in the NiFeP materials, as confirmed by the detection of K 2*p* in Fig. 1u.

### Electrocatalytic OER Performance in Alkaline Water

After characterizing the morphology, structure, and composition, we proceeded to evaluate the OER performance of pre-NiFe, NiFeP, NiFeO, Ni_x_P_y_, and commercial RuO_2_ using a typical three-electrode electrochemical system in Ar-saturated 1.0 M KOH electrolyte. The potential measured against the Hg/HgO electrode was converted to the reversible hydrogen electrode (RHE) scale, with calibration applied to the Hg/HgO reference (see Fig. S12). All samples were subjected to CV (40 cycles, Fig. S13) and LSV measurements. Post-LSV characterization revealed that these treatments led to altered elemental composition, morphology, and structure of the as-prepared catalysts, except for NiFeO. This process, which induces significant structural and chemical modifications, will be discussed in the next section. Samples treated by CV and LSV are referred to as activated catalysts, denoted as pre-NiFe-A, NiFeP-A, NiFeO-A, and Ni_x_P_y_-A, respectively, where “A” denotes the activated state.

**Fig. 2:**
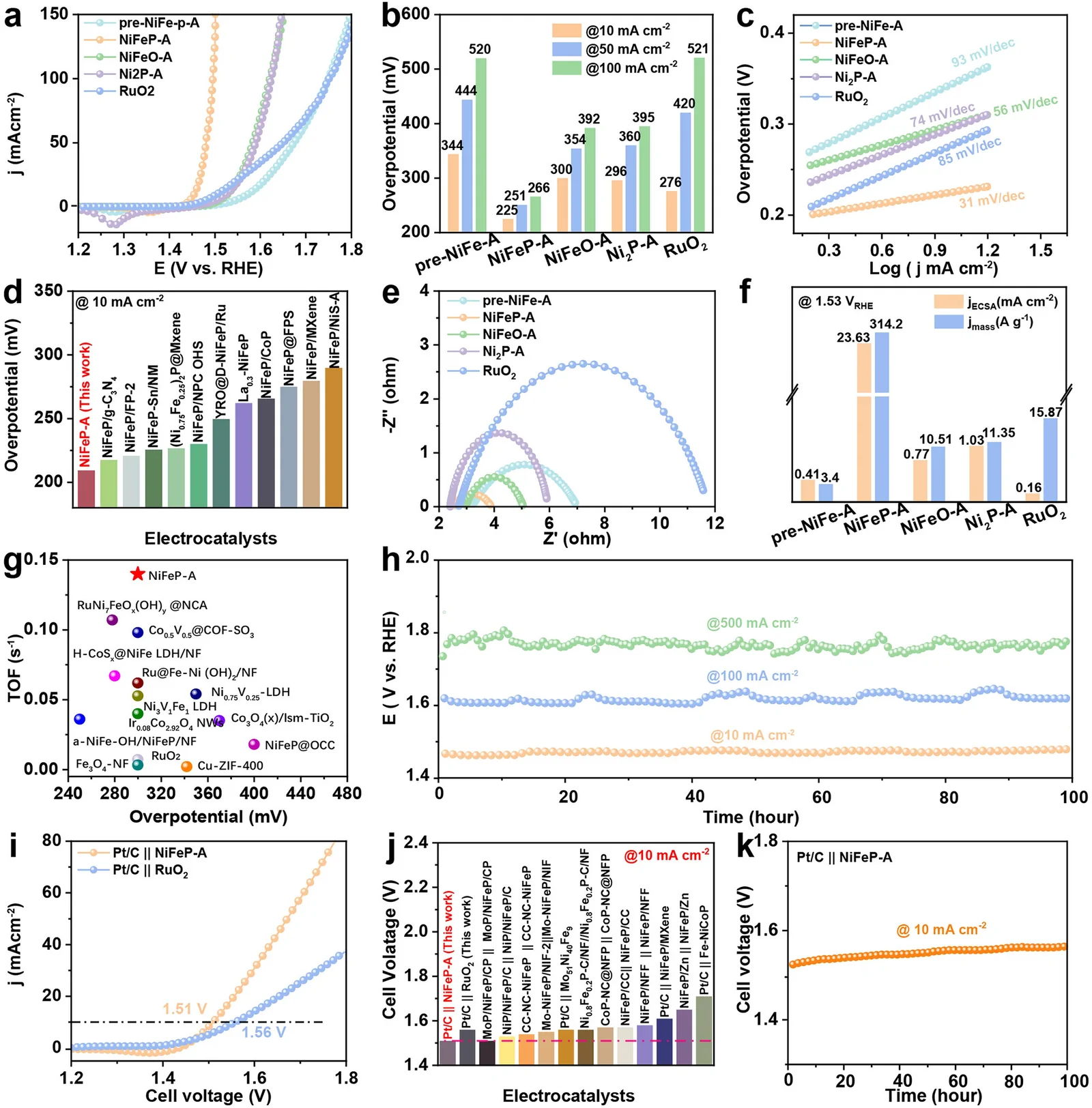
Catalytic performance evaluation. **a** Polarization curves (backward-scan profiles) of pre-NiFe-A, NiFeP-A, NiFeO-A, Ni_x_P_y_-A, and commercial RuO_2_ in 1.0 M KOH. **b** Overpotentials of different catalysts at current densities of 10, 50, and 100 mA cm^–2^. **c** Tafel plots of as-prepared catalysts recorded in 1.0 M KOH. **d** Comparison of overpotentials at 10 mA cm^–2^ with recently reported NiFeP-based OER catalysts in 1.0 M KOH. **e** The Nyquist plots. **f** ECSA-normalized specific activity (orange column) and mass activity (blue column) of various samples at 1.53 V *vs.* RHE. **g** Comparison of TOFs. **h** Stability test of NiFeP-A at the current densities of 10, 100, and 500 mA cm^–2^ for 100 h. **i** LSV curves of the Pt/C || NiFeP-A two electrode system and commercial Pt/C || RuO_2_ system in 1 M KOH solution. **j** Comparison of cell voltages at 10 mA cm^–2^ with state-of-the-art overall water splitting electrocatalysts in 1.0 M KOH. **k** Stability tests of the Pt/C || NiFeP-A two electrode system at a current density of 10 mA cm^–^^2^

As shown in Fig. 2a, b, the NiFeP-A exhibited superior OER activity, achieving a current density of 10 mA cm^–2^ at an overpotential of 225 mV, significantly outperforming pre-NiFe-A (344 mV), NiFeO-A (300 mV), Ni_x_P_y_-A (296 mV), and even RuO_2_ (276 mV). The enhanced catalytic activity is primarily attributed to the introduction of Fe and the phosphidation treatment. We further extended the LSV measurement of NiFeP‑A to current densities beyond 500 mA cm^–2^, and an overpotential of 500 mV was required to achieve 500 mA cm^–2^ (Fig. S14). This value is comparable to those of state‑of‑the‑art catalysts reported in the literature (Table S3).

The corresponding Tafel slopes, shown in Fig. 2c, revealed that NiFeP-A had the lowest Tafel slope of 31 mV dec^–^^1^, while the Tafel slopes of pre-NiFe-A, NiFeO-A, Ni_x_P_y_-A, and RuO_2_ were 93, 56, 74, and 85 mV·dec^–^^1^, respectively. This indicated that NiFeP-A exhibited superior OER kinetics than the other samples. Moreover, the NiFeP-A outperformed most other OER electrocatalysts reported in the literature (Fig. 2d, Table S4), highlighting its potential for practical applications. Furthermore, we applied electrochemical impedance spectroscopy (EIS) to measure the as-prepared electrocatalysts. As shown in Fig. 2e and Table S5, NiFeP-A (1.36 Ω) exhibited a lower charge-transfer resistance (R_ct_) than pre-NiFe-A (3.80 Ω), NiFeO-A (2.09 Ω), Ni_x_P_y_-A (3.55 Ω), and RuO_2_ (9.05 Ω), further demonstrating that the NiFeP-A had the fastest charge-transfer kinetics. The marked decrease in R_ct_ from Ni_x_P_y_-A to NiFeP-A further suggests that the addition of Fe significantly enhances electrical conductivity.

We also evaluated the ECSA by measuring the *C*_dl_ in non-Faradaic regions at different scan rates (Fig. S15). The higher ECSA value of NiFeP-A (9.13 cm^2^) indicated that it possessed a large number of exposed active sites. For a better comparison of the intrinsic activity of the as-prepared catalysts, the specific activity, mass activity, and TOF were examined at a potential of 1.53 V *vs.* RHE. Our results showed that NiFeP-A exhibited the highest specific activity (23.63 mA cm^–^^2^), mass activity (314.2 A g^–^^1^), and TOF value (0.14 s^–^^1^) among all other samples and recently reported catalysts (see Fig. 2f, g, Tables S6 and S7), indicating that NiFeP-A had a much higher intrinsic activity. In addition, we examined the stability of the NiFeP-A at a current density of 10, 100, and 500 mA cm^–2^. As shown in Fig. 2h, the overpotential did not exhibit significant degradation during chronopotentiometry tests over 100 h. Furthermore, the LSV curves of NiFeP-A before and after 10,000 cycles were nearly overlapping. These observations collectively demonstrate the high stability of the catalyst (Fig. S16).

To assess the practical potential of the as-prepared catalysts for overall water splitting, we employed a two electrode configuration in 1.0 M KOH, with NiFeP-A serving as the anode and commercial Pt/C (20 wt.%) as the cathode. For comparison, a system using commercial Pt/C and RuO_2_ was also evaluated. As depicted in Fig. 2i, the NiFeP-A || Pt/C couple required a cell voltage of only 1.51 V to achieve a current density of 10 mA cm^–2^, which is lower than that of the Pt/C || RuO_2_ benchmark (1.56 V). Remarkably, the performance of our NiFeP-A || Pt/C system surpasses that of most previously reported electrocatalysts (Fig. 2j and Table S8). Furthermore, the stability of the NiFeP-A || Pt/C system was examined at a constant current density of 10 mA cm^–2^. As shown in Fig. 2k, the cell voltage increased slightly from 1.525 to 1.565 V over 100 h of continuous operation, demonstrating good long-term durability. Additionally, we evaluated the performance of our catalyst system under higher current densities of 100 and 500 mA cm^–2^, which are more relevant to industrial operation conditions. As shown in Fig. S17, the cell voltage gradually increases over time at both current densities. This degradation is largely attributable to the limited stability of the commercial Pt/C cathode, as our chronopotentiometry tests (performed in a three-electrode configuration) confirms that the NiFeP catalyst alone can stably sustain the high current density of 100 and 500 mA cm^–2^ for over 100 h (Fig. 2h).

### Structural Transformations of NiFeP

**Fig. 3:**
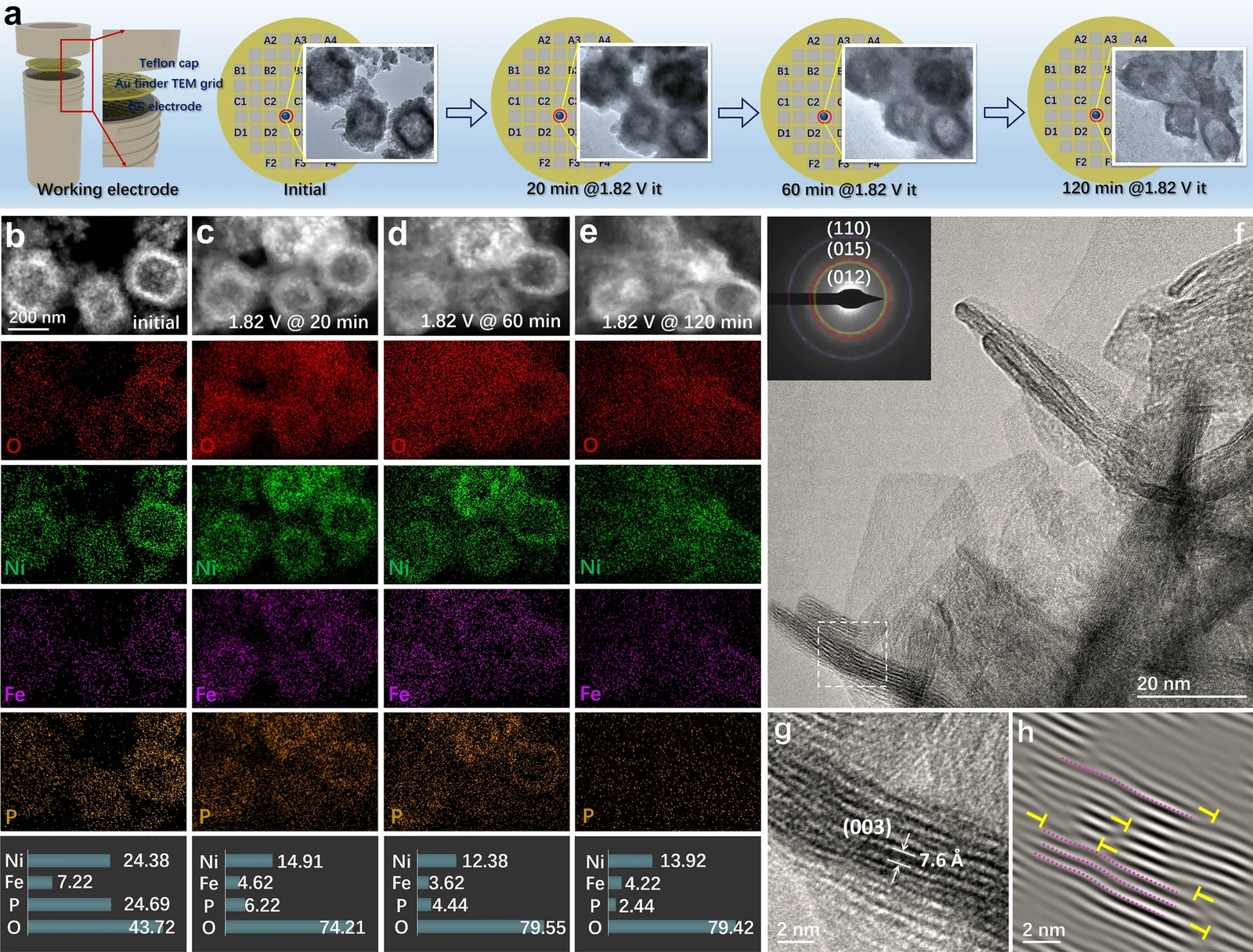
IL-TEM investigation. **a** Scheme of IL-TEM approach for electrochemical processes. HAADF-STEM images and corresponding elemental analysis of **b** initial NiFeP and after 1.82 V_RHE_ for **c** 20 min, **d** 60 min, and **e** 120 min electrolysis. **f** HRTEM image of NiFeP-A sample. **g** Enlarged HRTEM image from the dotted square indicated in **f**. **h** Inversed FFT image. The dotted magenta lines indicate distorted lattice fringes

The structural and chemical evolution of NiFeP during the anodic oxidation process was examined using IL-TEM, as schematically illustrated in Fig. 3a. After anodization at an applied potential of 1.82 V vs. RHE in 1.0 M KOH for varying durations, the NiFeP deposited on the Au-finder TEM grid was dried and immediately imaged via TEM. The pristine NiFeP prisms exhibited a hollow structure (Fig. 3b), consistent with the observations in Fig. 1c. With a chronoamperometry treatment lasting 20 min (Fig. 3c) and 60 min (Fig. 3d), the hollow progressively reconstructed into sheet-like structures, accompanied by a significant reduction in phosphorus content. After extended electrolysis for 120 min (Fig. 3e), the hollow prisms were further transformed into nanosheets, with the phosphorus content reduced to minimal levels, while the Ni/Fe ratio (Ni:Fe ≈ 3.3:1.0) remained largely unchanged. Additional representative regions are shown in Fig. S18, further confirming the above findings. ICP-OES analysis of NiFeP-A confirmed a significant decrease in phosphorus content, while the Ni/Fe ratio remained unchanged (Ni:Fe:P = 3.37:1.00:0.48, see Table S9). Consistently, ICP-MS analysis of the electrolyte before and after activation revealed ppm-level concentrations of Ni and Fe, indicating minimal dissolution of the transition metals (Fig. S19).

To further explore the origin of these changes, we conducted detailed electron microscopic analyses of the samples collected at each step of the activation process. TEM and EDX analyses of the CV-treated NiFeP (Fig. S20a-d) revealed significant morphological changes, with most areas transforming into nanosheets accompanied by partial dissolution of phosphorus. Nevertheless, the original morphology and phase structure were partially retained in certain regions (Fig. S20c), suggesting that the CV treatment did not induce a complete structural transformation. Following both CV and LSV treatments, electron diffraction analysis indicated the disappearance of metal phosphite phases, and the resulting ring patterns ((012), (015), and (110)) could be well indexed to NiFe-based hydroxide—a known layered structure (see insets in Fig. 3f) [53]. The diffuse character of the ring patterns suggested low crystallinity, which was further supported by HRTEM images showing curved lattice fringes (Fig. 3f-i). Structural analysis based on HRTEM revealed that the nanosheets resembled NiFe hydroxide, with lattice fringes exhibiting a *d*-spacing of 7.6 Å, corresponding to the (003) planes of NiFe hydroxide. Elemental mapping (Fig. S21) demonstrated spatially uniform distributions of O, Ni, Fe, and P across the entire sample. The homogeneous distribution of P indicated that P primarily existed within the interlayers of the nanosheets, likely in the form of PO_4_^3^^–^. The activation process resulted in a catalyst with abundant defects and a low-crystalline structure, both of which are associated with enhanced OER performance compared to directly synthesized counterparts [36]. These features may account for the high OER activity observed for the catalyst in this study.

### Chemical State and Coordination Environment

**Fig. 4:**
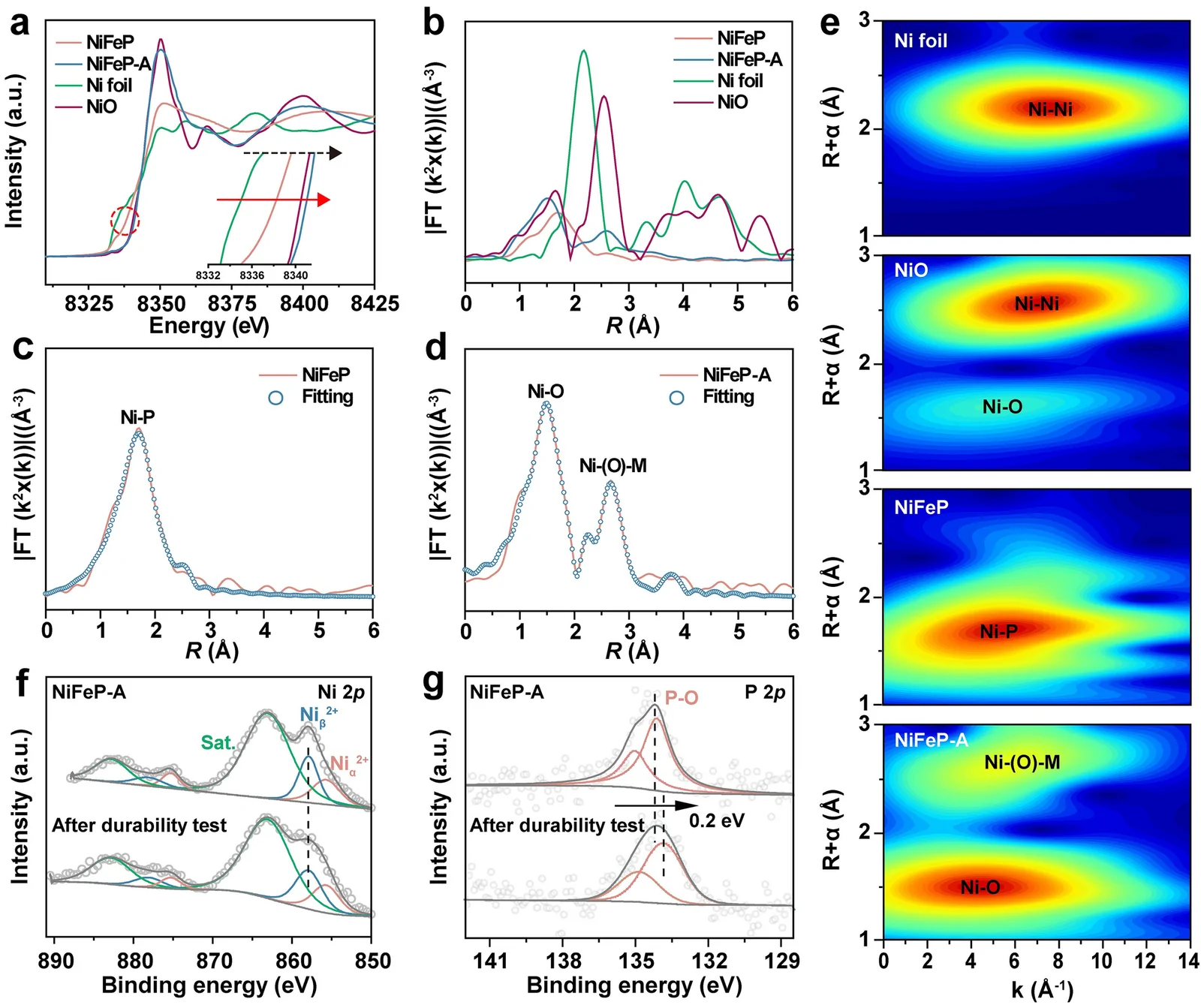
Electronic state and coordination environment. **a** XANES spectra of Ni K-edge. **b** FT-EXAFS spectra. The fitting of **c** NiFeP and **d** NiFeP-A. **e** WT analysis. All the FT-EXAFS data are presented without phase correction. **f, g** XPS spectra of NiFeP-A before and after stability test

The Ni K-edge XAFS was measured to track the changes in the electronic structure and coordination environment of Ni. The Ni K-edge X-ray absorption near-edge structure (XANES) spectra (Fig. 4a) indicated that the absorption edge of NiFeP was positioned between that of Ni foil and NiO, suggesting that the average oxidation state of Ni in NiFeP ranged from 0 to +2, consistent with the XPS results. After activation (NiFeP-A), the edge energy shifts to virtually coincide with that of NiO, signifying that the average oxidation state of Ni approaches +2. As shown in Fig. 4b, the FT-EXAFS spectra of NiFeP at the initial state exhibited a single peak around 1.71 Å, corresponding to Ni–P bonds in NiFeP [26, 54]. Upon activation, the peak intensity associated with Ni–P bonds in NiFeP disappears, where two new peaks emerge at 1.47 Å and 2.67 Å in NiFeP-A. These peaks can be assigned to the Ni–O and Ni-(O)-M bonds [55, 56], respectively, where M denotes a neighboring atom that is indistinguishable in XAS and can be either Ni or Fe. This variation suggests that the surface of NiFeP transforms into NiFe hydroxides and/or oxides or (oxy)hydroxides, which is consistent with the TEM observations (see Fig. 3). Fitting of the FT-EXAFS spectrum (Fig. 4c, d, Table S10) indicates coordination numbers of 7.1 ± 0.6 for pre-Ni in NiFeP, whereas in NiFeP-A, the coordination numbers for Ni–O and Ni-(O)-M (M = Ni or Fe) are approximately 7.3 ± 0.4 and 4.1 ± 0.2, respectively. This indicates that Ni is coordinated by O atoms instead of P atoms within the NiFeP-A. The combined TEM results and fitting analysis warrant the conclusion that NiFeP-A is characterized by a defect-rich NiFe (oxy)hydroxide structure. Wavelet transform EXAFS analyses further confirm the presence of Ni–O and Ni-(O)-M scattering paths at ≈4.5 and 5.7 Å^–1^, respectively, in NiFeP-A, which are different from those observed in Ni foil, NiO, and NiFeP (Fig. 4e).

XPS analysis was conducted to provide further insights into the changes in chemical composition and valence states of the catalysts following activation. As shown in Fig. 4f, the peaks assigned to Ni–P species in NiFeP disappear following reconstruction. Two distinct sets of peaks emerge at 855.7/875.3 eV and 857.9/878.1 eV. The first set corresponds to Ni^2+^ coordinated to lattice oxygen (denoted as Ni_α_^2+^). The second set, while appearing at binding energies close to those of Ni^3+^, is unlikely to originate from Ni^3+^, as this species is unstable and readily reduces to Ni^2+^ upon removal of the anodic potential [24]. Therefore, the second set is likely attributed to Ni^2+^ coordinated to oxyanions (denoted as Ni_β_^2+^) [57, 58], resulting from the residual presence of phosphate groups in the catalyst. For the Fe 2*p* peaks (Fig. S22), accurate fitting was challenging due to overlap with the Ni *LMM* Auger lines, the limited sample quantity used for post-reaction testing, and the partial dissolution of trace amounts of Ni and Fe into the electrolyte. Nonetheless, the data clearly demonstrate the disappearance of Fe–P species (initially at 129.4/130.3 eV) after the reaction (Figs. 4g and S22). Additionally, EELS analysis was performed to probe the electronic structures of NiFeP and NiFeP-A, as shown in Fig. S23. Compared to the crystalline regions of NiFeP, NiFeP-A shows decreased white line ratios for Ni *L*-edge and Fe *L*-edge, respectively, indicating increases in oxidation states of Ni and Fe following in situ activation [59], consistent with the XPS results. The NiFeP-A catalyst was further examined after a long-term stability test for 100 h using TEM and XPS. The morphology remained similar to that of NiFeP-A before the stability test, and EDX analysis confirmed the presence of P (0.55 at%) and its uniform distribution (Fig. S24). Moreover, XPS analysis confirmed the good stability of the activated catalyst (NiFeP-A). While minor shifts and changes in the spectra of P and Fe were observed, such as the slight decrease in the Fe 2*p* signal at around 720 eV (Fig. S22). These results collectively indicate that the overall chemical state remains largely unaltered after the stability test (Figs. 4f, g, and S22).

### Role of Phosphorus

The effects of phosphorus on the morphological and chemical composition variations upon the activation of NiFeP were investigated by comparing samples with and without phosphorus, such as Ni_x_P_y_, NiFeO, and pre-NiFe. The results demonstrate that Ni_x_P_y_ underwent a transformation process similar to that of NiFeP (Fig. S25), whereas no significant structural changes were observed in NiFeO (Fig. S26). These findings suggest that phosphorus plays a key role in promoting structural transformation. Notably, pre-NiFe underwent a complete transformation into ultrathin Ni (oxy)hydroxide (Fig. S27), accompanied by the near-total loss of Fe ions in the material, as confirmed by the detection of Fe ions in the electrolyte (Fig. S28). In contrast, Fe ions remained in both the NiFeP-A and NiFeO-A samples (Figs. S21 and S26), implying that either phosphidation or the heat treatment effectively prevented the dissolution of Fe ions.

To discern these two factors, pre-NiFe-Ar was prepared by annealing pre-NiFe in an argon atmosphere followed by XRD and TEM analysis (Figs. S29 and S30). The crystalline pre-NiFe-Ar after electrochemical activation (denoted as pre-NiFe-Ar-A) revealed no significant morphological or structural changes, and EDX analysis indicated that the initial atomic ratio of Ni:Fe was preserved after activation. These observations suggested that Fe ion preservation in pre-NiFe-Ar is primarily due to the heating treatment. Moreover, a comparison between pre-NiFe-Ar-A and NiFeP-A revealed that the introduction of phosphorus indeed induces distinct morphological changes (Figs. S31 and 3f). Additionally, pre-NiFe-Ar-A exhibits significantly lower OER activity than NiFeP-A (Fig. S32), further supporting the conclusion that the phosphidation is essential for enhancing electrocatalytic performance.

The enhancement of electrocatalytic performance can be attributed to the formation of the active NiFe (oxy)hydroxide structures promoted by the introduction of phosphorus. In addition, it can be linked to the presence of phosphorus (in the form of phosphate) directly in the electrocatalytic system formed upon activation. Our above characterizations revealed that NiFeP underwent significant phosphorus dissolution during activation, leaving only trace amounts of residual phosphate in the sample (see Table S9 and Fig. 3e). This residual phosphate may be adsorbed on the catalyst surface and/or intercalated into the sample, likely playing a crucial role in OER performance. Long-term stability experiments, which directly compare the NiFeP-A sample (containing residual phosphate, Fig. 2h) with the phosphorus-free NiFe-A (Fig. S33), further demonstrate the significant role of the residual phosphate in stabilizing the OER activity.

**Fig. 5:**
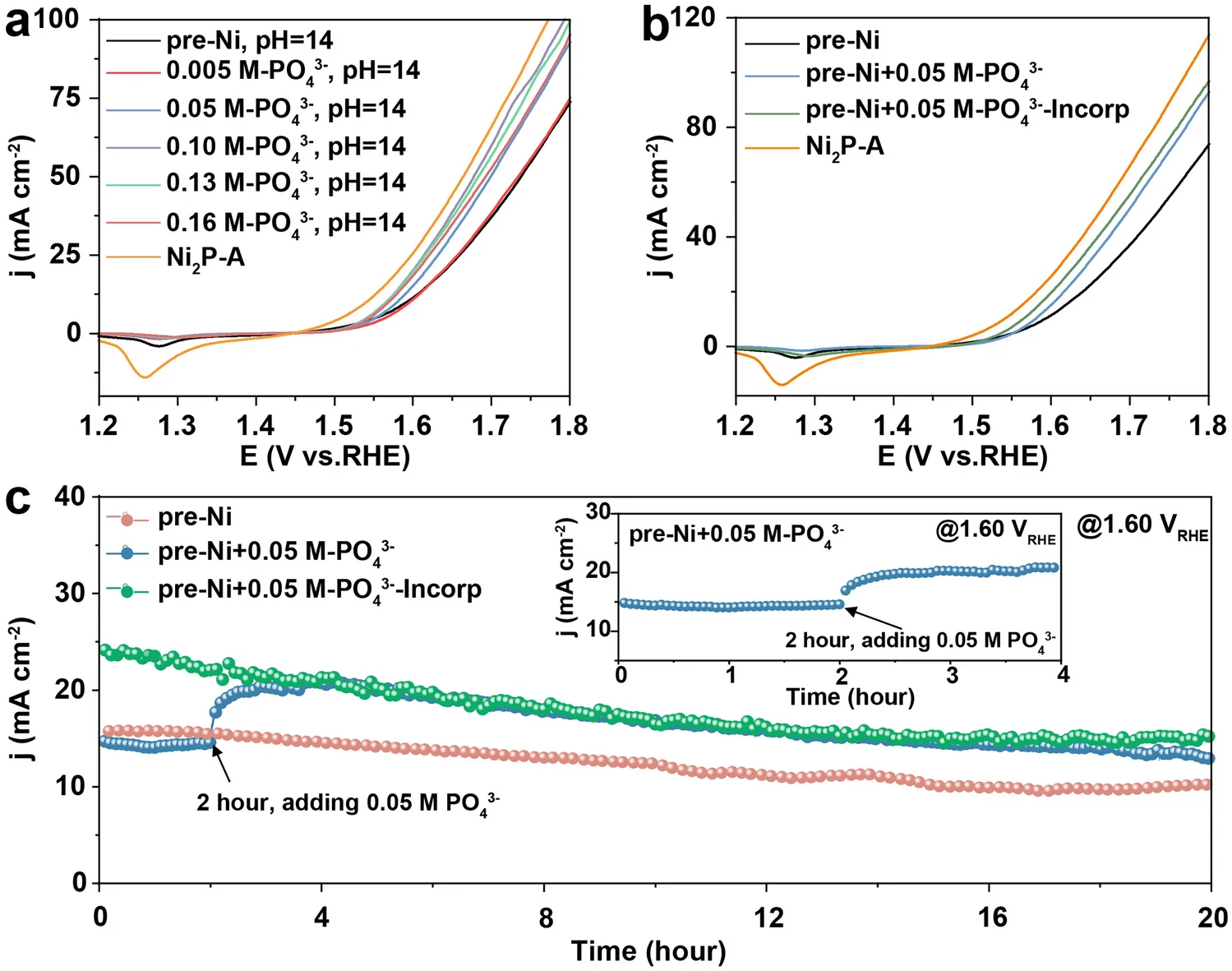
**a** LSV curves of Ni precursor in 1.0 M KOH electrolyte in the presence of different concentrations of PO_4_^3^^–^ and Ni_2_P-A. **b** Comparison of LSV curves of pre-Ni, pre-Ni + 0.05M_PO_4_^3^^–^, pre-Ni-PO_4_^3^^–^-Incorp, and Ni_2_P-A. **c** CA measurements of pre-Ni, pre-Ni + 0.05M_PO_4_^3^^–^, and pre-Ni-PO_4_^3^^–^-Incorp at 1.60 V vs. RHE

To further examine the role of phosphate in enhancing the OER performance, we measured the OER performance of the Ni precursor in 1.0 M KOH and that with the addition of PO_4_^3^^–^ using LSV and CA (Fig. 5a). Before adding PO_4_^3^^–^, the Ni precursor underwent the same CV and LSV treatments as described earlier. As shown in Fig. 5a, the LSV curves indicated that the addition of PO_4_^3^^–^ clearly enhances the OER activity of the Ni precursor. As the concentration of PO_4_^3^^–^ is increased from 0.005 to 0.05 M, the catalytic performance of the Ni precursor progressively improves, reaching optimal performance at a PO_4_^3^^–^ concentration of 0.1 M. Notably, the addition of PO_4_^3^^–^ to the 1.0 M KOH electrolyte had little effect on the pH, making it reasonable to attribute the enhanced activity to the PO_4_^3^^–^ rather than changes in pH. However, further increasing the PO_4_^3^^–^ concentration leads to decreased performance, suggesting that excess PO_4_^3^^–^ is detrimental and that an optimal synergy between PO_4_^3^^–^ concentration and the NiOOH host is required to achieve maximum catalytic performance. During the CA measurements, we applied ~ 1.60 V vs. RHE to the Ni precursor in fresh 1.0 M KOH for 2 h and recorded the initial OER activity (~ 15.7 mA cm^–^^2^). Afterward, 0.05 M PO_4_^3^^–^ was added to the electrolyte, resulting in an immediate and dramatic increase in OER current, which reached a maximum (~ 20.4 mA cm^–^^2^) after 2 h (Fig. 5c). This observation suggests that the PO_4_^3^^–^ within the sample (either via adsorption or intercalation) facilitates the OER kinetics.

We also assessed the role of phosphate by activating the pre-Ni in the presence of 0.05 M PO_4_^3^^–^ in 1.0 M KOH using CV and LSV treatments (Fig. 5b). This procedure likely facilitated the incorporation of phosphate into the bulk phase structure of the formed Ni oxyhydroxides, which was designated as “pre-Ni-PO_4_^3^^–^-Incorp,” in contrast to the previous case, activated without phosphate (designated as pre-Ni + 0.05M_PO_4_^3^^–^). However, it is expected that surface-adsorbed PO_4_^3^^–^ is also present in this scenario due to the release of PO_4_^3^^–^ into the electrolyte during activation. As shown in Figs. 5b and S34, pre-Ni-PO_4_^3^^–^-Incorp exhibited a lower overpotential and reduced R_ct_ compared to pre-Ni + 0.05M_PO_4_^3^^–^, suggesting that PO_4_^3^^–^ within the bulk phase enhanced electron transfer kinetics more effectively than surface-adsorbed PO_4_^3^^–^. Furthermore, Ni_x_P_y_-A exhibited superior activity compared to the two aforementioned cases, highlighting the significance of the phosphidation process in the evolution of the active structure. Additionally, CA measurements of pre-Ni-PO_4_^3^^–^-Incorp showed a higher initial current (~ 25 mA cm^–^^2^) compared to pre-Ni + 0.05 M_PO_4_^3^^–^ (15 mA cm^–^^2^), suggesting that PO_4_^3^^–^ incorporation into the bulk structure had a more pronounced activity-enhancing effect (Fig. 5c). Back to the CA experiment regarding the addition of PO_4_^3^^–^, it took nearly 2 h to reach the maximum activity after the addition. This delay may be attributed to the diffusion of PO_4_^3^^–^ from the electrolyte into the interlayers of the basal plane of Ni (oxy)hydroxides, resulting in a gradual enhancement of catalytic activity. After about 16 h of extended electrolysis, the current densities of both samples largely overlap, suggesting that prolonged electrolysis has brought the samples to a similar state (Fig. 5c). Collectively, these observations indicate that both adsorbed and intercalated PO_4_^3^^–^ are beneficial for enhancing OER performance, with intercalated PO_4_^3^^–^ most likely playing the dominant role.

### Density Functional Theory Calculations

**Fig. 6:**
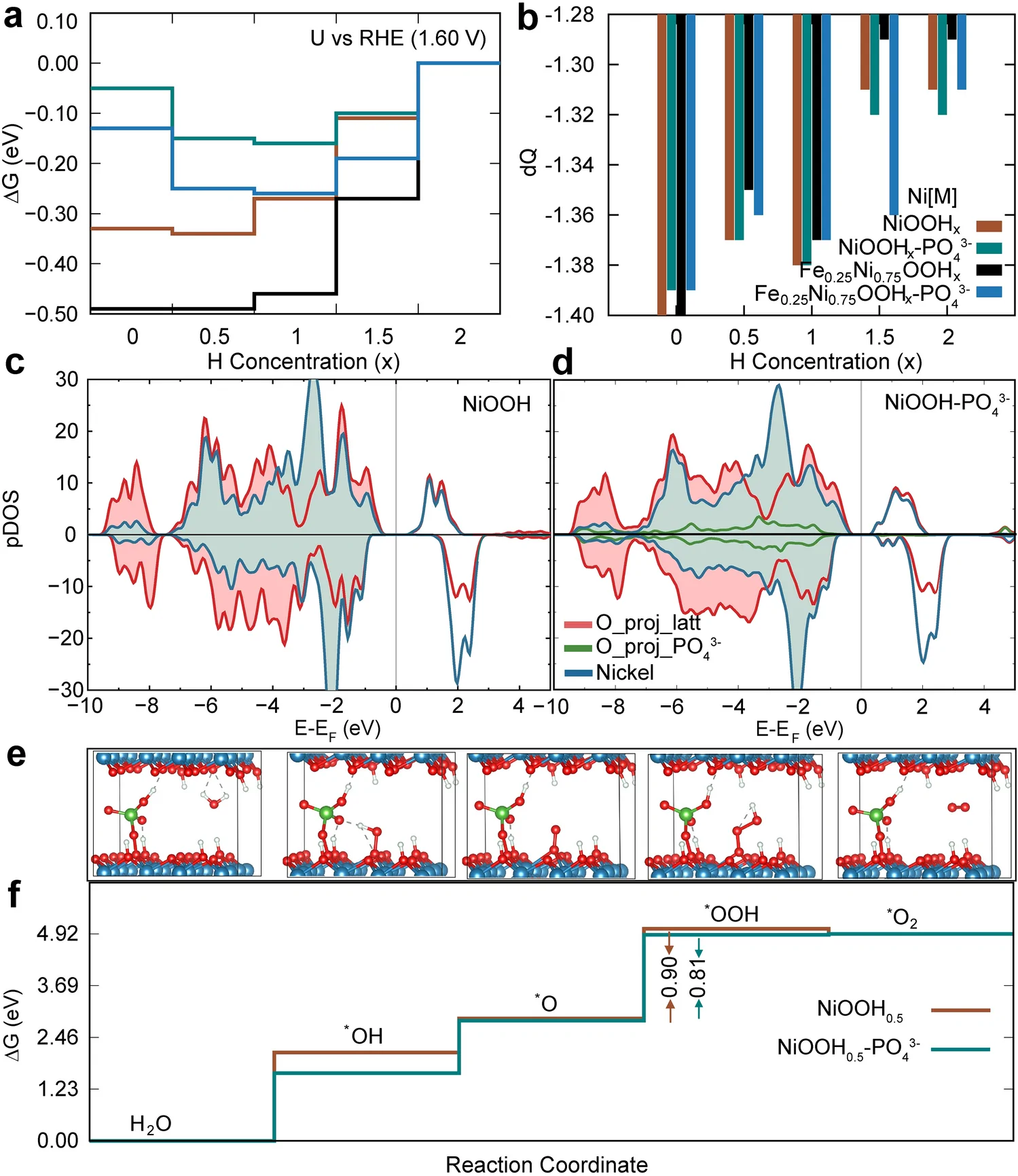
**a** Gibbs free energy of hydrogen adsorption (ΔG) as a function of hydrogen concentration (x) in NiOOH_x_ (black), NiOOH_x_-PO_4_^3^^–^ (red), Fe_0.25_Ni_0.75_O_2_H_x_ (blue), and Ni_0.75_Fe_0.25_OOH_x_-PO_4_^3^^–^ (green) at applied potentials of 1.60 V. **b** Bader charge analysis (ΔQ) of Ni atoms as a function of hydrogen concentration (x) in NiOOH_x_ (brown), NiOOH_x_-PO_4_^3^^–^ (green), Ni_0.75_Fe_0.25_OOH_x_ (black), and Ni_0.75_Fe_0.25_OOH_x_-PO_4_^3^^–^ (blue), resolved into medium-charge cluster. Projected density of states (pDOS) for **c** NiOOH and **d** NiOOH-PO_4_^3^^–^. **e** Side view of the optimized structural models used for the free-energy calculations and **f** the free-energy profiles for NiOOH_0.5_ with and without intercalated PO_4_^3^^–^

To elucidate the atomic mechanism by which PO_4_^3^^–^ enhances the OER activity of Ni and NiFe (oxy)hydroxide, we performed systematic density functional theory (DFT) calculations. Starting with NiOOH_x_ (x = 2), we have taken systematically deprotonated structural model compositions ranging from NiOOH_1.5_ to NiOOH_0.5_ and NiO_2,_ representing different states of charge during the OER process [60]. A (2 × 2) supercell was used to accommodate different hydrogen stoichiometries. We then introduced 25% Fe doping and modeled PO_4_^3^^–^ intercalation by adsorbing neutral PO_4_^3–^ onto the surface (see SI for computational details). The Gibbs free energy of hydrogen adsorption was computed using DFT + U and referenced to the computational reversible hydrogen electrode to express energies as a function of electrochemical potential. This approach allowed us to determine the equilibrium hydrogen content under applied potentials and assess the influence of PO_4_^3^^–^ on the protonation state of the lattice. As shown in Fig. 6a, at OER-relevant potentials (~ 1.6 V vs. RHE), each system exhibits distinct hydrogen stabilization behavior. Pristine NiOOH_x_ maintains a hydrogen coverage of x = 0.5, indicating a partially oxidized Ni state. With 25% Fe doping (Fe_0.25_Ni_0.75_OOH_x_), the stable H concentration increases to 1.0 at lower potentials and remains elevated (0.5–1.0) up to 1.6 V, demonstrating Fe’s role in promoting a more reduced Ni environment. When PO_4_^3^^–^ is intercalated, this effect is further enhanced. Notably, the system containing both Fe and PO_4_^3^^–^ consistently maintains a higher proton concentration (x = 1.0) across the operational potential window. This demonstrates a synergistic role of Fe and PO_4_^3^^–^, where Fe initially lowers the average Ni oxidation state, while PO_4_^3–^ acts as an effective redox buffer, preventing the Ni sites from becoming over-oxidized during the OER [61–64]. The synergic effect of Fe and PO_4_^3–^ allows fine control over the redox chemistry of Ni, corroborating the superior OER performance observed when both are present.

To quantify the electronic changes induced by the incorporation of PO_4_^3^^–^ and Fe, we conducted Bader charge analysis on nickel and the lattice oxygen as a function of the proton concentration, which is presented in Fig. 6b. The corresponding configurations are summarized in Figs. S35-S37. As H concentration increases, the Ni atoms exhibit progressively less positive Bader charges, consistent with a more reduced state, although the absolute values cannot be equated directly with formal oxidation states. Incorporation of Fe also leads to a relative reduction of Ni at fixed H concentration, whereas the introduction of PO_4_^3^^–^ counteracts this by partially reoxidizing Ni, particularly at intermediate proton concentrations. This suggests that PO_4_^3^^–^ acts as a redox buffer: while H and Fe drive the reduction of Ni, PO_4_^3^^–^ helps maintain Ni at an intermediate electron density regime, thereby preventing over-reduction or over-oxidation of Ni during cycling, which corroborates the energetics presented in Fig. 6a.

We further examined the electronic structure using hybrid functional (HSE) calculations. As shown in Fig. 6c, d, the pristine NiOOH model used exhibits a wide band gap (~ 0.85 eV), with the valence band maximum dominated by lattice O 2*p* states and the conduction band primarily composed of Ni 3*d* states. Upon PO_4_^3^^–^ incorporation, a change in the oxygen coordination environment shifts Ni 3*d* states closer to the Fermi level, further narrowing the bandgap (to ~ 0.56 eV) and broadening both valence and conduction band width, although the conduction band edge retains its Ni 3*d* and O 2*p* mixed character. Notably, the inclusion of Fe leads to an even narrower bandgap (to ~ 0.15 eV) (Fig. S38), which aligns with the experimentally observed reduction in R_ct_ (Ni_x_P_y_-A vs. NiFeP-A) shown in Fig. 2e. In summary, PO_4_^3^^–^ and Fe act synergistically to reduce the bandgap and enhance electronic conductivity by improving charge delocalization, favoring charge-transfer during OER.

Finally, using the computational hydrogen electrode (CHE) approach [65], we computed the free-energy profile for pristine NiOOH_0.5_, and Fe-doped NiOOH_0.5_, both with and without PO_4_^3^^–^ intercalation species, to identify the thermodynamic origin of the activity enhancement. Figure 6e and 6f exhibits the structures and OER profiles of pristine NiOOH_0.5_, with and without PO_4_^3^^–^. For the pristine surface, the *OOH formation step emerges as the potential-determining step (ΔG3 = 2.13 eV), corresponding to a theoretical overpotential (*η*) of 0.90 V. Upon PO_4_^3^^–^ intercalation, the overall reaction pathway remains identical, but ΔG3 decreases to 2.04 eV, reducing *η* to 0.81 V. Decomposition of the individual elementary steps shows that PO_4_^3^^–^ strongly stabilizes the *OH intermediate (ΔG1: 2.11 → 1.62 eV) and moderately stabilizes *OOH (ΔG3: 2.13 → 2.04 eV), while slightly destabilizing *O (ΔG2: 0.81 → 1.25 eV). This redistribution of adsorption energies effectively compresses the free-energy span among the key intermediates, bringing the overall reaction thermodynamics closer to the ideal four-electron pathway and rationalizing the lower *η* (Table S11). Consistent with our charge-density and Bader analyses, the PO_4_^3^^–^ moiety withdraws electron density from the Ni–O lattice, buffering Ni oxidation states while simultaneously stabilizing oxygenated adsorbates. This dual electronic and geometric effect promotes balanced intermediate binding and facilitates charge-transfer during the OER, in excellent agreement with the experimentally observed improvement in catalytic activity. Notably, this thermodynamic stabilization correlates with the electronic trends observed in the HSE-derived pDOS, where PO_4_^3^^–^ narrows the bandgap and broadens the valence band, enabling faster electron delocalization and more efficient *OOH formation.

Furthermore, the OER free-energy profiles for Fe-doped NiOOH_0.5_ with and without PO_4_^3^^–^ intercalation are provided in Fig. S39. Introducing 25% Fe substitution into the pristine host reduces *η* from 0.90 to 0.84 V, consistent with the well-established role of Fe as an activity-promoting dopant in Ni-based OER catalysts. Strikingly, the synergistic combination of Fe doping and PO_4_^3^^–^ intercalation yields a substantially more pronounced improvement: while the potential-determining step remains the *OOH formation, *η* is dramatically reduced to 0.52 V, representing a reduction of 0.38 V relative to the pristine system. This progressive decrease in overpotential across the series from pristine > PO_4_^3^^–^ intercalated > Fe-doped + PO_4_^3^^–^ intercalated unambiguously demonstrates that the two modifications act cooperatively rather than independently, collectively optimizing the adsorption energetics of key OER intermediates and pushing the catalytic performance closer to the thermodynamic ideal (Table S12).

To bridge the gap between the computational model and experimental reality, we additionally considered the fact that Fe doping in NiOOH inherently introduces O and OH vacancy defects under operating conditions [66, 67]. To capture this practical scenario, we explicitly modeled systems incorporating OH and O vacancies and computed their OER free-energy profiles, as presented in Fig. S40. The introduction of these vacancies breaks the local coordination symmetry around the active metal centers, generating undercoordinated sites that rebalance the binding energetics of *OH, *O, and *OOH intermediates (Table S12).

Overall, our DFT calculations reveal that PO_4_^3^^–^ intercalation, synergistically with Fe, serves as a redox buffer to prevent Ni over-oxidation, optimizes the electronic structure of Ni to enhance conductivity, and stabilizes key OER intermediates to lower the thermodynamic energy barrier (Fig. S41), collectively leading to enhanced catalytic activity.

## Conclusions

In this work, we prepared hollow NiFeP prisms composed of crystalline NiFeP domains surrounded by amorphous components containing Ni, Fe, O, P, and K through gas-phase phosphidation of pre-NiFe. During the OER process, the NiFeP dynamically reconstructed into ultrathin NiFe (oxy)hydroxide nanosheets, a transformation that has been directly captured in situ using IL-TEM and verified by ex situ TEM and XAFS analyses. The restructured catalyst exhibited robust stability and excellent OER activity under alkaline conditions, achieving low overpotentials of 225 mV to reach current densities of 10 mA cm^–^^2^, respectively, outperforming most reported OER catalysts. Beyond confirming the transformation, our systematic investigation disentangled the multiple essential roles of phosphorus: it accelerates the formation of the active NiFe (oxy)hydroxide phase, suppresses Fe dissolution, and modulates the electronic structure of Ni. The addition of PO_4_^3^^–^ ions into the electrolyte was found to significantly enhance the OER activity of the pre-Ni catalyst, further evidencing the beneficial role of phosphate species in promoting reaction kinetics. DFT calculations elucidated that PO_4_^3^^–^ and Fe act synergistically to prevent Ni over-oxidation, optimize electronic conductivity by narrowing the band gap, and stabilize key OER intermediates, thereby lowering the thermodynamic energy barrier of the reaction. Collectively, this work not only provides fundamental insights into phosphorus‑driven structural evolution but also demonstrates an effective strategy leveraging anionic species as active, synergistic components for developing cost‑effective and high‑performance electrocatalysts for alkaline water splitting and related energy conversion technologies.

## Supplementary Information

Below is the link to the electronic supplementary material.

Supplementary file1 (DOCX 14859 KB)

## References

[1] R. Ram, L. Xia, H. Benzidi, A. Guha, V. Golovanova et al., Water-hydroxide trapping in cobalt tungstate for proton exchange membrane water electrolysis. Science **384**(6702), 1373–1380 (2024). 10.1126/science.adk9849 38900890 10.1126/science.adk9849

[2] W. Shi, T. Shen, C. Xing, K. Sun, Q. Yan et al., Ultrastable supported oxygen evolution electrocatalyst formed by ripening-induced embedding. Science **387**(6735), 791–796 (2025). 10.1126/science.adr3149 39946454 10.1126/science.adr3149

[3] N. Shi, R. Ma, L. Lin, W. Xie, P. Liu et al., In-situ derived defective Ru particles anchored on Ru–Ni layered double hydroxides for enhanced alkaline hydrogen evolution. Small **20**(27), 2311076 (2024). 10.1002/smll.202311076 10.1002/smll.202311076 38279579

[4] Q. Hassan, S. Algburi, A.Z. Sameen, H.M. Salman, M. Jaszczur, A review of hybrid renewable energy systems: solar and wind-powered solutions: challenges, opportunities, and policy implications. Results Eng. **20**, 101621 (2023). 10.1016/j.rineng.2023.101621

[5] Q. Sha, S. Wang, L. Yan, Y. Feng, Z. Zhang et al., 10, 000-h-stable intermittent alkaline seawater electrolysis. Nature **639**(8054), 360–367 (2025). 10.1038/s41586-025-08610-1 40044863 10.1038/s41586-025-08610-1

[6] A. Odenweller, F. Ueckerdt, The green hydrogen ambition and implementation gap. Nat. Energy **10**(1), 110–123 (2025). 10.1038/s41560-024-01684-7

[7] N. Shi, W. Xue, P. Liu, C. Zeng, S. Yue et al., Atomically dispersed Ru on NiFeV layered triple hydroxides for enhanced water oxidation. Adv. Funct. Mater. **35**(24), 2421740 (2025). 10.1002/adfm.202421740

[8] M.T.M. Koper, Thermodynamic theory of multi-electron transfer reactions: implications for electrocatalysis. J. Electroanal. Chem. **660**(2), 254–260 (2011). 10.1016/j.jelechem.2010.10.004

[9] J. Zhou, P. Li, X. Xia, Y. Zhao, Z. Hu et al., Precisely tailoring the d-band center of nickel sulfide for boosting overall water splitting. Appl. Catal. B Environ. Energy **359**, 124461 (2024). 10.1016/j.apcatb.2024.124461

[10] M.-I. Jamesh, H. Tong, S.P. Santoso, W. Niu, J.-J. Kai et al., Recent advances in developing nanoscale electro-/photocatalysts for hydrogen production: modification strategies, charge-carrier characterizations, and applications. Nanoscale **16**(39), 18213–18250 (2024). 10.1039/D4NR01178C 39291727 10.1039/d4nr01178c

[11] M.-I. Jamesh, D. Hu, J. Wang, F. Naz, J. Feng et al., Recent advances in noble metal-free electrocatalysts to achieve efficient alkaline water splitting. J. Mater. Chem. A. **12**(20), 11771–11820 (2024). 10.1039/d3ta07418h

[12] M.-I. Jamesh, A. Akila, D. Sudha, K.G. Priya, V. Sivaprakash et al., Fabrication of earth-abundant electrocatalysts based on green-chemistry approaches to achieve efficient alkaline water splitting: a review. Sustainability **14**(24), 1–48 (2022). 10.3390/su142416359

[13] J. Wang, M.-I. Jamesh, Q. Gao, B. Han, R. Sun et al., Semimetallic hydroxide materials for electrochemical water oxidation. Sci. China Mater. **67**(5), 1551–1558 (2024). 10.1007/s40843-023-2802-8

[14] J. Nie, J. Shi, L. Li, M.-Y. Xie, Z.-Y. Ouyang et al., Anion-mediated rapid and direct synthesis of FeNiOOH for robust water oxidation. Adv. Funct. Mater. **35**(5), 2414493 (2025). 10.1002/adfm.202414493

[15] M.-Y. Xie, J.-R. Huang, H. Wan, J. Nie, M.-H. Xian et al., Quasi-*in situ* redeposition-enabled stabilization of NiFe-based (oxy)hydroxides under high OER current density. Appl. Phys. Lett. **126**(15), 153901 (2025). 10.1063/5.0261319

[16] J.-R. Huang, M.-Y. Xie, M.-H. Xian, Y. Luo, J.-H. Nie et al., Redefining Fe-doping effects: Solution-phase redox cycling dominates pre-lattice Ni^3^^**+**^ formation and 2/1.5D nanostructure for enhanced OER. Appl. Phys. Lett. **127**(10), 103905 (2025). 10.1063/5.0287885

[17] Z.-P. Wu, S. Zuo, Z. Pei, J. Zhang, L. Zheng et al., Operando unveiling the activity origin via preferential structural evolution in Ni-Fe (oxy)phosphides for efficient oxygen evolution. Sci. Adv. **11**(10), 5370 (2025). 10.1126/sciadv.adu5370 40053602 10.1126/sciadv.adu5370 PMC11887844

[18] N. Zhang, Y. Hu, Z. Zhang, C. Wu, J. Zhu et al., Crystallinity-dependent structural evolution of CoS_2_ catalysts for enhanced oxygen evolution reaction. Nat. Commun. **16**(1), 9306 (2025). 10.1038/s41467-025-64346-6 41120311 10.1038/s41467-025-64346-6 PMC12540841

[19] H.Y. Lin, W.J. Li, M.Y. Lin, H.G. Xu, S.R. Fang et al., Leaching-induced Ti trapping stabilizes amorphous IrO_x_ for proton exchange membrane water electrolysis. Angew. Chem. Int. Ed. Engl. **64**(26), e202504212 (2025). 10.1002/anie.202504212 40257792 10.1002/anie.202504212

[20] L. Tan, J. Wang, S. Zhou, H. Zhu, J. Guo et al., NiFe phosphides coupled on Ti_3_C_2_T_x_ MXene nanosheets for high-efficiency oxygen evolution reaction in alkaline medium. J. Colloid Interface Sci. **689**, 137263 (2025). 10.1016/j.jcis.2025.137263 40080983 10.1016/j.jcis.2025.137263

[21] Y. Lee, W. Jeong, Y.J. Hwang, B. An, H. Lee et al., Basics, developments, and strategies of transition metal phosphides toward electrocatalytic water splitting: beyond noble metal catalysts. J. Mater. Chem. A. **12**(42), 28574–28594 (2024). 10.1039/d4ta04455j

[22] S. Sun, X. Zhou, B. Cong, W. Hong, G. Chen, Tailoring the d-band centers endows (Ni_*x*_Fe_1–__*x*_)_2_P nanosheets with efficient oxygen evolution catalysis. ACS Catal. **10**(16), 9086–9097 (2020). 10.1021/acscatal.0c01273

[23] Q. Zhou, C. Xu, J. Hou, W. Ma, T. Jian et al., Duplex interpenetrating-phase FeNiZn and FeNi_3_ heterostructure with low-Gibbs free energy interface coupling for highly efficient overall water splitting. Nano-Micro Lett. **15**(1), 95 (2023). 10.1007/s40820-023-01066-w 10.1007/s40820-023-01066-w PMC10086094 37037951

[24] M. Cai, Q. Zhu, X. Wang, Z. Shao, L. Yao et al., Formation and stabilization of NiOOH by introducing α-FeOOH in LDH: composite electrocatalyst for oxygen evolution and urea oxidation reactions. Adv. Mater. **35**(7), 2209338 (2023). 10.1002/adma.202209338 10.1002/adma.202209338 36401826

[25] X. Wang, I.A. Cechanaviciute, L. Banko, S. Pokharel, T. Quast et al., From quinary co–Cu–Mo–Pd–Re materials libraries to gas diffusion electrodes for alkaline hydrogen evolution. Adv. Funct. Mater. **34**(33), 2400180 (2024). 10.1002/adfm.202400180

[26] F. Hu, S. Zhu, S. Chen, Y. Li, L. Ma et al., Amorphous metallic NiFeP: a conductive bulk material achieving high activity for oxygen evolution reaction in both alkaline and acidic media. Adv. Mater. **29**(32), 1606570 (2017). 10.1002/adma.201606570 10.1002/adma.201606570 28639333

[27] J. Yu, G. Cheng, W. Luo, Hierarchical NiFeP microflowers directly grown on Ni foam for efficient electrocatalytic oxygen evolution. J. Mater. Chem. A **5**(22), 11229–11235 (2017). 10.1039/C7TA02968C

[28] J. Wang, F. Ciucci, In-situ synthesis of bimetallic phosphide with carbon tubes as an active electrocatalyst for oxygen evolution reaction. Appl. Catal. B Environ. **254**, 292–299 (2019). 10.1016/j.apcatb.2019.05.009

[29] B. Yang, D. Bin, A.G. Tamirat, Y. Liu, L. Liu et al., Bamboo-like nitrogen-doped carbon nanotubes encapsulated with NiFeP nanoparticles and their efficient catalysis in the oxygen evolution reaction. Electrochim. Acta **331**, 135360 (2020). 10.1016/j.electacta.2019.135360

[30] J. Chen, Q. Long, K. Xiao, T. Ouyang, N. Li et al., Vertically-interlaced NiFeP/MXene electrocatalyst with tunable electronic structure for high-efficiency oxygen evolution reaction. Sci. Bull. **66**(11), 1063–1072 (2021). 10.1016/j.scib.2021.02.033 10.1016/j.scib.2021.02.033 36654340

[31] E. Vijayakumar, S. Ramakrishnan, C. Sathiskumar, D.J. Yoo, J. Balamurugan et al., MOF-derived CoP-nitrogen-doped carbon@NiFeP nanoflakes as an efficient and durable electrocatalyst with multiple catalytically active sites for OER, HER, ORR and rechargeable zinc-air batteries. Chem. Eng. J. **428**, 131115 (2022). 10.1016/j.cej.2021.131115

[32] W. Chen, H. Wang, Y. Li, Y. Liu, J. Sun et al., *In situ* electrochemical oxidation tuning of transition metal disulfides to oxides for enhanced water oxidation. ACS Cent. Sci. **1**(5), 244–251 (2015). 10.1021/acscentsci.5b00227 27162978 10.1021/acscentsci.5b00227 PMC4827502

[33] L.-A. Stern, L. Feng, F. Song, X. Hu, Ni_2_P as a Janus catalyst for water splitting: the oxygen evolution activity of Ni_2_P nanoparticles. Energy Environ. Sci. **8**(8), 2347–2351 (2015). 10.1039/C5EE01155H

[34] X. Xu, F. Song, X. Hu, A nickel iron diselenide-derived efficient oxygen-evolution catalyst. Nat. Commun. **7**, 12324 (2016). 10.1038/ncomms12324 27503136 10.1038/ncomms12324 PMC4980491

[35] K. Fan, H. Zou, Y. Lu, H. Chen, F. Li et al., Direct observation of structural evolution of metal chalcogenide in electrocatalytic water oxidation. ACS Nano **12**(12), 12369–12379 (2018). 10.1021/acsnano.8b06312 30508382 10.1021/acsnano.8b06312

[36] P. Yan, Q. Liu, H. Zhang, L. Qiu, H.B. Wu et al., Deeply reconstructed hierarchical and defective NiOOH/FeOOH nanoboxes with accelerated kinetics for the oxygen evolution reaction. J. Mater. Chem. A **9**(28), 15586–15594 (2021). 10.1039/D1TA03362J

[37] Y. Hu, Y. Zheng, J. Jin, Y. Wang, Y. Peng et al., Understanding the sulphur-oxygen exchange process of metal sulphides prior to oxygen evolution reaction. Nat. Commun. **14**(1), 1949 (2023). 10.1038/s41467-023-37751-y 37029185 10.1038/s41467-023-37751-y PMC10082196

[38] U. De Silva, J. See, W.P.R. Liyanage, J. Masud, J. Wu et al., Understanding the structural evolution of a nickel chalcogenide electrocatalyst surface for water oxidation. Energy Fuels **35**(5), 4387–4403 (2021). 10.1021/acs.energyfuels.0c04089

[39] T. Chen, B. Li, K. Song, C. Wang, J. Ding et al., Defect-activated surface reconstruction: mechanism for triggering the oxygen evolution reaction activity of NiFe phosphide. J. Mater. Chem. A **10**(42), 22750–22759 (2022). 10.1039/D2TA04879E

[40] F. Zhao, X. Mao, X. Zheng, H. Liu, L. Zhu et al., Roles of the self-reconstruction layer in the catalytic stability of a NiFeP catalyst during the oxygen evolution reaction. J. Mater. Chem. A **11**(1), 276–286 (2023). 10.1039/D2TA06514B

[41] J. Liu, X. Liu, H. Shi, J. Luo, L. Wang et al., Breaking the scaling relations of oxygen evolution reaction on amorphous NiFeP nanostructures with enhanced activity for overall seawater splitting. Appl. Catal. B Environ. **302**, 120862 (2022). 10.1016/j.apcatb.2021.120862

[42] A. Nandy, T. Ghosh, R. Kumar, D. Bhattacharyya, D. Senapati, Electronic properties modulation of NiFe-based nanoalloy by introducing Cu and P for faster oxygen evolution reaction kinetics. ACS Appl. Energy Mater. **7**(3), 1109–1119 (2024). 10.1021/acsaem.3c02617

[43] C. Qiu, F. Maroun, M. Bouvier, I. Pacheco, P. Allongue et al., Operando surface X-ray diffraction studies of epitaxial Co_3_O_4_ and CoOOH thin films during oxygen evolution: pH dependence. ChemCatChem **16**(23), e202400988 (2024). 10.1002/cctc.202400988

[44] E. Ronge, J. Ohms, V. Roddatis, T. Jones, F. Sulzmann et al., Operation of calcium-birnessite water-oxidation anodes: interactions of the catalyst with phosphate buffer anions. Sustainable Energy Fuels **5**(21), 5535–5547 (2021). 10.1039/D1SE01076J

[45] Z.-X. Qian, G.-H. Liang, L.-F. Shen, G. Zhang, S. Zheng et al., Phase engineering facilitates O-O coupling *via* lattice oxygen mechanism for enhanced oxygen evolution on nickel-iron phosphide. J. Am. Chem. Soc. **147**(1), 1334–1343 (2025). 10.1021/jacs.4c15847 39721054 10.1021/jacs.4c15847

[46] L. Yu, J.F. Yang, B.Y. Guan, Y. Lu, X.W. Lou, Hierarchical hollow nanoprisms based on ultrathin Ni-Fe layered double hydroxide nanosheets with enhanced electrocatalytic activity towards oxygen evolution. Angew. Chem. Int. Ed. **57**(1), 172–176 (2018). 10.1002/anie.201710877 10.1002/anie.201710877 29178355

[47] H. Kim, Y. Chae, D. Lee, M. Kim, J. Huh et al., Palladium nanoparticle catalyzed conversion of iron nanoparticles into diameter- and length-controlled Fe_2_P nanorods. Angew. Chem. Int. Ed. **49**(33), 5712–5716 (2010). 10.1002/anie.201001822 10.1002/anie.201001822 20602386

[48] D.-H. Ha, L.M. Moreau, C.R. Bealing, H. Zhang, R.G. Hennig et al., The structural evolution and diffusion during the chemical transformation from cobalt to cobalt phosphidenanoparticles. J. Mater. Chem. **21**(31), 11498–11510 (2011). 10.1039/C1JM10337G

[49] J. Ryu, N. Jung, J.H. Jang, H.-J. Kim, S.J. Yoo, *In situ* transformation of hydrogen-evolving CoP nanoparticles: toward efficient oxygen evolution catalysts bearing dispersed morphologies with co-oxo/hydroxo molecular units. ACS Catal. **5**(7), 4066–4074 (2015). 10.1021/acscatal.5b00349

[50] P.E.R. Blanchard, A.P. Grosvenor, R.G. Cavell, A. Mar, X-ray photoelectron and absorption spectroscopy of metal-rich phosphides M_2_P and M_3_P (M = Cr−Ni). Chem. Mater. **20**(22), 7081–7088 (2008). 10.1021/cm802123a

[51] H.W. Nesbitt, D. Legrand, G.M. Bancroft, Interpretation of Ni2*p* XPS spectra of Ni conductors and Ni insulators. Phys. Chem. Miner. **27**(5), 357–366 (2000). 10.1007/s002690050265

[52] N.S. McIntyre, D.G. Zetaruk, X-ray photoelectron spectroscopic studies of iron oxides. Anal. Chem. **49**(11), 1521–1529 (1977). 10.1021/ac50019a016

[53] J. Min, J. Liu, M. Lei, W. Wang, Y. Lu et al., Self-assembly of parallelly aligned NiO hierarchical nanostructures with ultrathin nanosheet subunits for electrochemical supercapacitor applications. ACS Appl. Mater. Interfaces **8**(1), 780–791 (2016). 10.1021/acsami.5b09997 26674109 10.1021/acsami.5b09997

[54] S. Li, L. Wang, H. Su, A.N. Hong, Y. Wang et al., Electron redistributed S-doped nickel iron phosphides derived from one-step phosphatization of MOFs for significantly boosting electrochemical water splitting. Adv. Funct. Mater. **32**(23), 2200733 (2022). 10.1002/adfm.202200733

[55] H. Yu, M.E. Sweers, L. Osmieri, J.H. Park, A.J. Kropf et al., Synergy between Ni and Fe in NiFe aerogel oxygen evolution reaction catalyst: *in situ*^57^Fe Mössbauer and X-ray absorption spectroscopy studies. EES Catalysis **3**(6), 1229–1245 (2025). 10.1039/D5EY00127G

[56] P. Acharya, J. Hong, R. Manso, A.S. Hoffman, L. Kekedy-Nagy et al., Temporal Ni K-edge X-ray absorption spectroscopy study reveals the kinetics of the Ni redox behavior of the iron-nickel oxide bimetallic OER catalyst. J. Phys. Chem. C **127**(25), 11891–11901 (2023). 10.1021/acs.jpcc.3c03480

[57] D. Wang, Y. Wang, Z. Fu, Y. Xu, L.-X. Yang et al., Cobalt–nickel phosphate composites for the all-phosphate asymmetric supercapacitor and oxygen evolution reaction. ACS Appl. Mater. Interfaces **13**(29), 34507–34517 (2021). 10.1021/acsami.1c04614 34255472 10.1021/acsami.1c04614

[58] T. Li, R. Cai, X. Nie, J. Tjong, Porous Ni_3_(PO_4_)_2_ thin film as a binder-free and low-cost anode of a high-capacity lithium-ion battery. J. Electroanal. Chem. **835**, 81–85 (2019). 10.1016/j.jelechem.2019.01.021

[59] H. Tan, J. Verbeeck, A. Abakumov, G. Van Tendeloo, Oxidation state and chemical shift investigation in transition metal oxides by EELS. Ultramicroscopy **116**, 24–33 (2012). 10.1016/j.ultramic.2012.03.002

[60] Z.K. Goldsmith, A.K. Harshan, J.B. Gerken, M. Vörös, G. Galli et al., Characterization of NiFe oxyhydroxide electrocatalysts by integrated electronic structure calculations and spectroelectrochemistry. Proc. Natl. Acad. Sci. U.S.A. **114**(12), 3050–3055 (2017). 10.1073/pnas.1702081114 28265083 10.1073/pnas.1702081114 PMC5373414

[61] G. Huang, T. Yuan, B. Li, C. Peng, L. Wang et al., Unraveling oxyanion effects on oxygen evolution electrocatalysis of nickel hydr(oxy)oxides: the critical role of Fe impurities. Nano Lett. **25**(14), 5803–5811 (2025). 10.1021/acs.nanolett.5c00462 40148233 10.1021/acs.nanolett.5c00462

[62] Q. Zhang, W. Xiao, H.C. Fu, X.L. Li, J.L. Lei et al., Unraveling the mechanism of self-repair of NiFe-based electrocatalysts by dynamic exchange of iron during the oxygen evolution reaction. ACS Catal. **13**(22), 14975–14986 (2023). 10.1021/acscatal.3c03804

[63] Z. Zhang, Y. Luo, K. Wang, Q. Yu, X. Kang et al., Dynamically activating Ni-based catalysts with self-anchored mononuclear Fe for efficient water oxidation. J. Mater. Chem. A **11**(19), 10228–10238 (2023). 10.1039/D3TA00866E

[64] M.W. Louie, A.T. Bell, An investigation of thin-film Ni-Fe oxide catalysts for the electrochemical evolution of oxygen. J. Am. Chem. Soc. **135**(33), 12329–12337 (2013). 10.1021/ja405351s 23859025 10.1021/ja405351s

[65] J.K. Nørskov, J. Rossmeisl, A. Logadottir, L. Lindqvist, J.R. Kitchin et al., Origin of the overpotential for oxygen reduction at a fuel-cell cathode. J. Phys. Chem. B **108**(46), 17886–17892 (2004). 10.1021/jp047349j 39682080 10.1021/jp047349j

[66] S. Liu, H. Zhang, E. Hu, T. Zhu, C. Zhou et al., Boosting oxygen evolution activity of NiFe-LDH using oxygen vacancies and morphological engineering. J. Mater. Chem. A. **9**(41), 23697–23702 (2021). 10.1039/D1TA06263H

[67] Y. Chen, J. Guo, B. Chen, Oxygen vacancy-induced crystal-amorphous interface in NiFe LDH catalyst for enhanced OER performance. Surf. Interfaces. **69**, 106829 (2025). 10.1016/j.surfin.2025.106829

