# Supplementary material for: Unlocking the Synergistic Promoter Role of Phosphorus in Evolving NiFe Phosphides for Enhanced Water Oxidation
Source: Nanomicro Lett. 2026 Jun 11;18:400. doi: 10.1007/s40820-026-02238-0 (PMC13260567; doi:10.1007/s40820-026-02238-0)
Supplement: Supplementary file 1 — Supplementary file1 (DOCX 14859 KB) [file 40820_2026_2238_MOESM1_ESM.docx]

Supporting Information for

**Unlocking the Synergistic Promoter Role of Phosphorus in Evolving NiFe Phosphides for Enhanced Water Oxidation**

Ningning Shi ^1, 2, 3, #^, Mingcheng Gao ^1, 2, #^, M. Maneesha ^4^, C. S. Praveen ^4,^ *, Panpan Liu ^1, 2^, Shengnan Yue ^1, 2^, Wangjing Xie ^1, 2^, Dechao Chen ^1^, Yu Tang ^1^, Yuanqing Wang ^5,^ *, Hua Fan ^1^, and Xing Huang ^1, 2, 3,^ *

^1^ State Key Laboratory of Green and Efficient Development of Phosphorus Resources, College of Chemistry, Fuzhou University, 350108 Fuzhou, P. R. China

^2^ Electron Microscopy Center, Qingyuan Innovation Laboratory, 362100 Quanzhou, P. R. China

^3^ School of Chemistry, Chemical Engineering and Materials, Jining University, 273155 Qufu, P. R. China

^4^ International School of Photonics, Cochin University of Science and Technology, 682022 Cochin, India

^5^ Materials Genome Institute, Shanghai University, 200444 Shanghai, P. R. China

^#^ Ningning Shi and Mingcheng Gao contributed equally to this work.

*Corresponding authors. E-mail: [praveen@gmail.com](mailto:praveen@gmail.com) (C. S. Praveen); [yuanqingwang@shu.edu.cn](mailto:yuanqingwang@shu.edu.cn) (Yuanqing Wang); [xinghuang@fzu.edu.cn](mailto:xinghuang@fzu.edu.cn) (Xing Huang)

**S1 Supplementary Experimental Section**

**S1.1 Electrochemical measurements**

The electrochemical measurements were performed in a single-chamber three-electrode reactor with a CHI660e electrochemical analyzer (CH Instruments, Inc., Shanghai). For high current density durability tests at 500 mAcm⁻^2^, we used the DH7000D analyzer (Donghua Analytical Instruments Co., Ltd., Jiangsu, capable of ±1 A). The catalysts coated on Carbon Fiber Paper (CFP) were used as the working electrode, the Hg/HgO reference electrode was used as the reference electrode, and a graphite rod was used as the counter electrode. The Hg/HgO electrode converted to a reverse hydrogen electrode (RHE) has been calibrated (see Fig. S11). In this study, *E*(RHE) = *E*(Hg/HgO) + 0.9153 V. Each catalyst (2 mg) was dispersed with Nafion (20 μL, 5 wt% in a mixture of lower aliphatic alcohol and water in alcohol (180 μL). The mixture was sonicated for 30 min to form a uniform catalyst ink. 50 µL of the dispersion of homogeneous ink was loaded onto a 2×0.5 cm^2^ carbon fiber paper (CFP) electrode with an effective area of 1×0.5 cm^2^ and allowed to dry naturally at room temperature. The loading amount of the electrocatalysts on CFP was calculated to be 1 mg cm^−2^.

Cyclic voltammetry with a scan rate of 5 mV s^−1^ was conducted for 40 cycles to activate the working electrode. The linear sweep voltammetry measurements were conducted with a scan rate of 5 mV s^−1^ in 1.0 M KOH solution. Before the experiment, the electrolyte solution was bubbled with Ar (99.999%) for 30 minutes to ensure the formation of an inert gas-saturated solution. The electrochemical impedance spectroscopy (EIS) was carried out at 1.56 V *vs.* RHE in a frequency range from 100 kHz to 10 mHz with an amplitude of 5 mV. All polarization curves were recorded with 100% iR correction. Unless otherwise explicitly stated, all current densities mentioned herein refer to values normalized per geometric area. Tafel plot of the overpotential vs. log (j) was calculated by fitting the linear portion at low overpotential to the Tafel equation (η = a + b log j, where η is the overpotential, j is the anodic current density, and b is the Tafel slope). The electrochemical surface area (ECSA) of the electrode was evaluated from their double-layer electrochemical capacitances (C_dl_) in a non-faradic region by a simple cyclic voltammetry method. By plotting the capacitive currents (j_anodic_ – j_cathodic_) / 2 vs. scan rate, the C_dl_ at the solid–liquid interface of materials can be estimated from the slope. The ECSA of a catalyst is proportional to its C_dl_: ECSA = $\frac{C_{\mathrm{dl}}}{C_{s}}$, where C_s_ is assumed to be 0.040 mF cm^-2^ [S1]. The stability tests were also performed using a typical three-electrode system. The specific activity values were calculated from the normalized ECSA values. Mass activity values were calculated from the electrocatalyst loading m (1 mg cm^-2^) and the measured current density j at η = 300 mV according to the equation: Mass activity =$\frac{j}{m}$. The TOF value was calculated by the equation: TOF = $\frac{I}{\alpha Fn}$, where *I* is the current at *η* = 300 mV, *F* is Faraday’s constant, *α* is the number of electrons consumed to form one O_2_ molecule from water (4 electrons for OER), and *n* is the number of active sites (See details from TOF calculation part).

**S1.2 DFT calculations**

All DFT calculations were performed using the Quantum ESPRESSO package [S2]. A monolayer Ni(OH)_2_ was constructed along the [0001] direction, with the interplanar spacing fixed at 7.6 Å to match the experimental observations. The geometries were taken from Sharon Hammes-Schiffer et.al. [S3], and the atomic positions were relaxed at the PBE+U level with ortho-atomic projections and spin polarization, wherein the PBE exchange-correlation functional was augmented with Hubbard U corrections (U = 5.5 eV for Ni 3*d* and U = 5.3 eV for Fe 3*d*) to capture correlation of the Ni and Fe 3d electrons. To account for the core electrons, ultrasoft (For Ni, H, and P) and PAW pseudopotentials, (for Fe and O) taken from the SSSP Library were used [S4]. We employed a kinetic energy cutoff of 60 Ry for the wavefunctions and 480 Ry for the charge density. Brillouin zone integrations employed a Γ-centered (2 × 2 × 2) **k**-point mesh, with Fermi-Dirac smearing of 0.005 Ry. After full structural optimization, the electronic density of states (DOS) was calculated using the screened hybrid HSE functional, incorporating 15% exact exchange. For the HSE calculations, the geometries optimized at the PBE+U level were kept fixed, and optimized norm-conserving Vanderbilt (ONCV) pseudopotentials [S5] were used for all elements to ensure reliable treatment of the nonlocal Fock exchange. The plane-wave cutoff energies were increased to 80 Ry for wavefunctions, and reduced to 320 Ry for charge density, and 160 Ry for the exact exchange, with the same (2 × 2 × 2) **k**-point mesh. Marzari-Vanderbilt smearing (width 0.001 Ry) was employed, and the Gygi-Baldereschi method was used for the treatment of exact exchange divergence. The Gibbs free energy of the OER intermediates was calculated using the computational hydrogen electrode model [S6] nd is given by *G* = *E*_tot-sys_ + *E*_ZPE_ -*TS*, where *E*_tot-sys_ is the DFT energy, *E*_ZPE_ is the zero point energy, *T* is the room temperature, and *S* is the entropy, which is taken as non-zero only for the aqueous and gas phase species. The reaction free energy (∆*G*) of each intermediate step was found using

$\Delta G_{1}=G_{OH}-E_{slab}-G_{H_{2}O}\left( l \right)+0.5\times G_{H_{2}}(g)$ (S1)

$\Delta G_{2}=G_{O}-G_{OH}+0.5 \times G_{H_{2}}\left( g \right)$ (S2)

$\Delta G_{3}=G_{OOH}-G_{O}-G_{H_{2}O}\left( l \right)+0.5 \times G_{H_{2}}\left( g \right)$ (S3)

$\Delta G_{4}=G_{O_{2}}\left( g \right)+E_{slab}+0.5 \times G_{H_{2}}\left( g \right)-G_{OOH}$ (S4)

and the theoretical overpotential is calculated as

η = max[∆*G*_1,_ ∆*G*_2,_ ∆*G*_3,_ ∆*G*_4_ ] $-$ U_eq_, where U_eq_= 1.23 eV [S6].

Due to the limitations of DFT in accurately predicting the high-spin ground state of the O_2_ molecule, its free energy (G_O2_(g)) is estimated using the relation

$G_{O_{2}}\left( g \right)=2 G_{H_{2}O}\left( l \right)-2G_{H_{2}}\left( g \right)+4.92 eV$ (S5)

where 4.92 eV is the total standard free energy change for the water splitting reaction [S7].

The free energy of gaseous phase H_2_O at 0.035 bar was taken as the reference state since at this pressure gas phase H_2_O is in equilibrium with liquid water at room temperature [S8].

**Table S1** Zero-point energy and entropy at room temperature

|  | ZPE (eV) | TS (eV) |
| --- | --- | --- |
| *OH | 0.386 | 0.07 |
| *O | 0.084 | 0.05 |
| *OOH | 0.457 | 0.16 |
| H_2_O | 0.56 | 0.583 |
| H_2_ | 0.27 | 0.404 |

The entropy and zero-point energy values listed in Table 1 were sourced as follows: gas-phase values were taken from the NIST Computational Chemistry Comparison and Benchmark Database [S9] while values for adsorbed species were taken from a previous study [S10].

**Supplementary Figures and Tables**


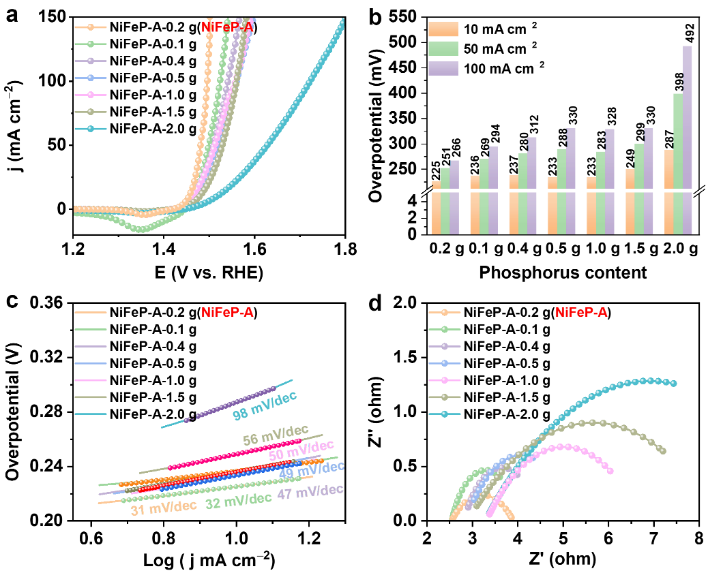


**Fig. S1** Catalytic performance evaluation of catalysts obtained using different phosphorus precursor amounts. **a** Polarization curves (backward-scan profiles) at a scan rate of 5 mV s^–1^. **b** Overpotentials at current densities of 10, 50, and 100 mA cm^−2^. **c** Tafel plots. **d** Nyquist plots.

**Discussion on Fig. S1:** To quantitatively investigate the correlation between phosphorus content and OER performance, we prepared a series of NiFeP catalysts with varying phosphorus contents by adjusting the amount of P precursor (NaH_2_PO_2_) from 0.1g to 2.0 g during the phosphidation step. After identical electrochemical activation (CV + LSV), the OER performance was measured and the residual phosphate content in each activated catalyst was determined by STEM-EDX.

As shown in Fig. S1, we observed a clear correlation between use of P precursor amount and OER performance, with the catalyst using 0.2 g of NaH_2_PO_2_ precursor exhibiting the best performance. Subsequent EDX analysis revealed that the residual phosphorus content in the activated catalysts was roughly proportional to the amount of P precursor used (Table S1). This result suggests that a moderate amount of intercalated PO_4_^3^⁻ is sufficient to achieve the desired redox‑buffering effect (see our DFT calculations) without blocking active sites.


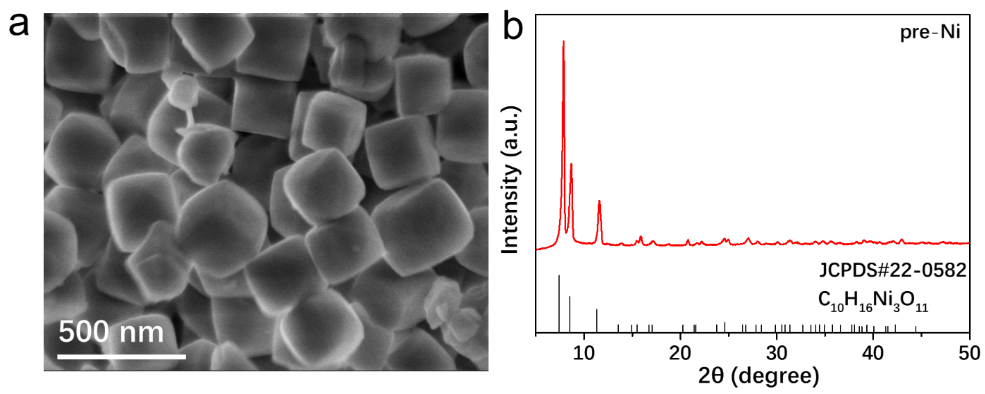


**Fig. S2 a** SEM image and **b** XRD patterns of pre-Ni.


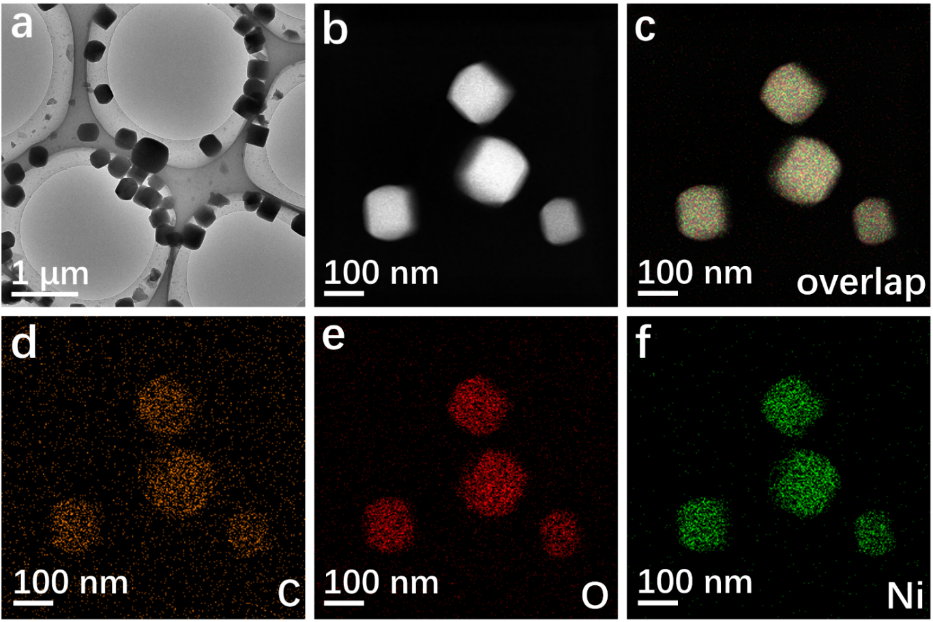


**Fig. S3** **a** TEM image, **b** HAADF-STEM image, and **c-f** corresponding elemental mappings of the pre-Ni.


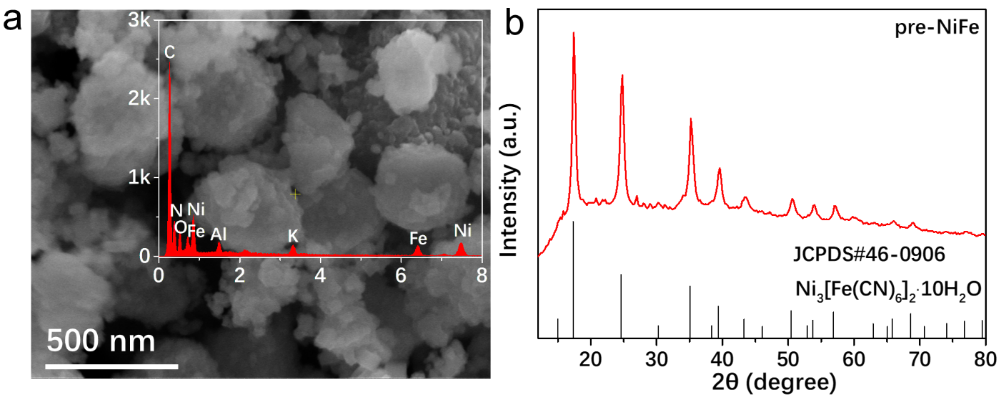


**Fig. S4 a** SEM image and **b** XRD patterns of pre-NiFe. Inset is SEM-EDX spectrum of pre-NiFe.


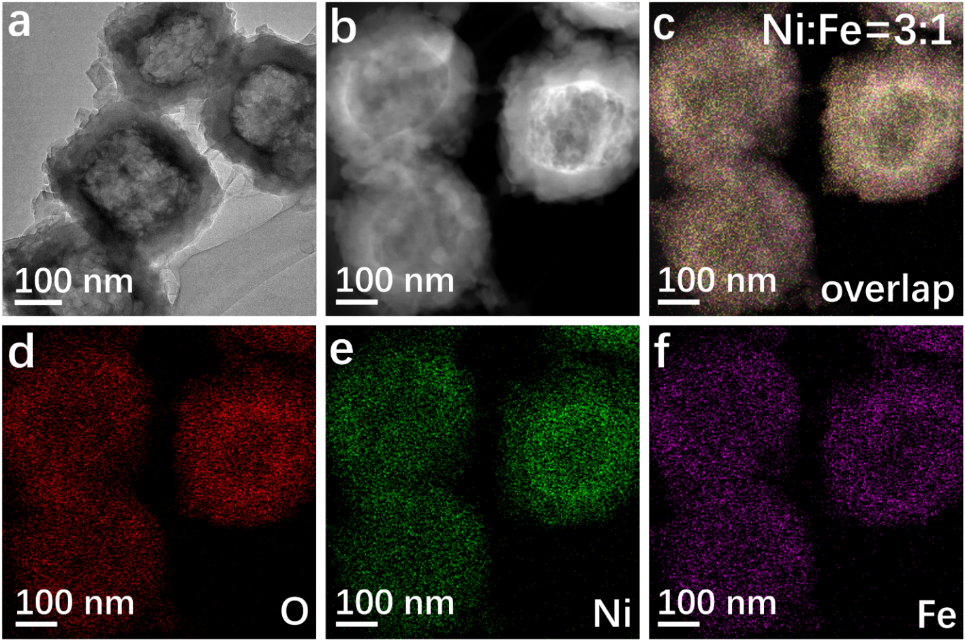


**Fig. S5** **a** TEM image, **b** HAADF-STEM image, and **c-f** corresponding elemental mappings of the pre-NiFe.


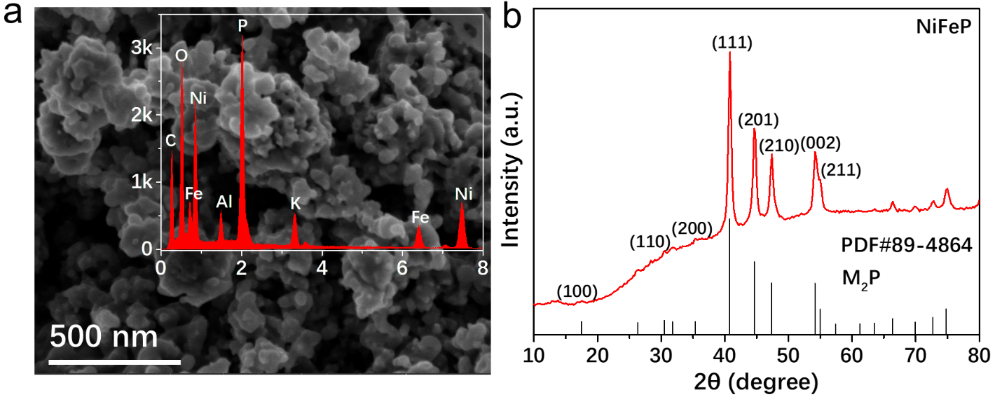


**Fig. S6 a** SEM image and **b** XRD patterns of NiFeP. Inset is SEM-EDX spectrum of NiFeP.

**
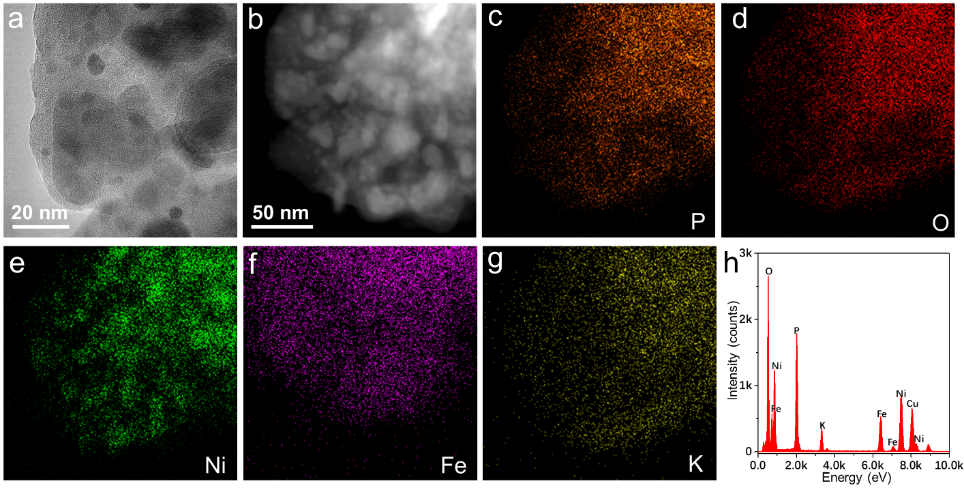
**

**Fig. S7 a** TEM image and **b** HAADF-STEM image of coating layers of as-prepared NiFeP. **c-g** Corresponding elemental maps and EDX line profiling spectra of NiFeP.


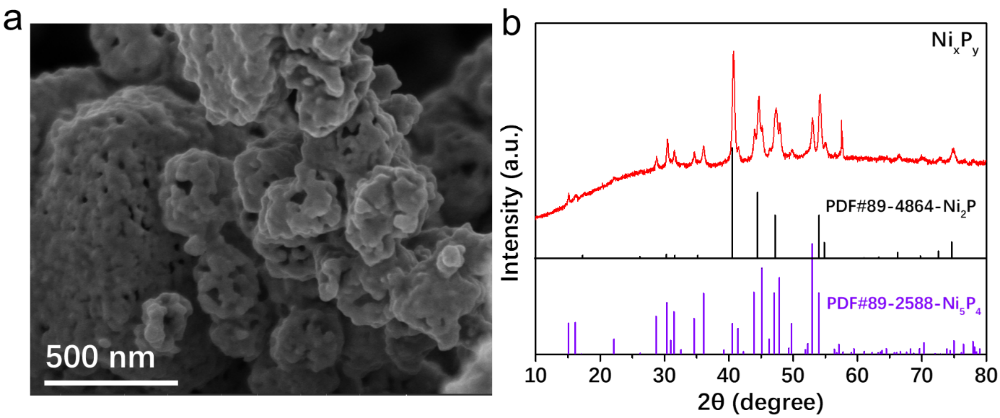


**Fig. S8 a** SEM image and **b** XRD patterns of Ni_x_P_y_.


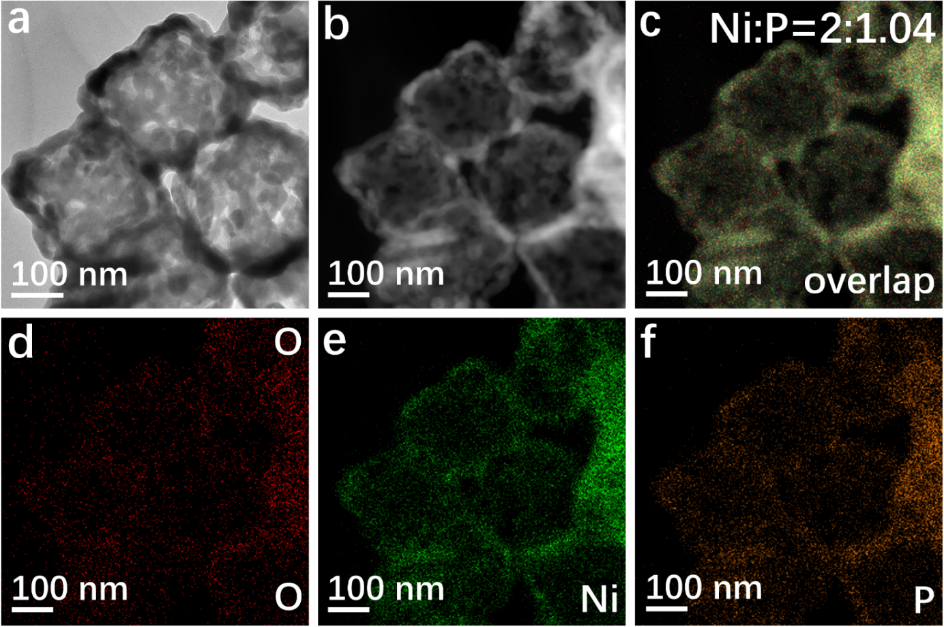


**Fig. S9** **a** TEM image, **b** HAADF-STEM image, and **c-f** corresponding elemental maps of the Ni_x_P_y_.


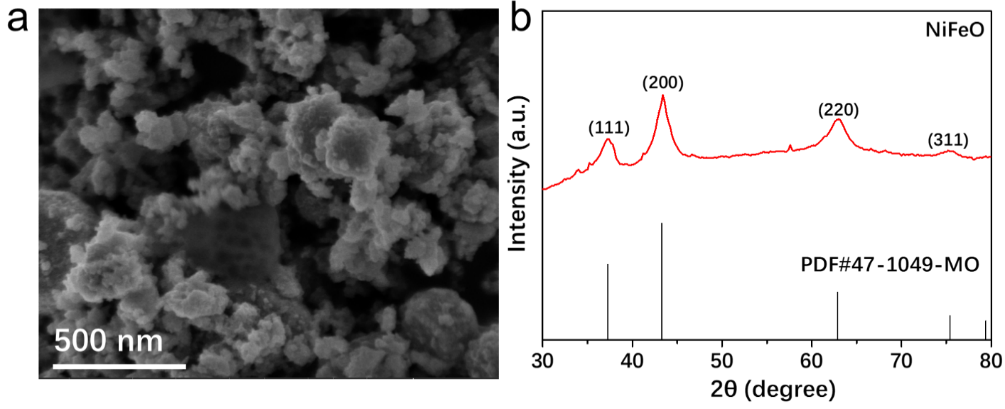


**Fig. S10 a** SEM image and **b** XRD patterns of NiFeO.


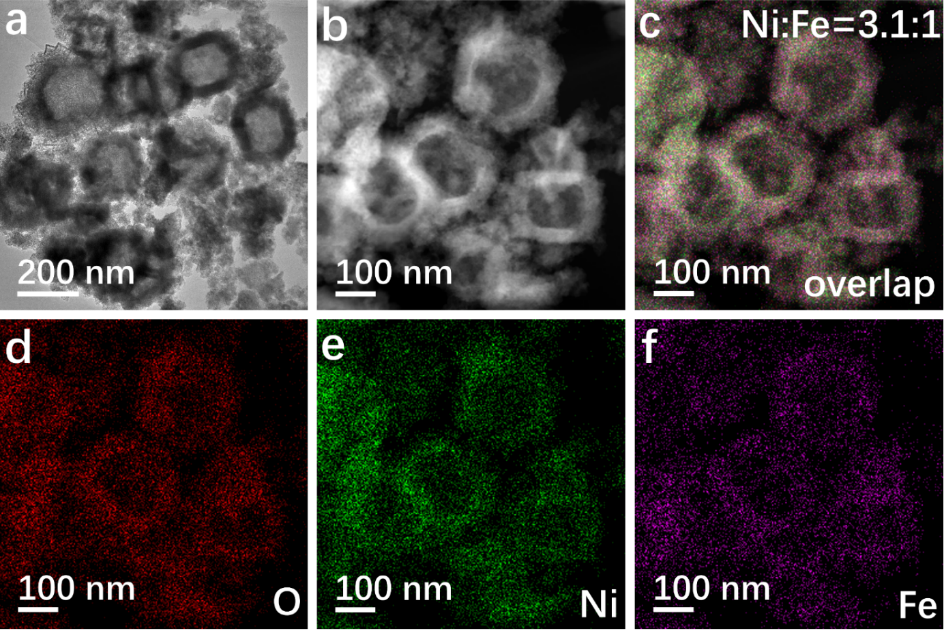


**Fig. S11** **a** TEM image, **b** HAADF-STEM image, and **c-f** corresponding elemental maps of the NiFeO.

**Fig. S12** LSV curves of Hg/HgO electrode vs. RHE.

The Hg/HgO reference electrode was calibrated using a standard three-electrode setup in 1.0 M KOH electrolyte, with a platinum counter electrode and a platinum mesh working electrode. Before measurement, the electrolyte was saturated with high-purity hydrogen (99.999%). Linear sweep voltammetry (LSV) was conducted at a scan rate of 2 mV s^-1^. All measurements were performed in triplicate to ensure reproducibility, as summarized in Fig. S12. The thermodynamic potential for the hydrogen electrode reaction was determined as the electrode potential at zero current. Based on the average of three independent measurements, the conversion relationship was established as *E*_(RHE)_ = *E*_(Hg/HgO)_ + 0.9153 V.

**
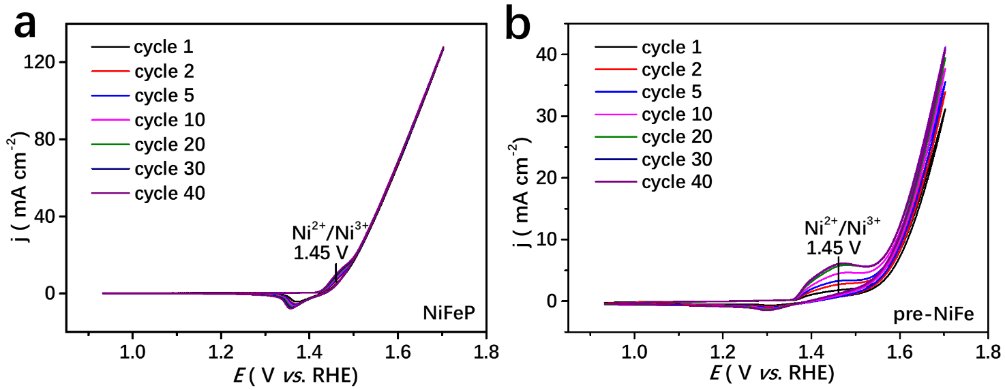
**

**Fig. S13** CV curves of the as-synthesized NiFeP and pre-NiFe.

**
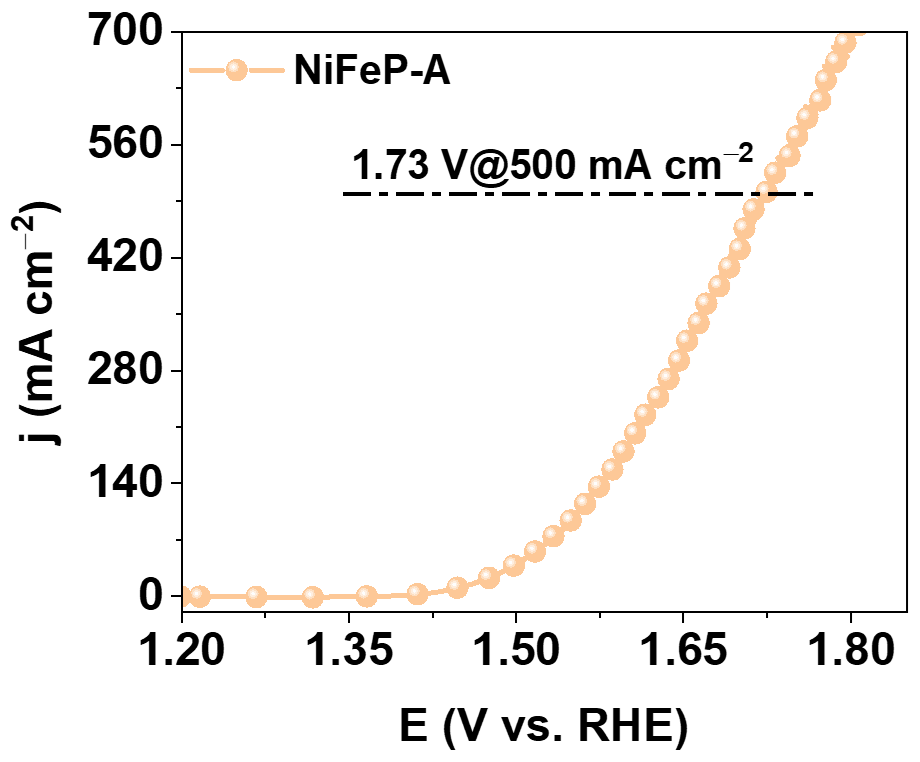
**

**Fig. S14** Polarization curves (backward-scan profiles) of NiFeP-A at the large current densities.
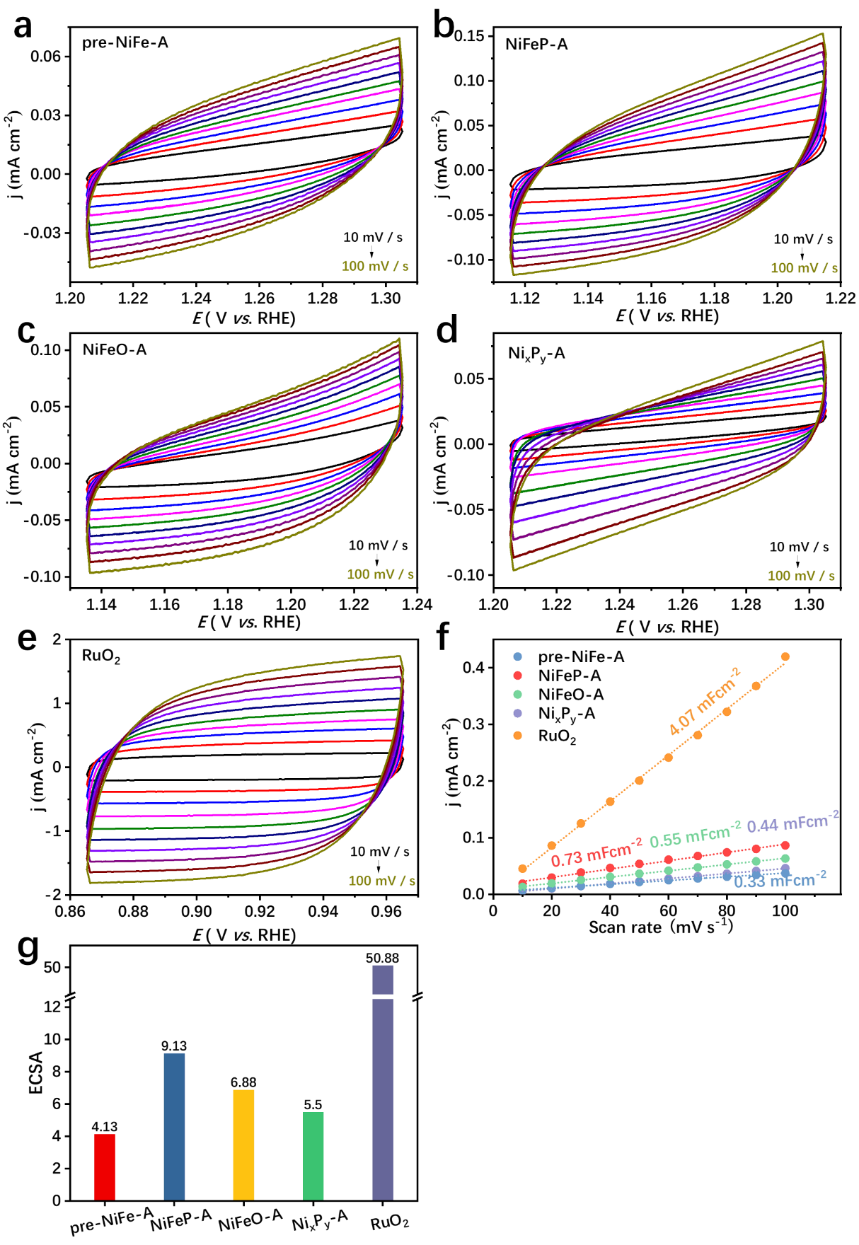


**Fig. S15** CV curves of the catalysts recorded in the non-Faradaic regions at scanning rates from 10 mV/s to 100 mV/s. **a** pre-NiFe-A. **b** NiFeP-A. **c** NiFeO-A. **d** Ni_x_P_y_-A. **e** RuO_2_. **f** Double-layer capacitances (C_dl_). **g** ECSA values.


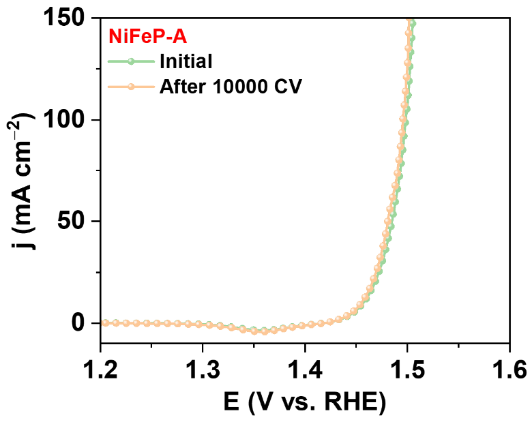


**Fig. S16** Polarization curves of NiFeP before and after 10000 cycles.


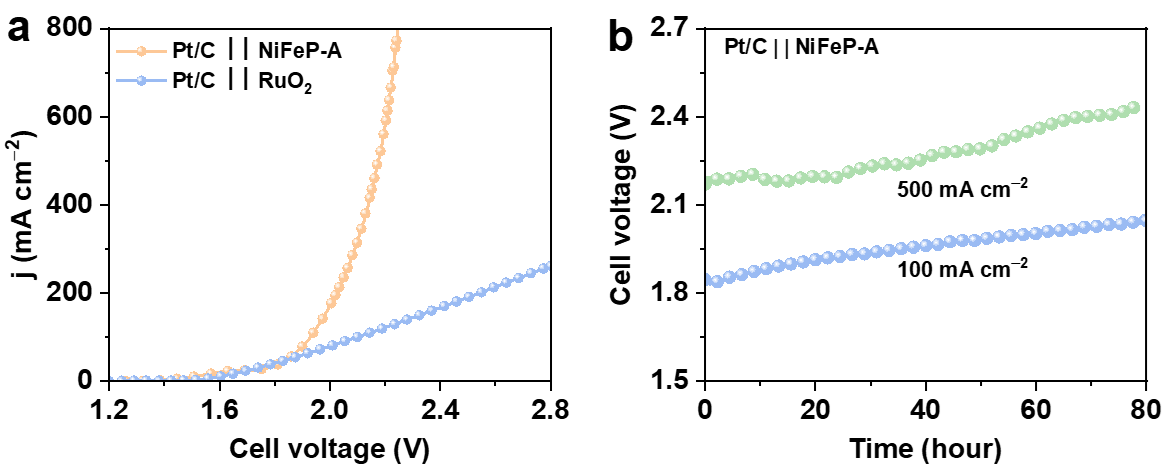


**Fig. S17** **a** LSV curves of the Pt/C || NiFeP-A two electrode system and commercial Pt/C || RuO_2_ system in 1 M KOH solution. **b** Stability tests of the Pt/C || NiFeP-A two electrode system at a current density of 100 mA cm^−2^ and 500 mA cm^−2^.

TOF calculation

TOF = $\frac{\text{Number of oxygen turnover}}{\text{Number of active sites}}$

Number of oxygen turnover (mol/s) = $\frac{\text{I}}{\text{4F}}$

Thus, TOF can be calculated with the equation:

TOF= $\frac{\text{I}}{\text{4Fn}}$

where I is the current obtained from the LSV tests at the overpotential of 300 mV, F is the Faradaic constant of 96485.3 C / mol.

The number of active sites (n) was determined by the total loading mass, which is in an underestimated way, via equation [S11]

Number of active sites (mol) = $\frac{\text{50 }\text{μ}\text{L*2 mg/200}\text{μ}\text{L}\text{×}\text{γ}_{\text{NiFe/NiFePOH}}}{\text{Mw}}$

Where γ_NiFe/NiFePOH_ is the mass ratio of NiFe in NiFeP-A (NiFePOH) (Ni:Fe:P:O = 0.77:0.23:013:2.49), M_w_ is the molecular weight of NiFeP-A.


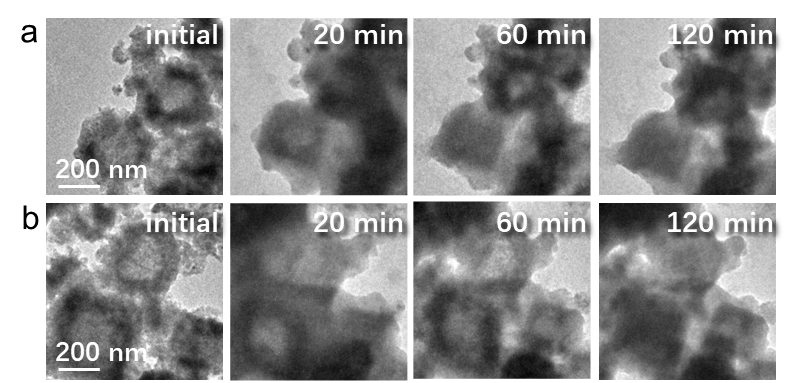


**Fig. S18** IL-TEM images of NiFeP before (left) and after 1.82 V_RHE_ for 20 min (middle), 60 min (middle), and 120 min (right) electrolysis in different areas.

**Fig. S19.** Comparison of Ni and Fe concentrations in fresh and NiFeP-activated alkaline media measured by ICP-MS.


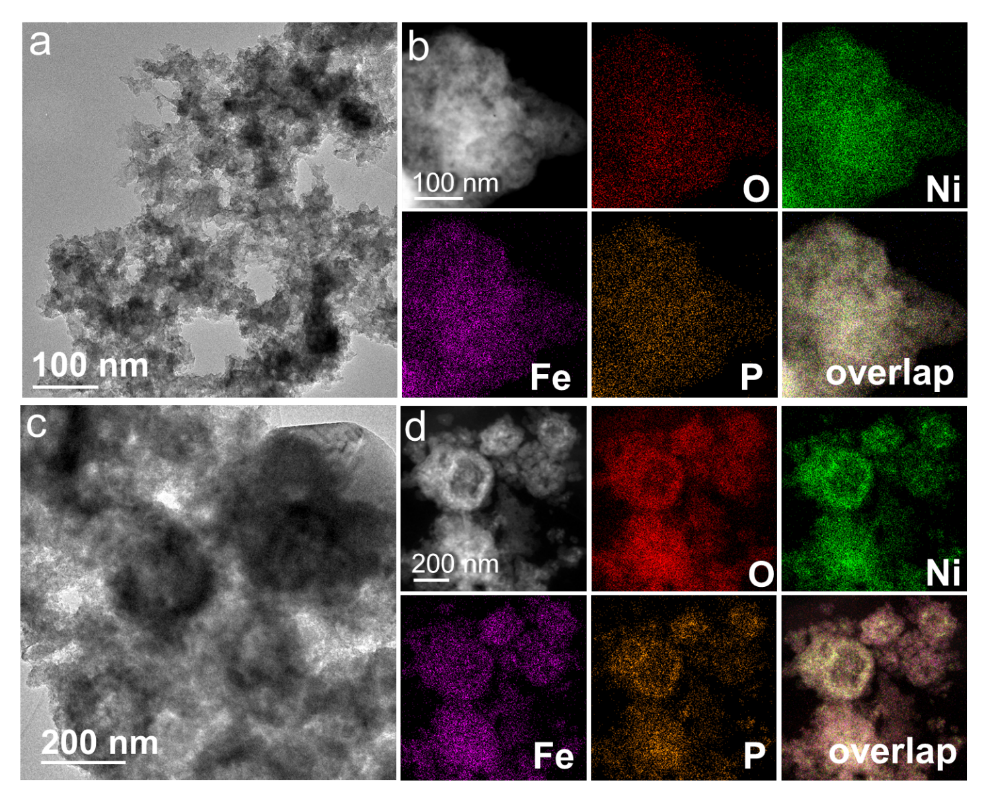


**Fig. S20** Morphological and compositional analyses of NiFeP after CV scans. **a, c** TEM images. **b, d** HAADF-STEM images and elemental maps.


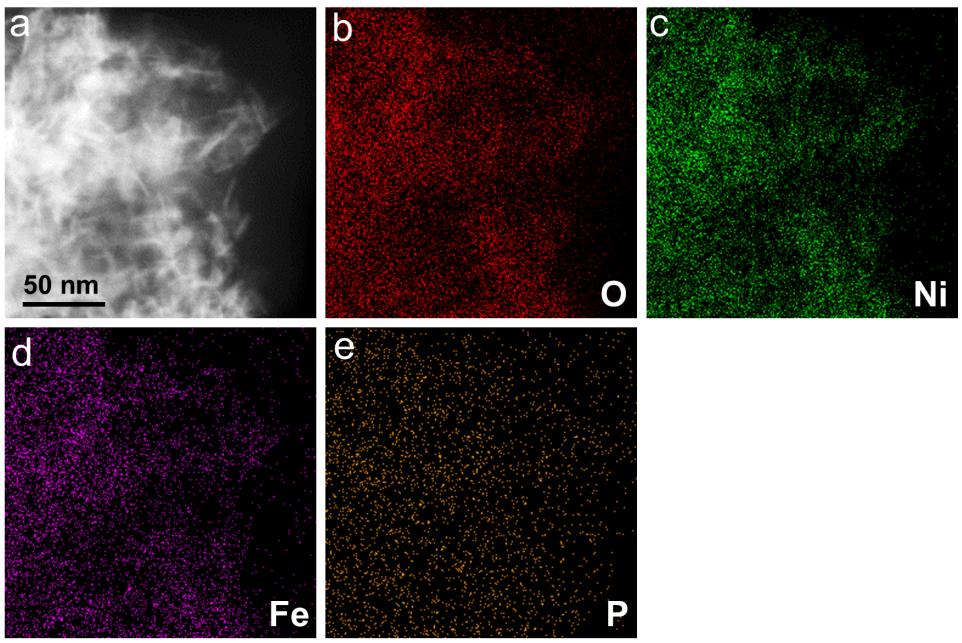


**Fig. S21** **a** HAADF-STEM image and **b-e** elemental maps of the NiFeP-A catalyst.


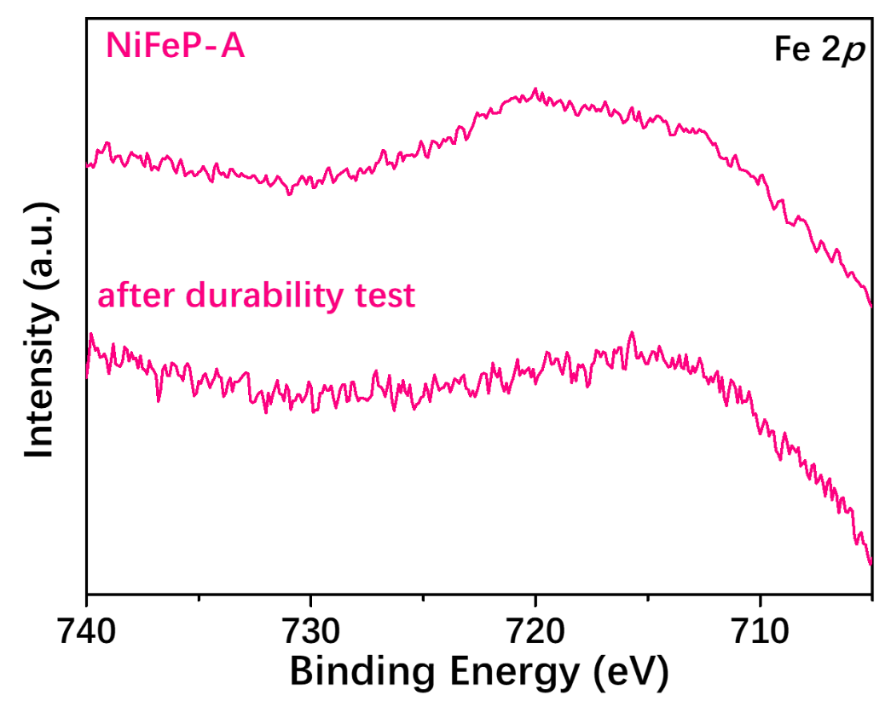


**Fig. S22** XPS spectra of NiFeP-A and after the durability test.


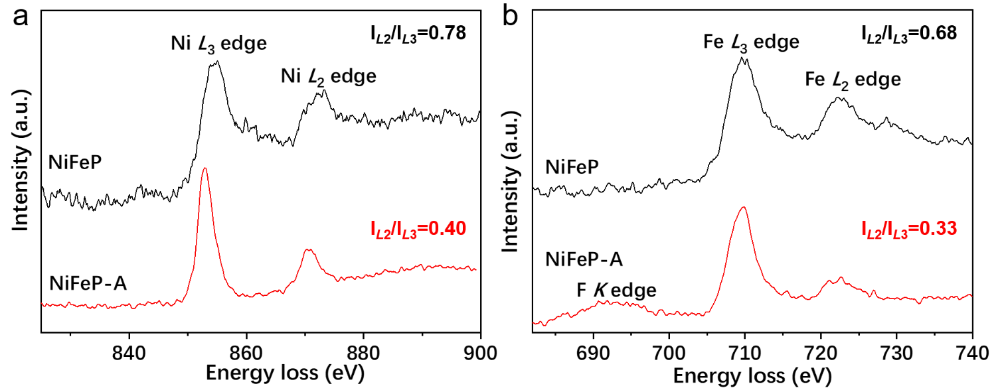


**Fig. S23** EELS analysis of **a** NiFeP and **b** NiFeP-A.


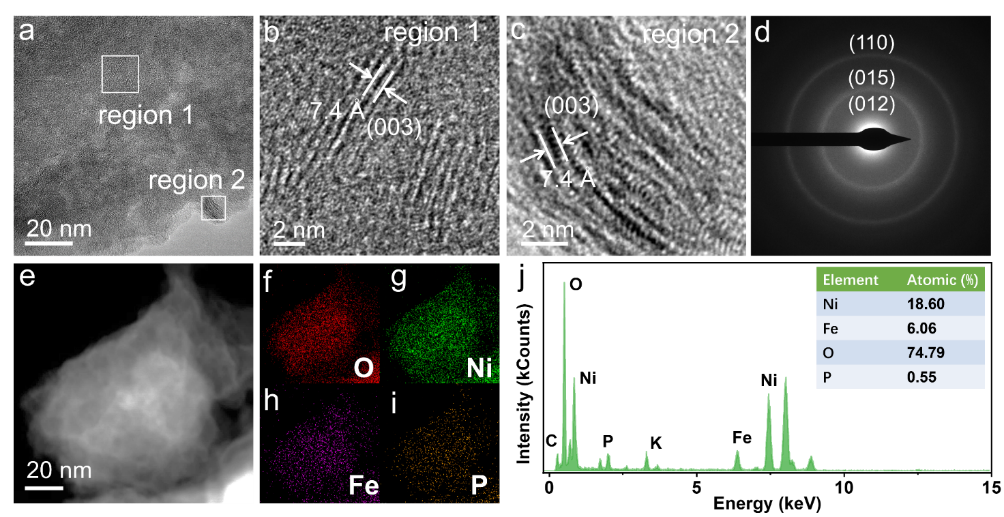


**Fig. S24** Structural and chemical analyses of the NiFeP-A after stability test. **a** HRTEM image. **b, c** Enlarged HRTEM images from the dotted squares indicated in **a**. **d** SAED patterns. **e** HAADF-STEM image and **e-i** corresponding elemental mappings. **j** EDX analysis of NiFeP-A after long-term stability experiments.


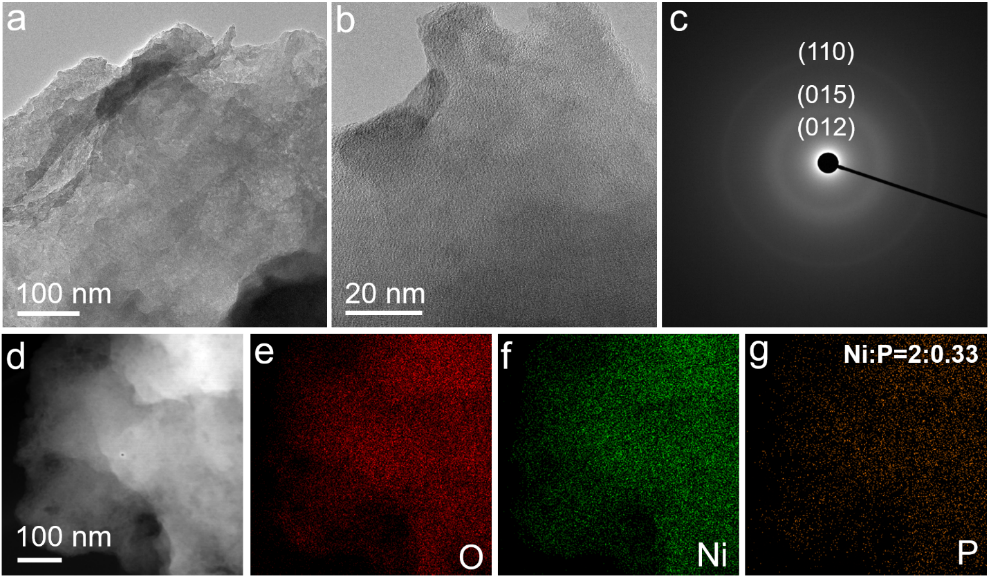


**Fig. S25** Structural and chemical analyses of the Ni_x_P_y_-A. **a** TEM image. **b** HRTEM image. **c** SAED pattern. **d-g** HAADF-STEM image and corresponding elemental mappings.


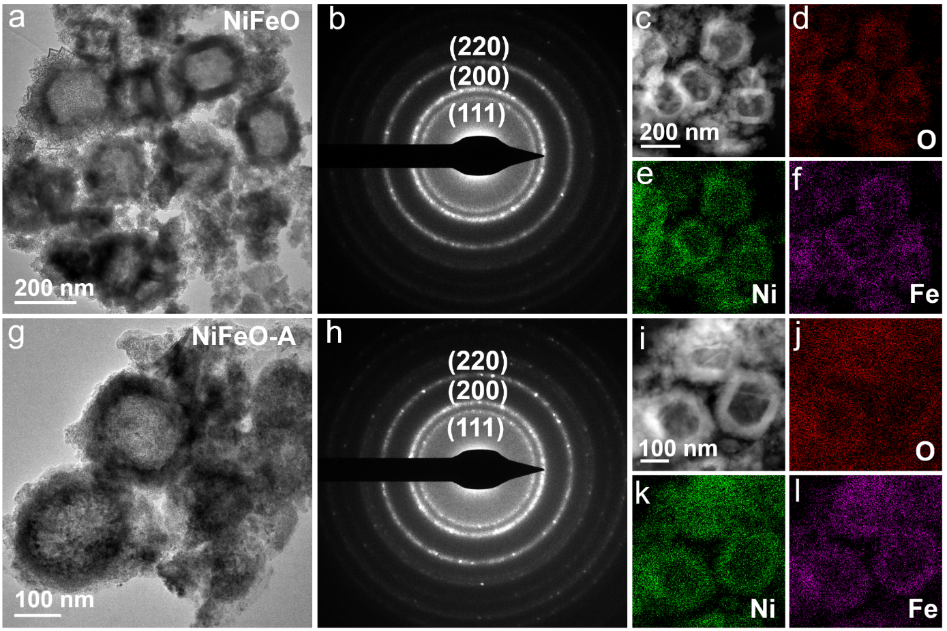


**Fig. S26** TEM images, SAED patterns, HAADF-STEM images, and corresponding elemental mappings of **a-f** NiFeO and **g-l** NiFeO-A.


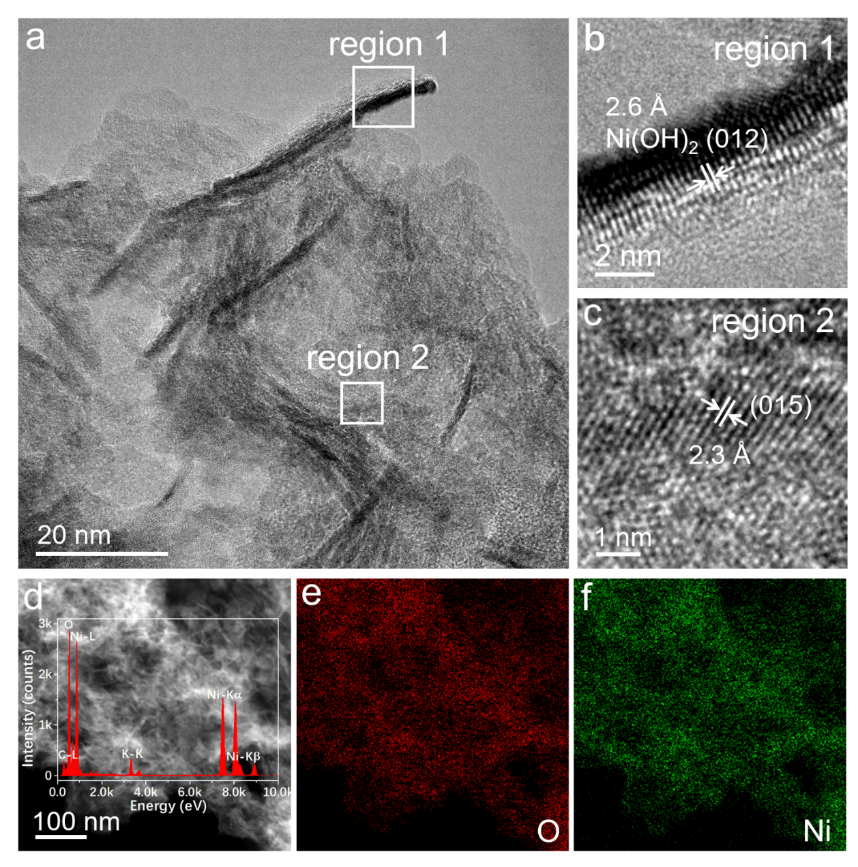


**Fig. S27** Structural analyses of the pre-NiFe-A. **a** HRTEM image. **b, c** Enlarged HRTEM images from the dotted squares indicated in **a**. **d-f** HAADF-STEM image and corresponding elemental maps. Inset of **d** shows an EDX spectrum of the sample, which demonstrates the absence of Fe signal in pre-NiFe-A.

**Fig. S28** Fe concentrations in basic media (1.0 mol/L KOH) before and after activation of pre-NiFe measured by ICP-MS.

**Control Experiment**

In order to determine whether iron is lost due to the heating process or the introduction of P, we synthesized pre-NiFe-Ar by heating pre-NiFe in an Ar atmosphere and conducted XRD, TEM, and OER test (see Figs. S29-S31). Pre-NiFe-Ar-A displayed a much inferior OER activity compared with NiFeP-A, indicating that the introduction of P played an important role in promoting electrocatalytic performance. Besides, TEM characterization of pre-NiFe-Ar-A (after CV and LSV activation) revealed that the morphology and structure remained unchanged, while EDX analysis showed that the initial atomic ratio of Ni:Fe (3.8:1) was preserved after activation, further indicating that Fe in pre-NiFe-Ar can be maintained through a heating process. Furthermore, by comparing pre-NiFe-Ar-A with NiFeP-A, we can conclude that the introduction of P could indeed induce the morphology changes.

**Synthesis of pre-NiFe-Ar**

The whole synthesis process is similar to that of NiFeP, except that Phosphorus sources are not applicable.

**Characterizations of pre-NiFe-Ar**

The morphology and structure of pre-NiFe-Ar are exhibited in Figs. S29 and S30. XRD patterns (Fig. S29) demonstrate that the crystallographic structure of the pre-NiFe-Ar can be assigned to the mixture of Ni_3_Fe (JCPDS 88-1715) and Ni_1.43_Fe_1.7_O_4_ (JCPDS 80-0072), which is due to the inevitable oxidation (oxygen in bulk pre-NiFe) of the air during the reaction. TEM images imply that pre-NiFe-Ar shows same morphology with pre-NiFe precursor, while EDX shows the Ni/Fe/O atomic ratio in pre-NiFe-Ar is 3.8:1:4.3 (Fig. S30).


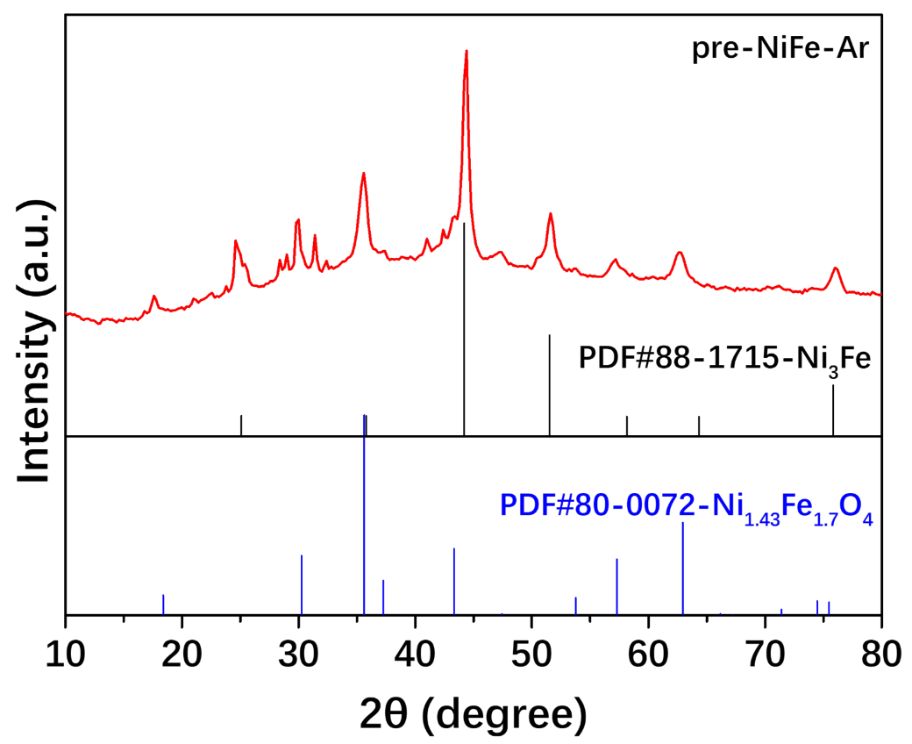


**Fig. S29** XRD patterns of pre-NiFe-Ar.


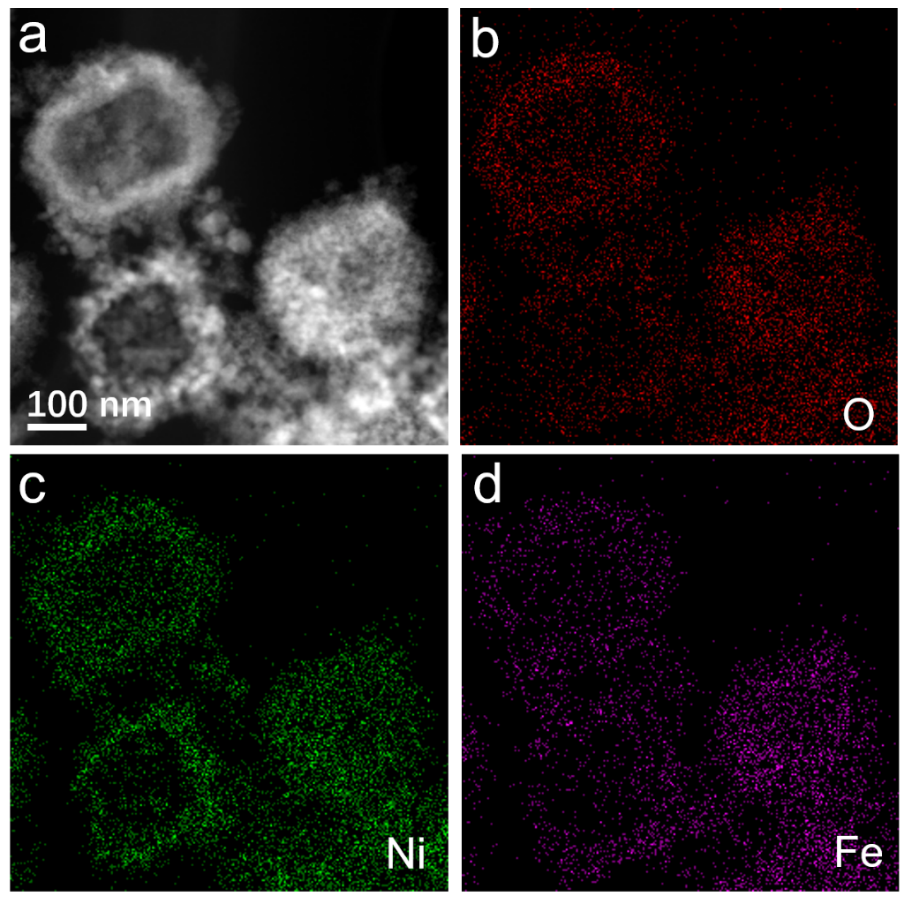


**Fig. S30** **a** HAADF-STEM image and **b-d** corresponding elemental maps of pre-NiFe-Ar.


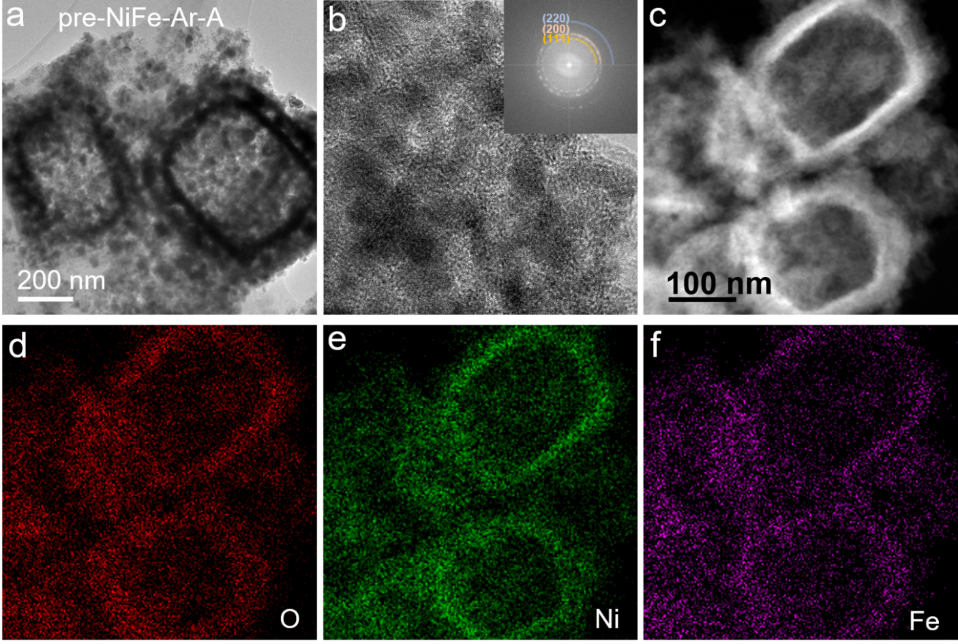


**Fig. S31** **a** TEM and **b** HRTEM images. Inset of b shows corresponding FFT image. **c** HAADF-STEM image and **d-f** corresponding elemental maps of pre-NiFe-Ar-A.


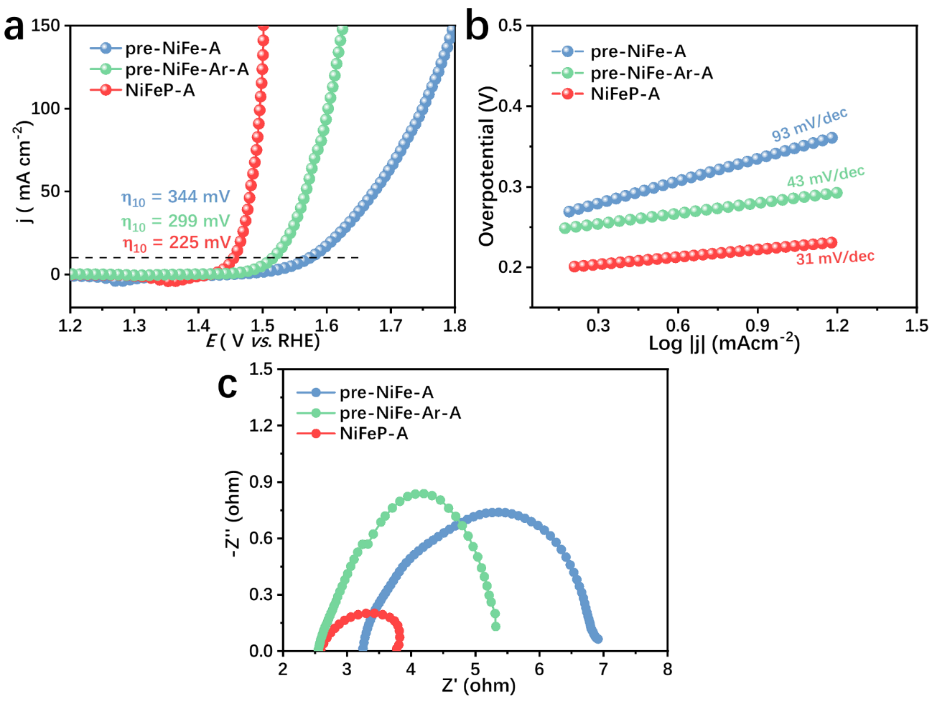


**Fig. S32** OER performance of pre-NiFe-A, pre-NiFe-Ar-A, and NiFeP-A in 1.0 M KOH. **a** Polarization curves. **b** Tafel plots. **c** The Nyquist plots.

**
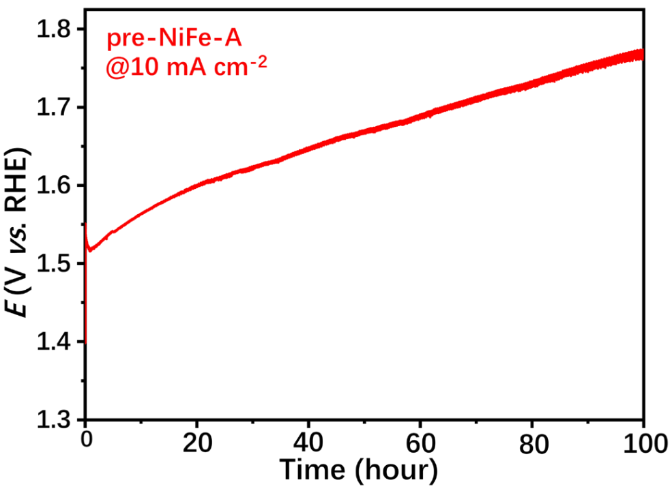
**

**Fig. S33** Stability test of pre-NiFe-A at the current density of 10 mA cm^−2^ for 100 h.

As shown in Figs. S32 and S33, the pre-NiFe-Ar-A showed a higher overpotential (η_10_ = 299 mV), a higher Tafel slope (43.0 mV dec⁻^1^), and a larger charge-transfer resistance (2.97 ohm) compared to NiFeP-A. These results demonstrated that the pre-NiFe-Ar-A had much inferior OER activity compared with NiFeP-A, indicating that the introduction of P played an important role in electrocatalytic performance. Moreover, TEM showed the morphology and composition of pre-NiFe-Ar-A were preserved after CV and LSV activation (Fig. S31), demonstrating that Fe in pre-NiFe can be retained through heating process other than phosphating process.

**
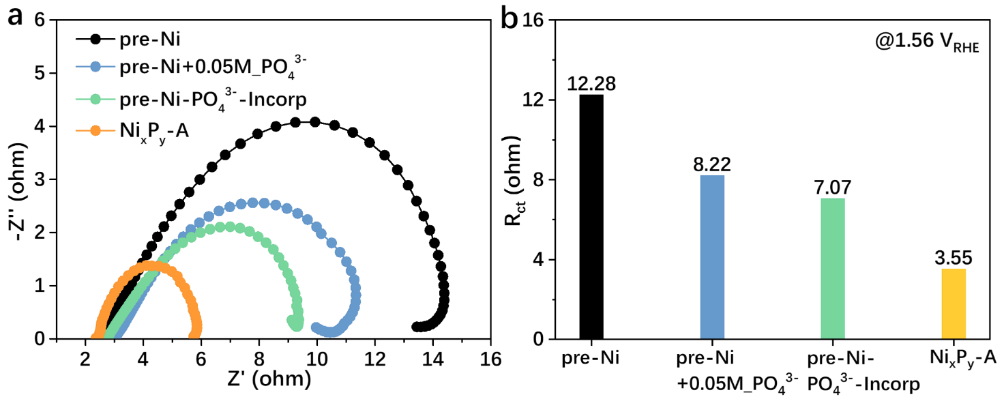
**

**Fig. S34** **a, b** Comparison of Nyquist plots at 1.56 V *vs.* RHE of pre-Ni in 1.0 M KOH electrolyte with 0.05 M concentrations of PO_4_^3^⁻ under different pretreatments.


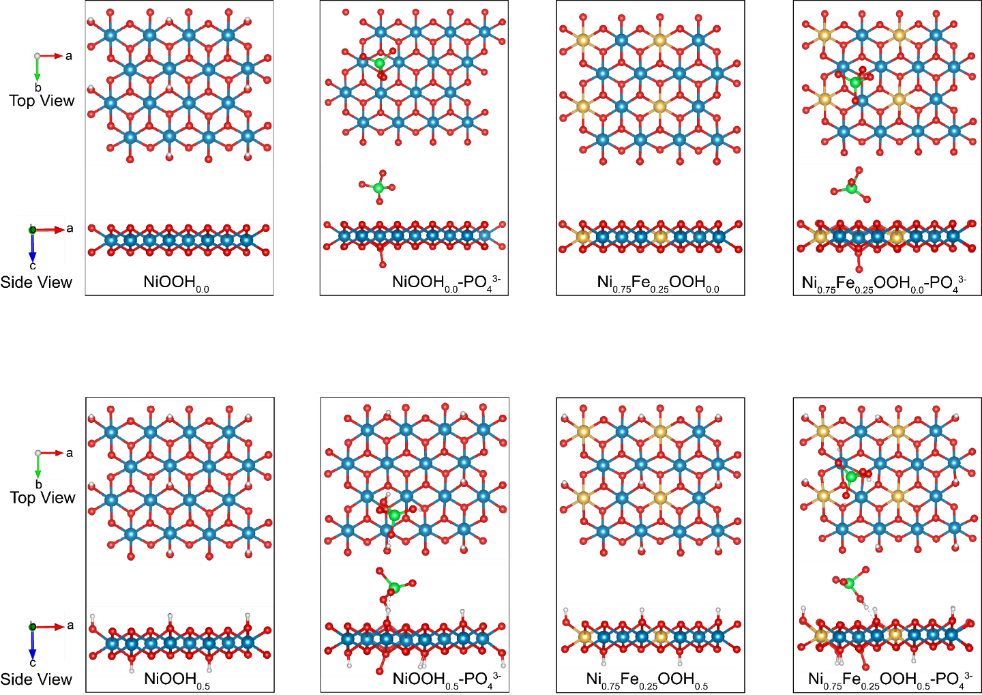


**Fig. S35** The optimal atom model. Atom coloring: Ni: blue, Fe: yellow, O: red, H: grey, P: green.


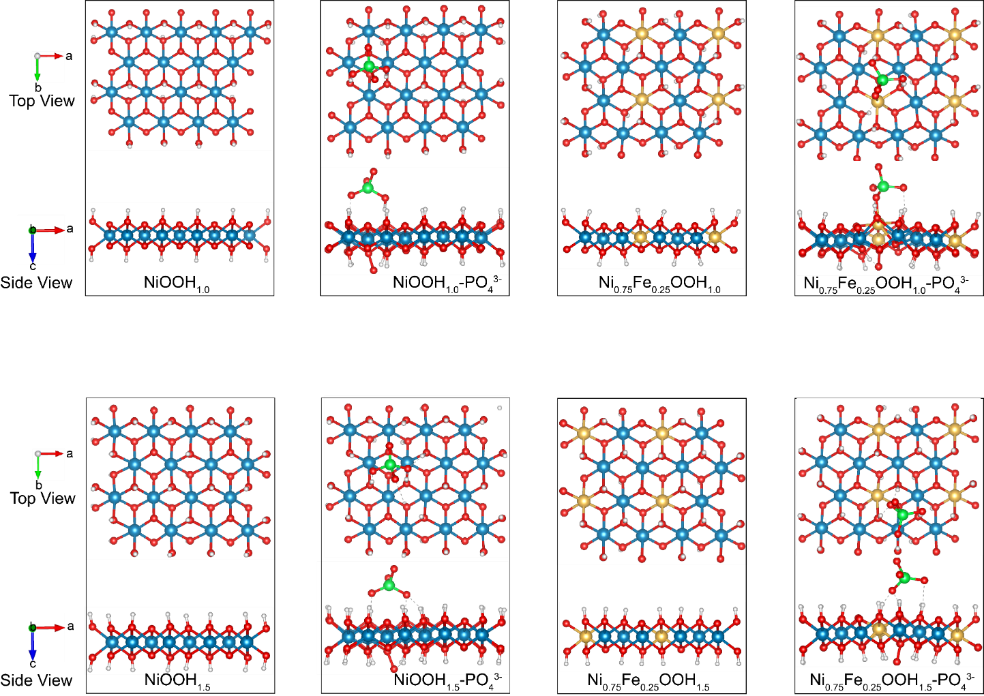


**Fig. S36** The optimal atom model. Atom coloring: Ni: blue, Fe: yellow, O: red, H: grey, P: green.


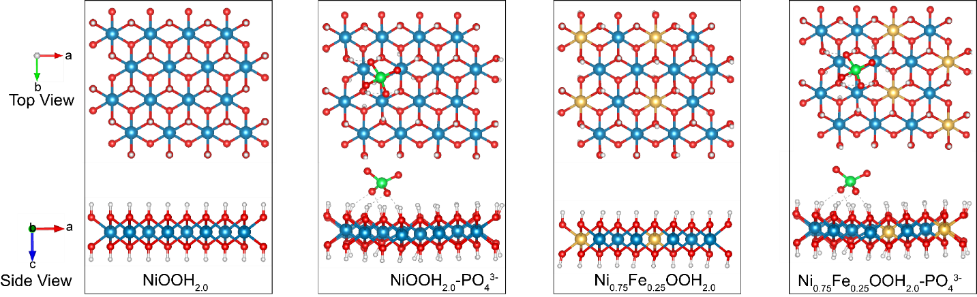


**Fig. S37** The optimal atom model. Atom coloring: Ni: blue, Fe: yellow, O: red, H: grey, P: green.


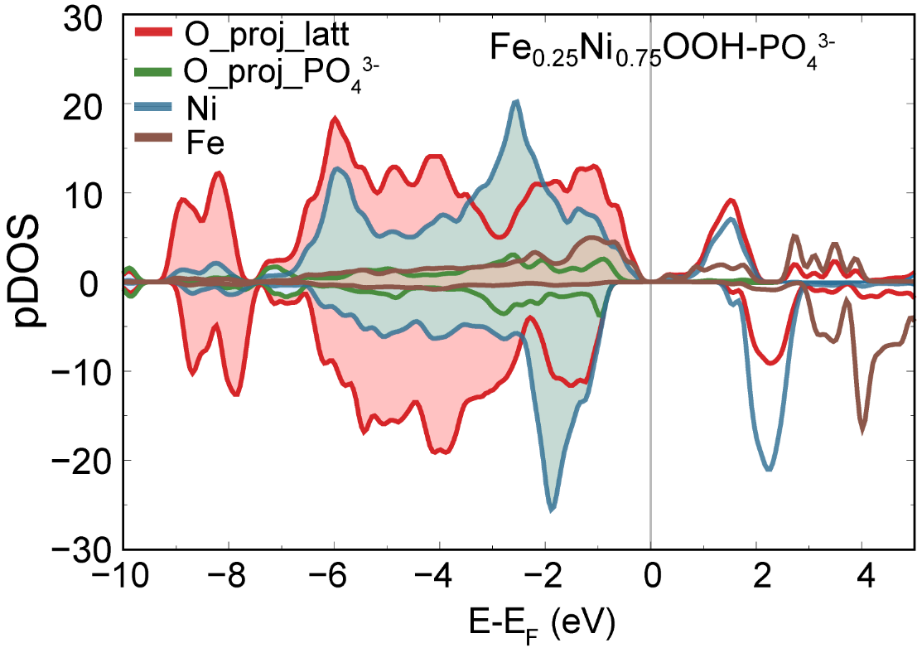


**Fig. S38** Projected density of states (pDOS) for Ni_0.75_Fe_0.25_OOH-PO_4_^3^⁻.

**
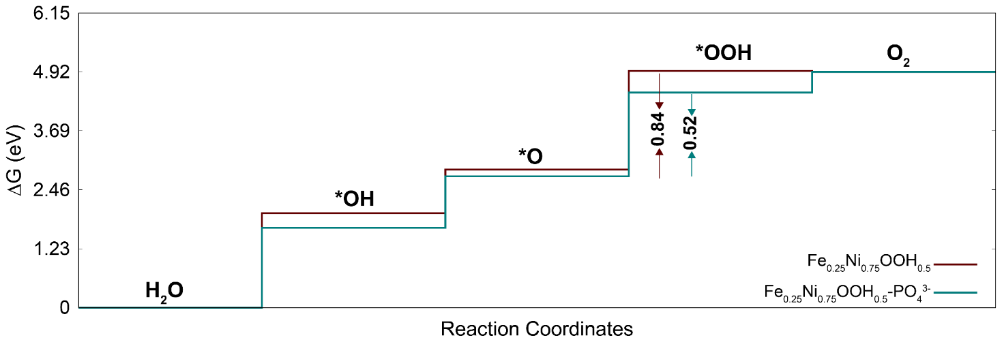
**

**Fig. S39** The free energy profile of Fe_0.25_Ni_0.75_OOH_0.5_ and Fe_0.25_Ni_0.75_OOH_0.5_- PO_4_^3^⁻.

**
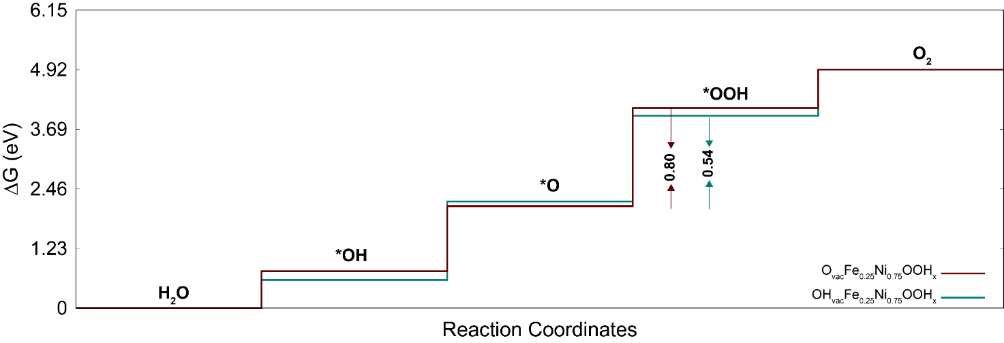
**

**Fig. S40** The free energy profile of OH_vac_ - Fe_0.25_Ni_0.75_OOH_x_ and O_vac_ - Fe_0.25_Ni_0.75_OOH_x_.

**
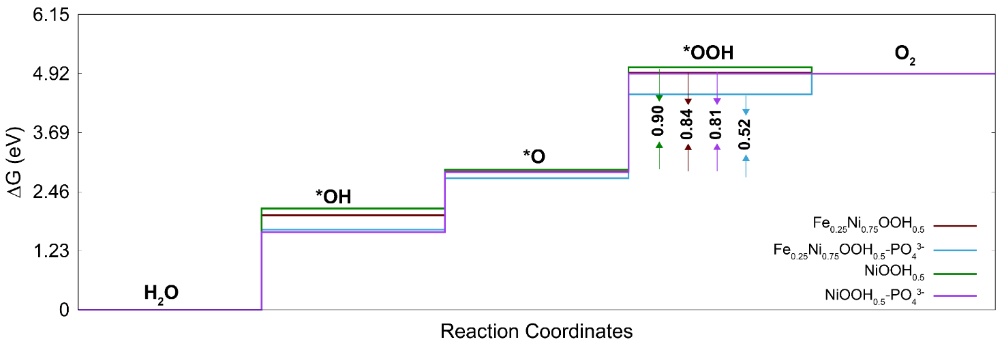
**

**Fig. S41** The free-energy profiles for Fe_0.25_Ni_0.75_OOH_0.5_, Fe_0.25_Ni_0.75_OOH_0.5_-PO_4_^3^⁻_,_ NiOOH_0.5_ and NiOOH_0.5_-PO_4_^3^⁻.

**Table S1** Elemental analysis of catalysts obtained using different amounts of phosphorus precursor (NaH_2_PO_2_) and after CV+LSV measurements

| Catalyst | Ni | Fe | P | O |
| --- | --- | --- | --- | --- |
| NiFeP-A-0.2 g (NiFeP-A) | 22.06% | 7.41% | 0.83% | 69.69% |
| NiFeP-A-0.1 g | 22.73% | 7.36% | 0.15% | 69.76% |
| NiFeP-A-1.0 g | 25.14% | 8.32% | 1.44% | 65.10% |
| NiFeP-A-2.0 g | 22.86% | 7.38% | 2.36% | 67.54% |

**Table S2** ICP-OES of NiFeP

| Element | Ni | Fe | P |
| --- | --- | --- | --- |
| Mass ratio in NiFeP, % | 33.97% | 10.78% | 24.67% |
| Molar ratio in NiFeP (normalized to Fe) | 3 | 1 | 4.13 |

**Table S3** Comparison of the overpotential (at 500 mA cm^−2^) of our catalyst with recently reported OER catalysts in 1.0 M KOH

| Catalysts | Overpotential @500 mAcm^−2^ | Ref. |
| --- | --- | --- |
| **NiFeP-A** | **500** | **This work** |
| αβ1-Ni(OH)_2_ | 590 | [S12] |
| NiCo/NF | 507 | [S13] |
| FeS@Fe_2_B/IP | 520 | [S14] |
| α-Co(OH)_2_ | 630 | [S15] |
| Co-CoO_x_@NOC-0.1KNO_3_ | 612 | [S16] |
| FeCoNiMoW | 600 | [S17] |
| FeCoCrNiTi_0.6_ (HEA) | 655 | [S18] |
| NiS-FeS@IF | 563 | [S19] |
| CeO_2_-NiFeO_x_H_y_ | 659 | [S20] |
| POM-CTAB-Co | 770 | [S21] |
| P-NiO | 560 | [S22] |
| CD@Ni_0.6_Fe_0.4_OOH/CP | 750 | [S23] |

**Table S4** Comparison of the overpotential (at 10 mA cm^-2^) and the Tafel slope of NiFeP-A with recently reported NiFeP OER catalysts in 1.0 M KOH

| Catalysts | Overpotential (10 Ma cm^-2^) | Tafel slope (mV/dec) | Ref. |
| --- | --- | --- | --- |
| **NiFeP-A** | **225** | **32** | **(This work)** |
| NiFeP/g-C_3_N_4_ | 232 | 103 | [S24] |
| NiFeP/FP-2 | 235 | 114.26 | [S25] |
| NiFeP**-**Sn/NM | 239 | 52.1 | [S26] |
| (Ni_0.75_Fe_0.25_)_2_P@MXene | 240 | 65.5 | [S27] |
| NiFeP/NPC OHS | 243 | 57 | [S28] |
| YRO@D-NiFeP/Ru | 260 | 42.4 | [S29] |
| La_0.3_-NiFeP | 271 | 78 | [S30] |
| NiFeP/CoP | 274 | 70 | [S31] |
| NiFeP@FPs | 282 | 63.41 | [S32] |
| NiFeP/MXene | 286 | 35 | [S33] |
| NiFeP/NiS-A | 295 | 52.3 | [S34] |

**Table S5** Fitting parameters of the electrochemical impedance spectra of as-prepared catalysts for OER measured at an overpotential of 330 mV

| Catalysts | η (mV) | R_s_ (ohm) | R_ct_ (ohm) |
| --- | --- | --- | --- |
| pre-NiFe-A | 330 | 3.21 | 3.80 |
| NiFeP-A | 330 | 2.54 | 1.36 |
| NiFeO-A | 330 | 2.97 | 2.09 |
| Ni_x_P_y_-A | 330 | 2.41 | 3.55 |
| RuO_2_ | 330 | 2.71 | 9.05 |

ƞ: overpotential

R_s_: The solution and electrode resistance

R_ct_: The charge transfer resistance

**Table S6** TOFs of pre-NiFe-A, NiFeP-A, NiFeO-A, Ni_x_P_y_-A, and RuO_2_

| Catalysts | Molecular formula | Molecular weight | Mass ratio | n (mol) | j (mA) | TOF (s^-1^) |
| --- | --- | --- | --- | --- | --- | --- |
| pre-NiFe-A | Ni_1_O_2.91_ | 105.25 | 0.56 | 2.66×10^-6^ | 1.701 | 0.0017 |
| NiFeP-A | Ni_0.77_Fe_0.23_P_0.13_O_2.49_ | 101.90 | 0.57 | 2.80×10^-6^ | 153.4 | 0.14 |
| NiFeO-A | Ni_0.76_Fe_0.24_O_1_ | 74.01 | 0.78 | 5.27×10^-6^ | 5.254 | 0.0026 |
| Ni_x_P_y_-A | Ni_1_P_0.17_O_2.64_ | 106.19 | 0.55 | 2.59×10^-6^ | 5.675 | 0.0057 |
| RuO_2_ | RuO_2_ | 133.10 | 0.76 | 2.86×10^-6^ | 7.935 | 0.0072 |

**Table S7** Comparison of TOF between NiFeP-A and recently reported OER catalysts in 1.0 M KOH.

| Catalysts (1.0 M KOH) | Overpotential (mV) | TOF | Ref. |
| --- | --- | --- | --- |
| **NiFeP-A** | **300** | **0.14** | **This work** |
| RuO_2_ | 300 | 0.0072 | This work |
| NiFe-OH/NiFeP/NF | 250 | 0.036 | [S35] |
| NiFeP@OCC | 400 | 0.018 | [S36] |
| H-CoS_x_@NiFe LDH/NF | 280 | 0.067 | [S37] |
| Ni_3_V_1_Fe_1_-LDH | 300 | 0.0528 | [S38] |
| Ni_0.75_V_0.25_-LDH | 350 | 0.054 | [S39] |
| RuNi_7_FeO_x_(OH)_y_ @NCA | 278 | 0.107 | [S40] |
| Ru@Fe-Ni (OH)_2_/NF | 300 | 0.062 | [S41] |
| Fe_3_O_4_-NF | 300 | 0.00312 | [S42] |
| Co_0.5_V_0.5_@COF-SO_3_ | 300 | 0.098 | [S43] |
| Cu-ZIF-400 | 342 | 0.0021 | [S44] |
| Co_3_O_4_-TiO_2_ | 370 | 0.034 | [S45] |

**Table S8** Comparison of cell voltages at 10 mAcm^−2^ with state-of-the-art overall water splitting electrocatalysts in 1.0 M KOH

| Catalysts | Cell voltage | Ref. |
| --- | --- | --- |
| **Pt/C \|\| NiFeP-A** | **1.51 V** | **This work.** |
| Pt/C \|\| RuO_2_ | 1.56 V | This work. |
| MoP/NiFeP/CP \|\| MoP/NiFeP/CP | 1.51 V | [S46] |
| NiP/NiFeP/C \|\| NiP/NiFeP/C | 1.53 V | [S47] |
| CC-NC-NiFeP \|\| CC-NC-NiFeP | 1.54 V | [S48] |
| Mo-NiFeP/NIF-2 \|\| Mo-NiFeP/NIF | 1.55 V | [S49] |
| Pt/C \|\| Mo_51_Ni_40_Fe_9_ | 1.56 V | [S50] |
| Ni_0.8_Fe_0.2_P-C/NF \|\| Ni_0.8_Fe_0.2_P-C/NF | 1.56 V | [S51] |
| CoP-NC@NFP \|\| CoP-NC@NFP | 1.57 V | [S52] |
| NiFeP/CC\|\| NiFeP/CC | 1.57 V | [S53] |
| NiFeP/NFF \|\| NiFeP/NFF | 1.58 V | [S54] |
| Pt/C \|\| NiFeP/MXene | 1.61 V | [S33] |
| NiFeP/Zn \|\| NiFeP/Zn | 1.65 V | [S55] |
| Pt/C \|\| Fe-NiCoP | 1.71 V | [S56] |

**Table S9** ICP-OES of NiFeP-A

| Element | Ni | Fe | P |
| --- | --- | --- | --- |
| Metallic mass ratio in NiFeP-A, % | 15.41% | 4.35% | 1.16% |
| Metallic molar ratio in NiFeP-A (normalized to Fe) | 3.37 | 1 | 0.48 |

**Table S10** The EXAFS fitting data of NiFeP and NiFeP-A at the Ni K-edge

| Sample | Path | *CN* | *R*(Å) | *σ*^2^(×10^−3^Å^2^*)* | Δ*E*_0_ (eV) | *R*-factor |
| --- | --- | --- | --- | --- | --- | --- |
| Ni foil | Ni−Ni | 12* | 2.48±0.03 | 5.9±0.3 | 7.1±0.4 | 0.0022 |
| NiO | Ni−O | 7.9±1.3 | 2.08±0.02 | 6.1±2.4 | -2.9±0.9 | 0.0084 |
|  | Ni−Ni | 17.2±2.1 | 2.95±0.01 | 7.0±0.9 | -2.9±0.9 |  |
| NiFeP | Ni−P | 7.1±0.6 | 2.24±0.01 | 10.1±1.3 | -6.2±1.2 | 0.0066 |
| NiFeP-A | Ni−O | 7.3±0.4 | 2.02±0.08 | 10.1±1.1 | -6.2±0.7 | 0.0047 |
|  | Ni−(O) −M | 4.1±0.2 | 3.04±0.08 | 4.4±3.5 | -6.1±0.7 |  |

*CN,* coordination number. *R*, the distance to the neighboring atom. *σ*^2^, Debye-Waller factors. Δ*E*_0_, inner potential correction. The *R*-factor indicates the goodness of the fit. *S*_0_^2^ was fixed to 0.75 according to the experimental EXAFS fit of Ni foil by fixing *CN* as the known crystallographic value. * This value was fixed during EXAFS fitting.

Fitting range: 3.0 ≤ k (/Å) ≤ 12.5 and 1.0 ≤ R (Å) ≤ 3.0 (Ni foil).

3.0 ≤ k (/Å) ≤ 12.4 and 1.0 ≤ R (Å) ≤ 3.0 (NiO).

3.0 ≤ k (/Å) ≤ 12.4 and 1.2 ≤ R (Å) ≤ 2.5 (NiFeP).

3.0 ≤ k (/Å) ≤ 12.3 and 1.0 ≤ R (Å) ≤ 3.5 (NiFeP-A).

A reasonable range of EXAFS fitting parameters: 0.700 < *S*_0_^2^ < 1.000; *CN* > 0; *σ*^2^ > 0 Å^2^; |Δ*E*_0_| < 10 eV; *R*-factor < 0.02.

**Table S11** Calculated Gibbs free energies and overpotential of OER on NiOOH_0.5_ and NiOOH_0.5_- PO_4_^3-^

| **Structure** | **Reaction step** | **∆G (eV)** | **Overpotential (V)** |
| --- | --- | --- | --- |
| **Ni_4_O_8_H_2_** | *+H_2_O(l) → *OH + (H^+^ + e^-^) | 2.11 | 0.90 |
|  | *OH → *O + (H^+^ + e^-^) | 0.81 |  |
|  | *O + H_2_O(l) → *OOH + (H^+^ + e^-^) | **2.13** |  |
|  | *OOH → * + O_2_ + (H^+^ + e^-^) | -0.13 |  |
| **Ni_4_O_8_H_2_ - PO_4_^3-^** | *+H_2_O → *OH + (H^+^ + e^-^) | 1.62 | 0.81 |
|  | *OH → *O + (H^+^ + e^-^) | 1.25 |  |
|  | *O + H_2_O(l) → *OOH + (H^+^ + e^-^) | **2.04** |  |
|  | *OOH → * + O_2_ + (H^+^ + e^-^) | 0.01 |  |

*Bold digits indicate ∆G _max._

**Table S12** Calculated Gibbs free energies and overpotential, η (V) of OER on Fe doped NiOOH_0.5_ and defective Fe doped NiOOH_0.5_

| **Structure** | **Reaction step** | | **∆G (eV)** | | **Overpotential (V)** | | |
| --- | --- | --- | --- | --- | --- | --- | --- |
| **Fe_0.25_Ni_0.75_OOH_0.5_** | | *+H_2_O(l) → *OH + (H^+^ + e^-^) | | 1.97 | | 0.84 |  |
|  |  | *OH → *O + (H^+^ + e^-^) | | 0.91 | |  |  |
|  |  | *O + H_2_O(l) → *OOH + (H^+^ + e^-^) | | **2.07** | |  |  |
|  |  | *OOH → * + O_2_ + (H^+^ + e^-^) | | -0.02 | |  |  |
| **Fe_0.25_Ni_0.75_OOH_0.5_- PO_4_^3-^** | | *+H_2_O(l) → *OH + (H^+^ + e^-^) | | 1.67 | | 0.52 |  |
|  |  | *OH → *O + (H^+^ + e^-^) | | 1.07 | |  |  |
|  |  | *O + H_2_O(l) → *OOH + (H^+^ + e^-^) | | **1.75** | |  |  |
|  |  | *OOH → * + O_2_ + (H^+^ + e^-^) | | 0.43 | |  |  |
| **OH_vac_- Fe_0.25_Ni_0.75_OOH_x_** | | *+H_2_O(l) → *OH + (H^+^ + e^-^) | | 0.58 | | 0.54 |  |
|  |  | *OH → *O + (H^+^ + e^-^) | | 1.62 | |  |  |
|  |  | *O + H_2_O(l) → *OOH + (H^+^ + e^-^) | | **1.77** | |  |  |
|  |  | *OOH → * + O_2_ + (H^+^ + e^-^) | | 0.95 | |  |  |
| **O_vac_ - Fe_0.25_Ni_0.75_OOH_x_.** | | *+H_2_O(l) → *OH + (H^+^ + e^-^) | | 0.76 | | 0.80 |  |
|  |  | *OH → *O + (H^+^ + e^-^) | | 1.34 | |  |  |
|  |  | *O + H_2_O(l) → *OOH + (H^+^ + e^-^) | | **2.03** | |  |  |
|  |  | *OOH → * + O_2_ + (H^+^ + e^-^) | | 0.79 | |  |  |

*Bold digits indicate ∆G _max._

**Supplementary References**

1. C.C.L. McCrory, S. Jung, J.C. Peters, T.F. Jaramillo, Benchmarking heterogeneous electrocatalysts for the oxygen evolution reaction. J. Am. Chem. Soc. **135**(45), 16977–16987 (2013). <https://doi.org/10.1021/ja407115p>
2. P. Giannozzi, S. Baroni, N. Bonini, M. Calandra, R. Car et al., QUANTUM ESPRESSO: a modular and open-source software project for quantum. J. Phys. Condens. Matter **21**(39), 395502 (2009). <https://doi.org/10.1088/0953-8984/21/39/395502>
3. Z.K. Goldsmith, A.K. Harshan, J.B. Gerken, M. Vörös, G. Galli et al., Characterization of NiFe oxyhydroxide electrocatalysts by integrated electronic structure calculations and spectroelectrochemistry. Proc. Natl. Acad. Sci. USA **114**(12), 3050–3055 (2017). <https://doi.org/10.1073/pnas.1702081114>
4. G. Prandini, A. Marrazzo, I.E. Castelli, N. Mounet, E. Passaro, et al., A Standard Solid State Pseudopotentials (SSSP) library optimized for p recision and efficiency, Materials Cloud Archive, 65 (2023). <https://doi.org/10.24435/materialscloud:f3-ym>
5. D.R. Hamann, Optimized norm-conserving Vanderbilt pseudopotentials. Phys. Rev. B **88**(8), 085117 (2013). <https://doi.org/10.1103/physrevb.88.085117>
6. J.K. Nørskov, J. Rossmeisl, A. Logadottir, L. Lindqvist, J.R. Kitchin et al., Origin of the overpotential for oxygen reduction at a fuel-cell cathode. J. Phys. Chem. B **108**(46), 17886–17892 (2004). <https://doi.org/10.1021/jp047349j>
7. H. Xu, D. Cheng, D. Cao, X.C. Zeng, Revisiting the universal principle for the rational design of single-atom electrocatalysts. Nat. Catal. **7**(2), 207–218 (2024). <https://doi.org/10.1038/s41929-023-01106-z>
8. J. Rossmeisl, A. Logadottir, J.K. Nørskov, Electrolysis of water on (oxidized) metal surfaces. Chem. Phys. **319**(1–3), 178–184 (2005). <https://doi.org/10.1016/j.chemphys.2005.05.038>
9. R.D.J. III, NIST Computational Chemistry Comparison and Benchmark Database, NIST Standard Reference Database, 101 (2022). <http://cccbdb.nist.gov/>
10. M. Li, L. Zhang, Q. Xu, J. Niu, Z. Xia, N-doped graphene as catalysts for oxygen reduction and oxygen evolution reactions: Theoretical considerations. J. Catal. **314**, 66–72 (2014). <https://doi.org/10.1016/j.jcat.2014.03.011>
11. P. Su, W. Pei, X. Wang, Y. Ma, Q. Jiang et al., Exceptional electrochemical HER performance with enhanced electron transfer between Ru nanoparticles and single atoms dispersed on a carbon substrate. Angew. Chem. Int. Ed. **60**(29), 16044–16050 (2021). <https://doi.org/10.1002/anie.202103557>
12. W. Zitzmann, A.S. Nair, B. Paulus, A. Rafferty, X. Yi et al., The role of nickel hydroxide phases in wastewater electrolysis for sustainable green hydrogen production. Nanoscale (2026). <http://doi.org/10.1039/D5NR05037E>
13. H.-S. Hu, Y. Li, Y.-R. Shao, K.-X. Li, G. Deng et al., NiCoP nanorod arrays as high-performance bifunctional electrocatalyst for overall water splitting at high current densities. J. Power Sources **484**, 229269 (2021). <https://doi.org/10.1016/j.jpowsour.2020.229269>
14. L. Yang, J. Lang, W. Jiang, Y. Ma, X. Chu et al., Interfacial engineering for the construction of iron sulfide/boride nanosheets heterostructure bifunctional electrocatalysts for high-efficiency overall water splitting. J. Alloys Compd. **1006**, 176344 (2024). <https://doi.org/10.1016/j.jallcom.2024.176344>
15. H. Ma, Y. Jin, X. Zhou, Y. Cui, Y. Zhao et al., Chloride-induced easier phase transformation and catalytic synergy for enhanced seawater splitting. Chem. Sci. (2026). <http://doi.org/10.1039/D5SC09403H>
16. A. Li, X. Qian, M. Han, Z. Li, X. He et al., Pyrolysis of ZIF-Co assisted by a small amount of KNO_3_: Tuning of Co-CoOx@N, O Co-doped carbon hybrid nanostructures for accelerating water splitting. Fuel **371**, 131969 (2024). <https://doi.org/10.1016/j.fuel.2024.131969>
17. L. Li, D. Liu, Z. Yu, Y. Cao, C. Zhong et al., Dynamic evolution of high-valence metals in high-entropy systems enabling highly enhanced oxygen evolution reaction. J. Energy Chem. **115**, 595–605 (2026). <https://doi.org/10.1016/j.jechem.2025.12.008>
18. T. Li, Z. Wang, Y. Wang, X. He, J. Luo et al., Anodized bulk FeCoCrNiTi_0.6_ high-entropy alloy electrocatalysts for long-term industrial-scale oxygen evolution reaction. J. Colloid Interface Sci. **703**, 139232 (2026). <https://doi.org/10.1016/j.jcis.2025.139232>
19. J. Fan, X. Ma, J. Xia, L. Zhang, Q. Bi et al., Corrosion resistance and earth-abundance FeS-based heterojunction catalyst for seawater splitting at industrial grade density. J. Colloid Interface Sci. **657**, 393–401 (2024). <https://doi.org/10.1016/j.jcis.2023.12.001>
20. L. Li, L. Qiao, D. Liu, Z. Yu, K. An et al., High-valence metals accelerate the reaction kinetics for boosting water oxidation. Small **21**(6), 2403992 (2025). <https://doi.org/10.1002/smll.202403992>
21. R.-Z. Sun, X. Ma, K. Chen, J.-B. Yang, Y.-X. Liu et al., Self-assembled polyoxometalate supramolecular nanosheets for efficient and durable water oxidation. Angew. Chem. Int. Ed. **64**(40), e202513915 (2025). <https://doi.org/10.1002/anie.202513915>
22. S. Haghverdi Khamene, M. Creatore, M.N. Tsampas, 3D electrode surface engineering *via* atomic layer deposition of nickel oxide for improved water oxidation performance. Chem. Eng. J. Adv. **22**, 100723 (2025). <https://doi.org/10.1016/j.ceja.2025.100723>
23. H. Shin, J. Lee, D. Jang, Y. Kim, H. Jeong et al., Enhanced oxygen evolution reaction and durability of carbon dot-anchored nickel-iron oxyhydroxide electrodes for alkaline water electrolysis at high current densities. Mater. Today Adv. **25**, 100560 (2025). <https://doi.org/10.1016/j.mtadv.2025.100560>
24. J. Xiang, F. Zhou, X. Ma, J. Wu, C. Guo et al., Spherical cluster heterojunction engineering of NiFeP/g-C_3_N_4_ for efficient oxygen evolution reaction in alkaline solution. J. Colloid Interface Sci. **674**, 266–278 (2024). <https://doi.org/10.1016/j.jcis.2024.06.042>
25. B. Kan, M. Wei, Y. Zhang, W. Feng, C. Si et al., Crystalline-amorphous NiFeP alloy as an efficient bifunctional self-supporting electrode for alkaline overall water splitting. J. Alloys Compd. **1060**, 187350 (2026). <https://doi.org/10.1016/j.jallcom.2026.187350>
26. Y. Zhou, J. Chen, Q. Chen, X. Dong, X. Yang et al., Sn-mediated amorphous NiFeP electroless plating on nickel mesh for stable high-current oxygen evolution reaction. ACS Appl. Mater. Interfaces **18**(10), 15144–15155 (2026). <https://doi.org/10.1021/acsami.5c24462>
27. X. Zhang, D. Lin, R. Wan, Y. Wang, Q. Pan et al., Hierarchical NiFeP nanoflowers on the MXene film as a self-standing bifunctional electrode toward superior overall water electrolysis. ACS Appl. Nano Mater. **7**(7), 7684–7693 (2024). <https://doi.org/10.1021/acsanm.4c00379>
28. M. Vinu, K.-Y. Chiang, Highly efficient oxygen carrier NiFeP (oxy) hydroxides nanoparticle embedded in N-doped porous carbon derived from bio-waste for bifunctional electrocatalysts. Chemosphere **366**, 143486 (2024). <https://doi.org/10.1016/j.chemosphere.2024.143486>
29. E. Jang, J. Kim, J. Cho, J. Lee, J. Kim, Defect modulation and *in-situ* exsolution in Y_2_Ru_2_O_7_@NiFeP/Ru heterostructure for enhanced oxygen evolution reaction. Rare Met. **44**(2), 1014–1023 (2025). <https://doi.org/10.1007/s12598-024-03006-y>
30. L. Yang, L. Li, Atomic-level La-modulated NiFeP bifunctional electrocatalysts for high efficiency overall water splitting. Chemistry **31**(46), e01525 (2025). <https://doi.org/10.1002/chem.202501525>
31. G.-L. Li, Y.-Y. Miao, F. Deng, S. Wang, R.-X. Wang et al., Highly-dispersed 2D NiFeP/CoP heterojunction trifunctional catalyst for efficient electrolysis of water and urea. J. Colloid Interface Sci. **667**, 543–552 (2024). <https://doi.org/10.1016/j.jcis.2024.04.059>
32. H. Chen, B. Hui, Flexible NiFeP@filter paper electrode for alkaline overall water electrosplitting. Int. J. Hydrog. Energy **51**, 615–623 (2024). <https://doi.org/10.1016/j.ijhydene.2023.08.279>
33. J. Chen, Q. Long, K. Xiao, T. Ouyang, N. Li et al., Vertically-interlaced NiFeP/MXene electrocatalyst with tunable electronic structure for high-efficiency oxygen evolution reaction. Sci. Bull. **66**(11), 1063–1072 (2021). <https://doi.org/10.1016/j.scib.2021.02.033>
34. M. Han, H. Wang, J. Zhou, K. Liu, N. Wang et al., Introducing lewis base-phosphate to boost neutral seawater splitting in anion exchange membrane electrolyzer. Adv. Funct. Mater. **35**(6), 2415143 (2025). <https://doi.org/10.1002/adfm.202415143>
35. H. Liang, A.N. Gandi, C. Xia, M.N. Hedhili, D.H. Anjum et al., Amorphous NiFe-OH/NiFeP electrocatalyst fabricated at low temperature for water oxidation applications. ACS Energy Lett. **2**(5), 1035–1042 (2017). <https://doi.org/10.1021/acsenergylett.7b00206>
36. A. Kafle, M. Kumar, D. Gupta, T.C. Nagaiah, The activation-free electroless deposition of NiFe over carbon cloth as a self-standing flexible electrode towards overall water splitting. J. Mater. Chem. A **9**(43), 24299–24307 (2021). <http://doi.org/10.1039/D1TA03244E>
37. Y.J. Lee, S.-K. Park, Metal-organic framework-derived hollow CoS_x_ nanoarray coupled with NiFe layered double hydroxides as efficient bifunctional electrocatalyst for overall water splitting. Small **18**(16), e2200586 (2022). <https://doi.org/10.1002/smll.202200586>
38. Z. Wang, W. Liu, Y. Hu, L. Xu, M. Guan et al., An Fe-doped NiV LDH ultrathin nanosheet as a highly efficient electrocatalyst for efficient water oxidation. Inorg. Chem. Front. **6**(7), 1890–1896 (2019). <http://doi.org/10.1039/C9QI00404A>
39. K. Fan, H. Chen, Y. Ji, H. Huang, P.M. Claesson et al., Nickel-vanadium monolayer double hydroxide for efficient electrochemical water oxidation. Nat. Commun. **7**, 11981 (2016). <https://doi.org/10.1038/ncomms11981>
40. S. Huang, J. Lu, X. Wu, H. Zhu, X. Shen et al., Ru-promoted NiFe oxyhydroxide anchored on the hierarchical porous N-doped carbon aerogel: Electronic structures modulation for much enhanced OER/HER dual-functional characteristics. Appl. Catal. A Gen. **664**, 119331 (2023). <https://doi.org/10.1016/j.apcata.2023.119331>
41. F. Liu, Z. Feng, X. Zhang, L. Cui, J. Liu, One-step achievement of Fe-doped and interfacial Ru nanoclusters co-engineered Ni(OH)_2_ electrocatalyst on Ni foam for promoted oxygen evolution reaction. J. Colloid Interface Sci. **638**, 498–505 (2023). <https://doi.org/10.1016/j.jcis.2023.01.037>
42. A.A. Pawar, H.A. Bandal, H. Kim, Spinel type Fe_3_O_4_ polyhedron supported on nickel foam as an electrocatalyst for water oxidation reaction. J. Alloys Compd. **863**, 158742 (2021). <https://doi.org/10.1016/j.jallcom.2021.158742>
43. Z. Gao, Z. Yu, Y. Huang, X. He, X. Su et al., Flexible and robust bimetallic covalent organic frameworks for the reversible switching of electrocatalytic oxygen evolution activity. J. Mater. Chem. A **8**(12), 5907–5912 (2020). <http://doi.org/10.1039/C9TA14023A>
44. Y. Zhang, X. Zhou, F. Zhang, T. Tian, Y. Ding et al., Design and synthesis of Cu modified cobalt oxides with hollow polyhedral nanocages as efficient electrocatalytic and photocatalytic water oxidation catalysts. J. Catal. **352**, 246–255 (2017). <https://doi.org/10.1016/j.jcat.2017.05.020>
45. M.S. Amer, M.A. Ghanem, P. Arunachalam, A.M. Al-Mayouf, S.M. Hadadi, Bifunctional electrocatalyst of low-symmetry mesoporous titanium dioxide modified with cobalt oxide for oxygen evolution and reduction reactions. Catalysts **9**(10): 836. (2019). <https://doi.org/10.3390/catal9100836>
46. L. Lin, M. Chen, L. Wu, Hierarchical MoP/NiFeP hybrid hollow spheres as highly efficient bifunctional electrocatalysts for overall water splitting. Mater. Chem. Front. **5**(1), 375–385 (2021). <http://doi.org/10.1039/D0QM00635A>
47. B. Weng, X. Wang, C.R. Grice, F. Xu, Y. Yan, A new metal–organic open framework enabling facile synthesis of carbon encapsulated transition metal phosphide/sulfide nanoparticle electrocatalysts. J. Mater. Chem. A **7**(12), 7168–7178 (2019). <http://doi.org/10.1039/C9TA00404A>
48. S. Wang, J. Cai, C. Lv, C. Hu, H. Guan et al., General and scalable preparation of Prussian blue analogues on arbitrary conductive substrates and their derived metal phosphides as highly efficient and ultra-long-life bifunctional electrocatalysts for overall water splitting. Chem. Eng. J. **420**, 129972 (2021). <https://doi.org/10.1016/j.cej.2021.129972>
49. Y. Wang, P. Yang, Y. Gong, D. Liu, S. Liu et al., Amorphous high-valence Mo-doped NiFeP nanospheres as efficient electrocatalysts for overall water-splitting under large-current density. Chem. Eng. J. **468**, 143833 (2023). <https://doi.org/10.1016/j.cej.2023.143833>
50. X. Luo, Q. Shao, Y. Pi, X. Huang, Trimetallic molybdate nanobelts as active and stable electrocatalysts for the oxygen evolution reaction. ACS Catal. **9**(2), 1013–1018 (2019). <https://doi.org/10.1021/acscatal.8b04521>
51. X. Zhang, X.-R. Shi, P. Wang, Z. Bao, M. Huang et al., Bio-inspired design of NiFeP nanoparticles embedded in (N, P) Co-doped carbon for boosting overall water splitting. Dalton Trans. **52**(20), 6860–6869 (2023). <http://doi.org/10.1039/D3DT00583F>
52. E. Vijayakumar, S. Ramakrishnan, C. Sathiskumar, D.J. Yoo, J. Balamurugan et al., MOF-derived CoP-nitrogen-doped carbon@NiFeP nanoflakes as an efficient and durable electrocatalyst with multiple catalytically active sites for OER, HER, ORR and rechargeable zinc-air batteries. Chem. Eng. J. **428**, 131115 (2022). <https://doi.org/10.1016/j.cej.2021.131115>
53. J. Bian, Z. Song, X. Li, Y. Zhang, C. Cheng, Nickel iron phosphide ultrathin nanosheets anchored on nitrogen-doped carbon nanoflake arrays as a bifunctional catalyst for efficient overall water splitting. Nanoscale **12**(15), 8443–8452 (2020). <http://doi.org/10.1039/C9NR10471B>
54. X. Yu, X. He, R. Li, X. Gou, One-step synthesis of amorphous nickel iron phosphide hierarchical nanostructures for water electrolysis with superb stability at high current density. Dalton Trans. **50**(23), 8102–8110 (2021). <http://doi.org/10.1039/D1DT00852H>
55. J.O. Silva, S. Cartagena, J.A. Calderón, Novel electrodeposited NiFeP/Zn bifunctional catalytic coating for alkaline water splitting. Electrochim. Acta **451**, 142299 (2023). <https://doi.org/10.1016/j.electacta.2023.142299>
56. Y. Liu, P. Ge, Y. Li, X. Zhai, K. Lu et al., Prussian blue analogues derived Fe-NiCoP reveals the cooperation of Fe doping and phosphating for enhancing OER activity. Appl. Surf. Sci. **615**, 156378 (2023). <https://doi.org/10.1016/j.apsusc.2023.156378>
